# Supplementary material for: mRNA vaccines induce durable immune memory to SARS-CoV-2 and variants of concern
Source: Science. 2021 Oct 14;374(6572):abm0829. doi: 10.1126/science.abm0829 (PMC9284784; doi:10.1126/science.abm0829)
Supplement: 20211014-1 [file science.abm0829.v1.pdf]

Cite as: R. R. Goel *et al.*, *Science*
10.1126/science.abm0829 (2021).

mRNA vaccines induce durable immune memory to SARS-CoV-2 and variants of concern

Rishi R. Goel[1,2]†, Mark M. Painter[1,2]†, Sokratis A. Apostolidis[1,2,3]†, Divij Mathew[1,2]†, Wenzhao Meng[1,4], Aaron M. Rosenfeld[1,4], Kendall A. Lundgreen[5], Arnold Reynaldi[6], David S. Khoury[6], Ajinkya Pattekar[2], Sigrid Gouma[5], Leticia Kuri-Cervantes[1,5], Philip Hicks[5], Sarah Dysinger[5], Amanda Hicks[2], Harsh Sharma[2], Sarah Herring[2], Scott Korte[2], Amy E. Baxter[1], Derek A. Oldridge[1,4], Josephine R. Giles[1,7,8], Madison E. Weirick[5], Christopher M. McAllister[5], Moses Awofolaju[5], Nicole Tanenbaum[5], Elizabeth M. Drapeau[5], Jeanette Dougherty[1], Sherea Long[1], Kurt D'Andrea[1], Jacob T. Hamilton[2,5], Maura McLaughlin[1], Justine C. Williams[2], Sharon Adamski[2], Oliva Kuthuru[1], The UPenn COVID Processing Unit‡, Ian Frank[9], Michael R. Betts[1,5], Laura A. Vella[10], Alba Grifoni[11], Daniela Weiskopf[11], Alessandro Sette[11,12], Scott E. Hensley[5], Miles P. Davenport[6], Paul Bates[5], Eline T. Luning Prak[1,4], Allison R. Greenplate[1,2], E. John Wherry[1,2,7,8]*

[1]Institute for Immunology, University of Pennsylvania Perelman School of Medicine, Philadelphia, PA, USA. [2]Immune Health, University of Pennsylvania Perelman School of Medicine, Philadelphia, PA, USA. [3]Division of Rheumatology, University of Pennsylvania Perelman School of Medicine, Philadelphia, PA, USA. [4]Department of Pathology and Laboratory Medicine, University of Pennsylvania Perelman School of Medicine, Philadelphia, PA, USA. [5]Department of Microbiology, University of Pennsylvania Perelman School of Medicine, Philadelphia, PA, USA. [6]Kirby Institute, University of New South Wales, Sydney, Australia. [7]Department of Systems Pharmacology and Translational Therapeutics, University of Pennsylvania Perelman School of Medicine, Philadelphia, PA, USA. [8]Parker Institute for Cancer Immunotherapy, University of Pennsylvania Perelman School of Medicine, Philadelphia, PA, USA. [9]Division of Infectious Disease, University of Pennsylvania Perelman School of Medicine, Philadelphia, PA, USA. [10]Division of Infectious Disease, Department of Pediatrics, Children's Hospital of Philadelphia, Philadelphia, PA, USA. [11]Center for Infectious Disease and Vaccine Research, La Jolla Institute for Immunology (LJI), La Jolla, CA, USA. [12]Department of Medicine, Division of Infectious Diseases and Global Public Health, University of California San Diego (UCSD), La Jolla, CA, USA.

†These authors contributed equally to this work.

‡The UPenn COVID Processing Unit includes individuals from diverse laboratories at the University of Pennsylvania who volunteered their time and effort to enable study of COVID-19 patients during the pandemic. Members and affiliations are listed at the end of this paper.

***Corresponding author. Email: wherry@pennmedicine.upenn.edu**

The durability of immune memory after SARS-CoV-2 mRNA vaccination remains unclear. Here, we longitudinally profiled vaccine responses in SARS-CoV-2 naïve and recovered individuals for 6 months after vaccination. Antibodies declined from peak levels but remained detectable in most subjects at 6 months. We found mRNA vaccines generated functional memory B cells that increased from 3-6 months post-vaccination, with the majority of these cells cross-binding the Alpha, Beta, and Delta variants. mRNA vaccination further induced antigen-specific CD4+ and CD8+ T cells, and early CD4+ T cell responses correlated with long-term humoral immunity. Recall responses to vaccination in individuals with pre-existing immunity primarily increased antibody levels without substantially altering antibody decay rates. Together, these findings demonstrate robust cellular immune memory to SARS-CoV-2 and variants for at least 6 months after mRNA vaccination.

The coronavirus disease 2019 (COVID-19) pandemic has resulted in significant morbidity and mortality worldwide. Community-level immunity, acquired through infection or vaccination, is necessary to control the pandemic as the virus continues to circulate (*1*). mRNA vaccines encoding a stabilized version of the full-length SARS-CoV-2 Spike protein have been widely administered and clinical trial data demonstrated up to 95% efficacy in preventing symptomatic COVID-19 (*2*, *3*). These mRNA vaccines induce potent humoral immune responses, with neutralizing antibody titers proposed as the major correlate of protection (*4*–*6*). Current evidence suggests that circulating antibodies persist for at least 6 months post-vaccination (*7*), though there is some decay from peak levels achieved after the second dose. This decline from peak antibody levels may be associated with an increase in infections over time compared to the initial months post-vaccination (*8*, *9*). Yet, vaccine-induced immunity remains highly effective at preventing severe disease, hospitalization, and death even at later timepoints when antibody levels may decline (*10*–*12*).

Previous research has largely focused on responses early in the course of vaccination, with transcriptional analysis identifying potential links between myeloid cell responses and neutralizing antibodies (*13*). In addition to the

production of antibodies, an effective immune response requires the generation of long-lived memory B and T cells. mRNA vaccines induce robust germinal center responses in humans (*14*, *15*), resulting in memory B cells that are specific for both the full-length SARS-CoV-2 Spike protein and the Spike receptor binding domain (RBD) (*16*–*18*). mRNA vaccination has also been shown to generate Spike-specific memory CD4+ and CD8+ T cell responses (*19*–*22*). Although antibodies are often correlates of vaccine efficacy, memory B cells and memory T cells are important components of the recall response to viral antigens and are a likely mechanism of protection, especially in the setting of exposures in previously vaccinated individuals where antibodies alone do not provide sterilizing immunity (*23*). In such cases, memory B and T cells can be rapidly re-activated, resulting in enhanced control of initial viral replication and limiting viral dissemination in the host (*24*, *25*). By responding and restricting viral infection within the first hours to days after exposure, cellular immunity can thereby reduce or even prevent symptoms of disease (i.e., preventing hospitalization and death) and potentially reduce the ability to spread virus to others (*26*, *27*).

Immunological studies of SARS-CoV-2 infection show that memory B and T cell responses appear to persist for at least 8 months post-symptom onset (*28*, *29*). However, the durability of these populations of memory B and T cells following vaccination remains poorly understood. The emergence of several SARS-CoV-2 variants, including B.1.1.7 (Alpha), B.1.351 (Beta), and B.1.617.2 (Delta), has also raised concerns about increased transmission and potential evasion from vaccine-induced immunity (*30*–*33*). As such, it is necessary to develop a more complete understanding of the trajectory and durability of immunological memory after mRNA vaccination, as well as how immune responses are affected by current variants of concern (VOCs). Moreover, the United States and other well-resourced countries have recently announced plans for a third vaccine booster dose, yet information on how pre-existing serological and cellular immunity to SARS-CoV-2 is boosted by mRNA vaccination remains limited. Specifically, it is unclear how different components of the immune response may benefit from boosting and whether boosting has any effect on the durability of these components. Here, we investigated these key questions by measuring SARS-CoV-2-specific antibody, memory B cell, and memory T cell responses through 6 months post-vaccination in a group of healthy subjects generating primary immune responses to 2 doses of mRNA vaccine compared with a group of SARS-CoV-2 recovered vaccinees generating recall responses from pre-existing immunity. These analyses provide insights into mRNA vaccine-induced immunological memory and may be relevant for future vaccine strategies, including recommendations for additional booster vaccine doses.

Results and Discussion

Cohort Design

We collected 348 longitudinal samples from 61 individuals receiving either the Pfizer BNT162b2 (N=54) or Moderna mRNA-1273 (N=7) SARS-CoV-2 vaccines at 6 timepoints (Fig. 1A), ranging from pre-vaccination baseline to 6 months post-vaccination. This study design allowed us to monitor the induction and maintenance of antigen-specific immune responses to the vaccine. Specifically, sampling at 1-, 3-, and 6-months post-vaccination enabled analysis of immune trajectories from peak responses after the second vaccine dose through establishment and maintenance of immunological memory. This cohort was divided into 2 groups based on prior SARS-CoV-2 infection (N=45 SARS-CoV-2 naïve, N=16 SARS-CoV-2 recovered). Age and sex were balanced in both groups. Paired serum and peripheral blood mononuclear cell (PBMC) samples were collected from all individuals, allowing detailed analysis of both serologic and cellular immune memory to SARS-CoV-2 antigens. Notably, the subjects with a prior infection allowed us to study the dynamics of reactivating pre-existing immunity with mRNA vaccines. Though pre-existing immunity generated by infection may differ from that generated by vaccination, responses observed in this group may provide insights into boosting of vaccine-induced immunity using additional doses of vaccine.

Antibody Responses to SARS-CoV-2 mRNA Vaccines

We first measured anti-Spike and anti-RBD binding antibody responses in plasma samples by enzyme linked immunosorbent assay (ELISA). As reported previously by our group and others, mRNA vaccines induced robust circulating antibody responses to the SARS-CoV-2 Spike protein and Spike RBD with distinct patterns of early response in SARS-CoV-2 naïve and recovered individuals (Fig. 1B) (*16*, *34*–*36*). Peak levels of anti-Spike and anti-RBD IgG were observed 1 week after the second vaccine dose and subsequently declined over the course of the next 2 months with a half-life of ~28-33 days (Fig. 1B), consistent with the dynamics of a typical immune response. This decrease in antibody levels slowed from 3-6 months post-vaccination (decay rates were significantly different before and after day 89 by likelihood ratio rest; $p = 0.004$ for anti-Spike IgG, $p = 0.01$ for anti-RBD IgG) (Fig. 1B). Of note, the calculated decay rates for anti-Spike IgG were not significantly different between SARS-CoV-2 naïve and recovered vaccinees. Even after the decrease from peak antibody responses, all individuals had detectable anti-Spike IgG at 6 months.

To examine the functional quality of circulating antibodies, we used a neutralization assay with pseudotyped virus expressing either the wild-type Spike with the prevailing D614G mutation or the B.1.351 variant Spike (sequences in Methods). We focused on B.1.351 neutralization as this

variant has consistently shown the highest immune evasion among the current VOCs. In line with our binding antibody data, neutralizing titers for D614G and B.1.351 declined from peak levels after the second dose to 6 months for both SARS-CoV-2 naïve and recovered vaccinees (Fig. 1C). However, neutralizing titers displayed different decay kinetics, with slightly longer half-lives than binding antibody responses. Modeled 2-phase decay rates for D614G neutralization were not significantly different between SARS-CoV-2 naïve and recovered vaccinees with a half-life of 72 days between 3-6 months post-vaccination (Fig. 1C). In contrast, a relative stabilization of neutralizing titers against the B.1.351 variant was observed between 3 and 6 months post-vaccination in individuals without a prior SARS-CoV-2 infection with a half-life of 231 days, compared to 63 days in SARS-CoV-2 recovered subjects (Fig. 1C). We next compared neutralizing titers to D614G, B.1.351, and B.1.617.2 at 6 months post-vaccination. Neutralizing antibody titers to B.1.617.2 were similar to D614G (Fig. 1D). By contrast, neutralizing titers to B.1.351 were significantly lower than D614G. Despite this reduced neutralizing ability, 31/33 SARS-CoV-2 naïve and 9/9 SARS-CoV-2 recovered individuals still had neutralizing antibodies against B.1.351 above the limit of detection at 6 months post-vaccination (Fig. 1, C and D). Finally, cross-sectional analysis of 6-month antibody responses also demonstrated that binding antibodies remained highly correlated with neutralizing titers (Fig. 1E), indicating that Spike- and RBD-specific antibody responses retain their functional characteristics and neutralizing capacity over time.

Memory B Cell Responses to SARS-CoV-2 mRNA Vaccines

In addition to antibodies, we measured the frequencies of SARS-CoV-2 Spike- and RBD-specific memory B cells in peripheral blood using a flow cytometric assay. Antigen specificity was determined based on binding to fluorescent SARS-CoV-2 Spike and RBD probes (Fig. 2, A and B). Influenza hemagglutinin (HA) from the 2019 flu vaccine season was also included as a historical antigen control. Full gating strategies are provided in fig. S1A.

SARS-CoV-2-specific memory B cells were detectable in all previously uninfected individuals after 2 vaccine doses (the currently recommended primary vaccination series) and remained stable as a percentage of total B cells from 1-3 months post-vaccination (Fig. 2C). All SARS-CoV-2 recovered individuals in our study had a robust population of antigen-specific memory B cells at pre-vaccination baseline, and these pre-existing memory B cells were significantly boosted by the first vaccine dose with little change after the second vaccine dose (Fig. 2C). No changes were observed in influenza HA+ memory B cells after SARS-CoV-2 vaccination for either group (Fig. 2C).

Longitudinal analysis revealed a continued increase in the frequency of Spike+ and Spike+ RBD+ memory B cells from 3-6 months post-vaccination in SARS-CoV-2 naïve individuals, whereas the frequency of these antigen-specific memory B cells in SARS-CoV-2 recovered subjects continued to decline from peak levels (Fig. 2C). One possible explanation for the observed increase in frequency of vaccine-induced memory B cells over time in SARS-CoV-2 naïve vaccinees is prolonged germinal center activity, resulting in continued export of memory B cells. Indeed, antigen-specific germinal center B cells have been documented in axillary lymph nodes at 15 weeks post-mRNA vaccination in SARS-CoV-2 naïve subjects (*14*), though germinal center dynamics in vaccinees with prior immunity to SARS-CoV-2 remain to be defined. SARS-CoV-2 recovered individuals had consistently higher frequencies of antigen-specific memory B cells up to 3 months post-vaccination (Fig. 2C). However, due to distinct trajectories, both SARS-CoV-2 naïve and SARS-CoV-2 recovered individuals had similar frequencies of Spike+ and Spike+ RBD+ memory B cells at 6 months post-vaccination (Fig. 2C), perhaps reflecting some upper limit to the frequencies of antigen-specific memory B cells that can be maintained long-term.

We next investigated the phenotype of mRNA vaccine-induced memory B cells. Analysis of immunoglobulin isotypes in SARS-CoV-2 naïve vaccinees revealed a steady increase in IgG+ memory B cells over time (Fig. 2, D and E, and fig. S2, A to C), indicating ongoing class-switching. By contrast, IgM+ cells were most abundant at pre-immune baseline and early post-vaccination timepoints. IgM+ and IgA+ memory B cells represented a minor fraction of the overall response in the blood at later timepoints (Fig. 2F and fig. S2C). In SARS-CoV-2 recovered vaccinees, the majority of Spike+ and Spike+ RBD+ memory B cells were IgG+ at baseline, and the fraction of IgG+ cells continued to increase following vaccination (Fig. 2, D and E, and fig. S2, A to C). Moreover, we assessed the activation status of antigen-specific memory B cells by CD71 expression (*37*). The percent of Spike+ memory B cells expressing CD71 increased over the course of the primary 2-dose vaccine regimen in SARS-CoV-2 naïve individuals, peaking at 1 week after the second vaccine dose (Fig. 2G). The percent of CD71+ antigen-specific memory B cells then steadily declined by the 6-month timepoint, indicating a transition toward a population of mature resting memory B cells. A similar decrease in CD71 expression was observed from 1-6 months post-vaccination in SARS-CoV-2 recovered individuals (Fig. 2G).

Given the robust generation of Spike- and RBD-binding memory B cells, we next tested whether vaccine-induced memory B cells could produce functional antibodies upon reactivation. For other pathogens, this reactivation-induced antibody production from memory B cells may be especially

relevant in the setting of antigen re-encounter, either through exposure to live virus or an additional vaccine dose (*38*). To this end, we established an in vitro culture system to differentiate memory B cells into antibody secreting cells (*39*). PBMC samples from vaccinated individuals at the 6-month timepoint were cultured with a combination of R848, a TLR7/8 agonist, and IL-2, and culture supernatants were collected to measure antibody levels and function (Fig. 2H). Anti-Spike IgG was detected in supernatants as early as 4 days post-stimulation (Fig. 2I), indicating that memory B cells can act as a rapid source of secondary antibody production. All 6-month samples tested generated significant levels of anti-Spike IgG in this assay compared to unstimulated controls (Fig. 2J). This in vitro anti-Spike IgG production also correlated with the frequency of Spike+ memory B cells detected by flow cytometry (Fig. 2K). We further tested the function of memory B cell-derived antibodies from culture supernatants using an ELISA-based RBD-ACE2-binding inhibition assay. Indeed, RBD-ACE2-binding inhibition activity was observed and correlated with the frequency of RBD-specific memory B cells in peripheral blood (Fig. 2L). Moreover, pseudovirus neutralization assays demonstrated that antibodies produced by memory B cells upon restimulation were capable of neutralizing the B.1.351 and B.1.617.2 VOCs (Fig. 2M), and neutralization titers correlated with both anti-Spike IgG and RBD-ACE2 binding inhibition (fig. S3, A to D). The neutralization potential of memory B cell-derived antibodies was greater for B.1.617.2 than B.1.351 but was not significantly different between SARS-CoV-2 naïve and recovered vaccinees. Finally, VOC neutralizing titers in culture supernatants correlated with the frequency of RBD-specific memory B cells by flow cytometry (Fig. 2, N and O), further supporting the functional relevance of quantifying antigen-specific memory B cells in the blood. Taken together, these data demonstrate that mRNA vaccines induced a population of memory B cells that were durable for at least 6 months after vaccination and were capable of rapidly producing functional antibodies against SARS-CoV-2, including neutralizing antibodies against VOCs, upon stimulation.

Memory B Cell Responses to Major Variants of Concern (VOCs)

We next developed an expanded antigen probe panel to better quantify memory B cell specificities to different regions of the Spike protein and test how RBD binding by memory B cells may be affected by the mutations found in emerging VOCs. Specifically, we designed B cell tetramers for 8 SARS-CoV-2 antigens, including full-length Spike, N-terminal domain (NTD), multiple variant RBDs (wild-type, B.1.1.7, B.1.351, and B.1.617.2), and the S2 domain (Fig. 3, A and B). Spike-specific memory B cells were defined based on a multiple-discrimination approach, with binding to full-length Spike plus one or more additional probes. This strategy also allowed us to identify memory B cells that cross-bind all variant RBDs (RBD++++). SARS-CoV-2 nucleocapsid was used as a vaccine-irrelevant antigen (but one for which SARS-CoV-2 immune subjects had detectable pre-existing immunity; fig. S4, A and B). Full gating strategies are provided in fig. S1B. We also leveraged a separate cohort of healthcare workers (HCW, table S1) who had mild COVID-19 and were sampled longitudinally after a positive serology test to compare vaccine-induced responses with infection alone (*40*).

mRNA vaccination induced robust memory B cell responses to all SARS-CoV-2 Spike antigens in previously uninfected individuals, and the frequency of these memory B cells increased from 3-6 months post-vaccination (Fig. 3C). In individuals with immunity from prior COVID-19, vaccination resulted in a significant expansion of memory B cells targeting all Spike antigens. These responses subsequently contracted from peak levels, remaining slightly above pre-vaccination frequencies at 6 months post-vaccination (Fig. 3C). In the mild infection HCW cohort, a gradual increase in the frequency of Spike+ NTD+ and Spike+ RBD++++ memory B cells was observed from 2 weeks to 6 months post-seropositive test (Fig. 3C). Cross-sectional analysis at 6 months post-vaccination or sero-positivity revealed similar antigen-specific memory B cell frequencies between all groups (fig. S4B), suggesting that both vaccination and infection can induce durable memory B cell populations.

As our panel included probes covering much of the Spike protein, including NTD, RBD, and S2, we also examined immunodominance patterns and how B cell immunodominance to Spike changed over time. In previously uninfected individuals, ~30% of Spike-binding memory B cells co-bound S2 at pre-vaccine baseline (Fig. 3D). Previous work has shown that the S2 domain of SARS-CoV-2 Spike is more conserved with other coronaviruses and it is likely that S2-binding memory B cells detected at baseline reflect cross-reactivity to these commonly circulating coronaviruses (*41*, *42*). mRNA vaccination induced robust populations of S2-specific memory B cells in SARS-CoV-2 naïve vaccinees, with S2-binding B cells accounting for 40-80% of the total Spike-specific memory B cell population at 6 months (Fig. 3D). Although the overall frequency of NTD+ and RBD+ memory B cells increased over time, they were comparatively less immunodominant than S2 as a percentage of total Spike+ memory B cells (Fig. 3, C and D). mRNA vaccination induced a gradual increase in NTD-specificity over time in SARS-CoV-2 naïve individuals, whereas RBD-specificity as a percent of Spike+ memory B cells had a more prominent peak 1 week after the second vaccine dose and then stabilized from 3-6 months post-vaccination (Fig. 3D). When SARS-CoV-2 recovered subjects were immunized with mRNA vaccine, a similar immunodominance pattern was observed with S2-specificity representing

most of the total anti-Spike response (Fig. 3D). Vaccination transiently increased NTD- and RBD-specificity in this group; however, this effect returned to baseline by 6 months post-vaccination. In the context of infection only, we found that NTD, RBD and S2 immunodominance remained relatively stable from early convalescence through late memory, with a slight increase in NTD-specificity over time (Fig. 3D).

We next examined memory B cell binding to B.1.1.7 (Alpha), B.1.351 (Beta), and B.1.617.2 (Delta) variant RBDs relative to WT RBD (Fig. 3, E and F, and fig. S4, C and D). All RBD probes were used at the same concentration to facilitate direct comparisons, and specific point mutations are shown in Fig. 3, A and B. Variant-binding memory B cells were detectable in all SARS-CoV-2 naïve individuals after 2 vaccine doses and were stable as a percentage of WT RBD+ cells from 1-6 months post vaccination (Fig. 3F). In SARS-CoV-2 recovered individuals, vaccination resulted in a significant increase in memory B cell cross-binding to the B.1.617.2 variant (Fig. 3F). In convalescent individuals who recovered from a mild infection, there was a gradual increase in cross-binding to variants over time (Fig. 3F). Class-switching to an IgG dominated response was also observed in all groups, with vaccination producing a higher percentage of IgG+ cells compared to infection alone (fig. S4, E and F). Of note, the variants and corresponding mutations tested in our panel had different magnitudes of effect (Fig. 3, E and F, and fig. S4, C and D). B.1.1.7 RBD with a single N501Y mutation had relatively little change in binding compared to WT RBD. Consistent with the in vitro pseudovirus neutralization data above, B.1.351 RBD resulted in a more substantial loss of cross-binding, whereas B.1.617.2 RBD had an intermediate effect on binding.

Cross-sectional analysis of variant-binding at the 6-month timepoint also revealed two major findings. First, all vaccinated individuals in our study maintained variant-specific memory B cells for at least 6 months, with an average of >50% of WT RBD+ memory B cells also cross-binding all 3 major variants of concern (Fig. 3, G and H). Second, mRNA vaccination in SARS-CoV-2 naïve individuals induced a stronger response to B.1.351 than infection alone (Fig. 3H). One possible explanation for this difference is the immunogen itself. Vaccinated individuals mount a primary response to the mRNA-encoded prefusion stabilized Spike trimer, potentially allowing increased recruitment and/or selection of specific clones that can bind conserved regions of RBD (*43*, *44*). In contrast, convalescent individuals were primed against native, non-stabilized Spike protein. Taken together, our data indicate robust B cell memory to multiple components of the Spike protein as well as currently described VOCs that continues to evolve and increase in frequency over time.

Clonal Evolution of Variant-Specific Memory B Cells

We next asked what differences may underly variant-binding versus non-binding properties of memory B cells. Here, we focused on the Beta B.1.351 variant RBD containing the K417N, E484K, and N501Y mutations as this variant resulted in the greatest loss of binding relative to WT RBD (Fig. 3, E, G, and H). We designed a sorting panel to identify 3 populations of memory B cells with different antigen-binding specificities: 1) memory B cells that bind full-length Spike but not RBD, 2) memory B cells that bind full-length Spike and WT RBD but not B.1.351 variant RBD, and 3) memory B cells that bind full-length Spike and cross-bind both WT and B.1.351 variant RBD (Fig. 4A and fig. S5A). Naïve B cells were also sorted as a control. These populations were isolated from 8 SARS-CoV-2 naïve and 4 SARS-CoV-2 recovered individuals at 3-4 months post-vaccination (Fig. 4A and fig. S5A). Consistent with our previous data, between 50-80% of WT RBD+ cells co-bound B.1.351 variant RBD (Fig. 4B), indicating that a majority of RBD epitopes in the response are shared by the WT and mutant RBDs.

To gain insight into the clonal composition of the different spike and/or RBD-binding B cell populations, IgH rearrangements were amplified from the sorted populations (N=48 total) and related sequences were grouped into clones (N=348,346 clones, table S2). We analyzed the contribution of the top copy number clones to the overall repertoire as measured by the D20 index. The D20 index ranged from less than 1% for naïve B cells (which is expected for a diverse, non-clonally expanded population) to greater than 20% for some of the antigen-binding populations (Fig. 4C). Clones that cross-bound both WT and B.1.351 RBD trended toward higher D20 scores, suggesting greater clonal expansion and/or lower diversity compared to the other antigen-binding populations (Fig. 4C). The clonality of antigen-binding memory B cell populations was not significantly different after vaccination based on prior immunity, although there was heterogeneity in clonal expansion across individuals.

We further analyzed IGHV gene usage across the different antigen-binding memory B cell populations. Hierarchical clustering revealed that VH gene profiles were overall similar in vaccinated individuals regardless of prior SARS-CoV-2 infection status (Fig. 4D and fig. S5B), indicating that both vaccination and infection followed by vaccination can recruit similar clones into the response. Rather, IGHV gene usage largely clustered based on the antigen specificity, with increased usage of VH3-53 and VH3-66 in RBD cross-binding clones (Fig. 4D and fig. S5B). Of note, both of these IGHV genes are known to be enriched in spike-binding B cells (*45*, *46*). These differences in IGHV gene usage between WT only and variant cross-binding phenotype suggested that these cells may derive, at least partially, from different B cell clones that were independently recruited into the vaccine response.

Analysis of VH gene sequences also revealed clear differences in somatic hypermutation (SHM) between the different

antigen-binding populations. As expected, SARS-CoV-2-specific memory B cell clones had significantly more VH nucleotide mutations compared to naïve B cell clones (Fig. 4, E and F, and fig. S5C). Spike+, RBD non-binding memory B cells (which include NTD- and S2-binding populations) had high SHM (Fig. 4, E and F, and fig. S5C), consistent with germinal center-dependent responses as well as possible recall responses of pre-existing S2 cross-reactive clones. Notably, significantly higher levels of SHM were observed in variant RBD cross-binding clones compared to WT RBD only clones (Fig. 4, E and F, and fig. S5C). Additionally, boosting of infection-acquired immunity by mRNA vaccination in SARS-CoV-2 recovered donors did not produce higher SHM in RBD-binding memory B cell clones compared to vaccination alone (Fig. 4F).

To determine if variant cross-binding clones could evolve from WT RBD-binding clones, we next investigated if there was any clonal overlap between these populations. For clonal overlap analysis, we focused on larger clones (defined as having copy numbers at or above 50% of the mean copy number frequency within each sequencing library) (*47*), as larger clones are more readily sampled at both the clonal and subclonal levels. Among such larger clones, 2.5% had sequence variants that were isolated from both WT RBD and cross-binding populations (Fig. 4G and fig. S5D). Lineage analysis revealed that WT and cross-binding sequence variants localized on separate branches (representative lineages shown in Fig. 4H), indicating that the shift in antigen-reactivity was not due to contamination of the sorted populations (in which case sequence variants localize to the same nodes). Next, to determine if cross-binding activity arose from WT binding or vice versa, we used SHM as a molecular clock and counted the fraction of overlapping clonal lineages in which variant binding had higher, lower, or equivalent levels of SHM to WT RBD-binding variants. Consistent with the overall SHM data, this analysis of overlapping clones revealed higher levels of SHM in the variant binding sequences compared to WT only binding sequences (Fig. 4, I and J), suggesting a clonal evolution from WT only binding to variant RBD co-binding for at least some clones.

Taken together, these data indicate that mRNA vaccine-induced memory B cells that bind variant RBDs have higher SHM compared to clones that only bind WT RBD. Moreover, the clonal relationships between WT-only and cross-binding RBD-specific memory B cells suggest that variant binding capacity can evolve from clones that initially bound to WT RBD. Ongoing evolution and selection of these clones could therefore facilitate cross-protection against different VOCs. These findings are consistent with earlier work suggesting that SHM and affinity maturation are important for the acquisition of broader neutralization activity of RBD-binding antibodies that are formed in response to SARS-CoV-2 infection (*48*, *49*). It is presently unclear how additional antigen exposure through booster vaccination, environmental virus exposure, or overt infection may impact additional affinity maturation toward improved variant-binding.

Memory CD4+ and CD8+ T Cell Responses to SARS-CoV-2 mRNA Vaccines

In addition to antibodies and memory B cells, memory T cells can contribute to protection upon re-exposure to virus. Memory T cell responses have also been shown to be less affected by variants of concern than humoral immune responses (*21*, *50*). To determine whether mRNA vaccination induced durable antigen-specific memory T cell responses, we performed a flow cytometric analysis using an activation induced marker (AIM) assay. PBMCs were stimulated with peptide megapools containing optimized Spike epitopes (*51*, *52*). Antigen-specific responses were quantified as the frequency of AIM+ non-naïve T cells in stimulated samples with background subtraction from paired unstimulated controls (Fig. 5, A and B) (*19*). Full gating strategies are provided in fig. S6. Antigen-specific CD4+ T cells were defined based on co-expression of CD40L and CD200. Antigen-specific CD8+ T cells were defined based on expression of 4 of 5 total activation markers as described previously (*19*).

Consistent with recent studies, SARS-CoV-2 mRNA vaccination efficiently primed antigen-specific CD4+ T cells and CD8+ T cells (Fig. 5, C and D) (*20*–*22*). All individuals in our cohort, regardless of prior infection with SARS-CoV-2, had detectable CD4+ T cell responses above their individual baseline one week following the second vaccine dose (Fig. 5C). Most (36/41) SARS-CoV-2 naïve individuals also generated detectable CD8+ T cell responses after the second dose (Fig. 5D). In contrast, vaccination did little to further boost pre-vaccination antigen-specific CD8+ T cell frequencies in SARS-CoV-2 recovered individuals (Fig. 5D). A marked contraction phase was observed from peak responses to 3-months post-vaccination, with a half-life of 47 days for CD4+ T cells and 27 days for CD8+ T cells (Fig. 5, C and D). These kinetics are consistent with a typical T cell response after the effector phase (*53*). After this initial contraction, antigen-specific memory CD4+ T cell frequencies stabilized from 3-6 months post-vaccination with a half-life of 187 days, whereas CD8+ T cells continued to decline. Overall, 28/31 SARS-CoV-2 naïve individuals had vaccine-induced antigen-specific CD4+ T cell responses at 6 months post-vaccination above pre-vaccination baseline levels, and 13/31 had detectable CD8+ T cell responses above baseline (Fig. 5, C and D). In SARS-CoV-2 recovered subjects, mRNA vaccination had only a modest impact on T cell responses and did not elevate the magnitude of long-term antigen-specific CD4+ or CD8+ T cell memory above baseline levels (Fig. 5, C and D). Taken together, these data indicate that mRNA vaccination generates durable

SARS-CoV-2-specific CD4+ T cell memory in individuals who were not previously infected with SARS-CoV-2 and only transiently boosts these responses in SARS-CoV-2 recovered individuals.

Antigen-specific T cells can further be classified into different memory subsets using cell surface markers (Fig. 5E). Peak CD4+ T cell responses following SARS-CoV-2 mRNA vaccination were composed of predominantly central memory (CM; CD45RA- CD27+ CCR7+) and effector memory 1 (EM1; CD45RA- CD27+ CCR7-) cells in both SARS-CoV-2 naïve and recovered individuals (Fig. 5F) (*19*). During contraction from peak responses, antigen-specific CCR7+ CM cells were largely lost from circulation, whereas antigen-specific CCR7- EM1 cells stabilized in frequency from 3-6 months post-vaccination. Moreover, the percentage of the peak CD4+ response that was EM1 cells, but not other memory subsets, was significantly associated with the durability of the overall CD4+ T cell response at 3 and 6 months post-vaccination (Fig. 5, G and H), suggesting that EM1s are long-lived memory CD4+ T cells and that early skewing toward an EM1 phenotype contributes to durable CD4+ T cell memory. Although our AIM assay allows detection of low-frequency memory CD8+ T cell responses for overall quantification, reliable subsetting of antigen-specific CD8+ T cells at memory timepoints was not feasible due to the low number of events.

mRNA vaccination also preferentially induced antigen-specific CD4+ cTfh and Th1 helper cells in both SARS-CoV-2 naïve and recovered individuals, whereas Th2, Th17, and Th1/17 cells were detected at lower levels in the AIM assay (Fig. 5I). Although the overall frequency of antigen-specific CD4+ T cells stabilized from 3-6 months post-vaccination, cTfh and Th1 cells had distinct trajectories. Specifically, cTfh cells declined more rapidly than Th1 cells both during the initial contraction phase and from 3-6 months post-vaccination (Fig. 5J), perhaps reflecting redistribution of Tfh into lymphoid tissues. In contrast, Spike-specific Th1 cells did not decline in the blood from 3-6 months post-vaccination. While cTfh cells may be important in the early stages of vaccine response, these data indicate that the durable component of the memory CD4+ T cell response at 6 months post-vaccine is largely composed of Th1 cells, and boosting of pre-existing immunity with mRNA vaccine does not change the magnitude or subset composition of the CD4+ memory T cell response.

Integrated Analysis of Immune Components and Vaccine-Induced Memory to SARS-CoV-2

A goal of this study was to assess the development of multiple components of antigen-specific immune memory over time in the same individuals following SARS-CoV-2 mRNA vaccination. This dataset allowed us to integrate longitudinal antibody, memory B cell, and memory T cell responses to construct an immunological landscape of SARS-CoV-2 mRNA vaccination. To this end, we applied uniform manifold approximation and projection (UMAP) to visualize the trajectory of vaccine-induced adaptive immunity over time. This analysis revealed a continued evolution of the overall immune response in SARS-CoV-2 naïve subjects after mRNA vaccination with different timepoints occupying largely non-overlapping UMAP space (Fig. 6A). Projection of individual immune components onto the UMAP space revealed that primary vaccination was largely defined by rapid induction of CD4+ T cell immunity (Fig. 6B). The second vaccine dose induced peak antibody, CD4+ T cell, and CD8+ T cell responses. Antibodies and CD4+ T cells then remained durable through later memory timepoints, coinciding with a trajectory shift toward peak memory B cell responses. Notably, all 6-month samples clustered away from pre-immune baseline samples (Fig. 6A), highlighting the durable multi-component immune memory induced by mRNA vaccination. At 6 months, we observed some heterogeneity in the immune landscape. This heterogeneity may be partially driven by a significant negative correlation between age and anti-Spike IgG (fig. S7, A and B). Sex did not appear to have any association with the overall antigen-specific response to mRNA vaccination (fig. S7C). SARS-CoV-2 recovered individuals occupied a wide range of UMAP space at baseline, highlighting the variability of infection-induced virus-specific immunity (Fig. 6A). Time since infection did not appear to fully explain the observed variability for SARS-CoV-2 recovered individuals at pre-vaccine baseline (fig. S7D). Vaccination uniformly shifted SARS-CoV-2 recovered individuals at 3 months post-vaccine to a region defined by high levels of all antigen-specific immune parameters analyzed (Fig. 6A). This region was largely unoccupied by SARS-CoV-2 naïve vaccinees, underscoring the unique potency of reactivating pre-existing immune responses. These uniquely high responses were transient, however, as SARS-CoV-2 recovered individuals at 6 months post-vaccine shifted toward the UMAP space occupied by memory timepoints in SARS-CoV-2 naïve individuals at 3 and 6 months post-vaccine.

A second question is how different antigen-specific mRNA vaccine-induced immune components interact with each other over time. Antibody responses after the first or second vaccine dose did not correlate with the magnitude of B cell memory at 6 months (Fig. 6C). However, at 3- and 6-months post-vaccination antibodies were significantly associated with contemporaneous memory B cell responses, an effect most prominent for B.1.351 neutralizing titers (Fig. 6C). Given the role of Tfh cells in generating efficient humoral immunity, we next investigated the relationship between antigen-specific T cells and humoral responses. CD4+ T cell responses, especially cTfh responses, as early as 2 weeks after the first dose of mRNA vaccine were positively correlated with antibody responses up to and including 6 months post-

vaccination (Fig. 6D and fig. S7E). This observation suggested that rapid mobilization of CD4+ T cell responses by the first mRNA vaccine dose had a lasting effect on humoral immunity. Like memory B cells, the magnitude of CD4+ T cell responses at 6 months was also correlated with antibodies at 6 months (Fig. 6D), suggesting that antibody levels may provide a useful (though incomplete) proxy for the magnitude of memory B and CD4+ T cell responses at 6 months post-vaccination. Taken together, these data identify key temporal relationships between different branches of the human immune response that are associated with long-term immune memory after mRNA vaccination.

Next, we investigated if the magnitude of peak responses after the second vaccine dose in SARS-CoV-2 naïve subjects was predictive of memory responses at 3 and 6 months. Indeed, peak antibody levels were significantly correlated with later antibody levels (fig. S7F). Memory B cell frequencies 1 week after the second dose were also correlated significantly with 3- and 6-month frequencies (fig. S7F). Like antibodies and memory B cells, peak T cell responses after the second dose were predictive of later timepoints (fig. S7F). Overall, these data suggest that the magnitude and trajectory of individual components of the immune response are patterned soon after the second vaccine dose in SARS-CoV-2 naïve individuals.

This dataset also presented an opportunity to investigate the impact of mRNA vaccination in subjects with pre-existing immunity, in this case from a prior SARS-CoV-2 infection. To investigate the dynamics of these recall responses, we examined the change in individual SARS-CoV-2-specific immune responses from pre-vaccine baseline levels. Vaccination modestly increased pre-existing memory B cell and CD4+ T cell frequencies at 1 month, with a more robust increase in antibody levels (Fig. 6E). To investigate the contribution of pre-existing immune memory to these recall antibody responses, we correlated the magnitude of pre-vaccine memory responses with the change in antibody levels after vaccination. The frequency of SARS-CoV-2-specific memory B cells was the only feature of pre-existing immunity that correlated significantly with antibody responses after vaccination (Fig. 6F), consistent with a major role for memory B cells in recall responses. As we observed that memory B cell frequencies continue to increase in the months post-vaccination, we investigated whether time since infection impacted the magnitude of the antibody recall response. Indeed, a longer interval between infection and vaccination correlated with a significantly greater neutralizing antibody recall response to D614G, with similar trends for B.1.351 neutralization and for binding antibodies to Spike and RBD (Fig. 6F). Thus, these data suggest that there may be some benefit to a longer interval between initial priming and subsequent restimulation or boost of immune responses to SARS-CoV-2.

Finally, we evaluated the decay kinetics of SARS-CoV-2-specific recall responses. Boosting of Spike- and RBD-specific memory B cell and memory CD4+ T cell responses was transient and returned to pre-vaccination baseline by 3-6 months (Fig. 6E). CD8+ T cell responses were not boosted in SARS-CoV-2 immune subjects and decayed from peak at a comparable rate to SARS-CoV-2 naïve vaccinees (Fig. 6E). The increase in anti-Spike and anti-RBD binding antibodies was also transient and returned to near baseline by 6 months post-vaccine (Fig. 6E). Only D614G and B.1.351 neutralizing antibody remained substantially above pre-vaccine baseline levels (~10-fold increase at 6 months), but these antibody levels were also declining over time. Notably, the decay rate of antibodies was similar between SARS-CoV-2 naïve and SARS-CoV-2 recovered vaccinees (Fig. 6E). Lastly, we estimated the benefit of mRNA vaccine-mediated "boosting" of pre-existing immunity in this setting by calculating, based on antibody half-lives, the time it would take for recall responses to return to pre-vaccine antibody levels. We estimated from these calculations that recall responses to mRNA vaccination will maintain antibodies above pre-vaccination levels in this cohort of mostly young individuals who recovered from mild COVID-19 for approximately 7-16 months. In addition, recall responses in this cohort remained above peak responses in SARS-CoV-2 naïve vaccinees, where clinical efficacy is well-established, for 2-3 months for Spike-binding antibodies and 6-10 months for neutralizing titers (table S3). Overall, these data suggest that boosting of infection-induced immunity with mRNA vaccination does not dramatically enhance already durable memory B cell or memory T cell responses. Rather, the benefit of vaccination in the context of pre-existing immunity may be limited to a significant but transient increase in antibody, with some of this benefit to antibody levels remaining at 6 months.

Concluding Remarks

These studies provide insight into the evolution of immunological memory following SARS-CoV-2 mRNA vaccination. Specifically, the continued increase in SARS-CoV-2-specific memory B cells between 3 and 6 months post-mRNA vaccination, even as antibody levels declined in the same individuals, suggests that prolonged germinal center reactions (*14*) continue to generate circulating memory B cells for at least several months following vaccination. A majority of these memory B cells were able to cross-bind VOCs, including B.1.1.7 (Alpha), B.1.351 (Beta), and B.1.617.2 (Delta), and clonal relationships indicated that at least some of these cross-binding memory B cells evolved via somatic hypermutation from clones that initially lacked variant binding. This evolution of variant binding may have implications for booster strategies aimed at targeting antibody responses to future variants. As demonstrated here, these memory B cells are capable of

mounting rapid recall responses, providing a new source of antibodies upon infection or booster vaccination. Furthermore, there may be differences in immunity generated by mRNA vaccination versus infection, as memory B cells 6 months post-vaccination were qualitatively superior at binding VOCs compared to memory B cells 6 months after recovering from mild COVID-19. Variant binding developed rapidly following two-dose mRNA vaccination but evolved more slowly following infection, consistent with conclusions drawn from other approaches (*17*). In addition to durable B cell memory, SARS-CoV-2-specific memory CD4+ T cells were relatively stable from 3-6 months post mRNA vaccination, and the vast majority of vaccinees maintained robust CD4+ T cell responses at 6 months. Early CD4+ T cell responses correlated with 3- and 6-month humoral responses, highlighting a role for T cell immunity in shaping the overall response to vaccination. Together, these data identify durable cellular immunity for at least 6 months after mRNA vaccination with persistence of high-quality memory B cells and strong CD4+ T cell memory in most individuals.

These data may also provide context for understanding potential discrepancies in vaccine efficacy at preventing infection versus severe disease, hospitalization, and death (*10*, *11*). Declining antibody titers over time likely reduce the potential that vaccination will completely prevent infection or provide near-sterilizing immunity. However, the durability of cellular immunity, here demonstrated for at least 6 months, may contribute to rapid recall responses that can limit initial viral replication and dissemination in the host, thereby preventing severe disease. Finally, by examining individuals with pre-existing immunity following infection, we were able to gain insights into the possible effects of booster vaccination. In this setting, boosting of pre-existing immunity from prior infection with mRNA vaccination mainly resulted in a transient benefit to antibody titers with little-to-no long-term increase in cellular immune memory. Antibody decay rates were similar in SARS-CoV-2 naïve and recovered vaccinees, suggesting that additional vaccine doses will temporarily prolong antibody-mediated protection without fundamentally altering the underlying landscape of SARS-CoV-2 immune memory. It will be important to examine whether similar dynamics exist following other types of immune boosting including a third dose of mRNA vaccine in previously vaccinated individuals or SARS-CoV-2 infections that occur after vaccination. Nevertheless, these data provide evidence for durable immune memory at 6 months after mRNA vaccination and are relevant for interpreting epidemiological data on rates of infections in vaccinated populations and the implementation of booster vaccine strategies.

Despite the overall strengths of this study, including the large sample size and integrated measurement of multiple components of the antigen-specific adaptive immune response, there are several limitations. First, the overall number of subjects, while substantial for studies with high depth of immune profiling, was still limited compared to epidemiological or phase 3 clinical trials. In particular, only 9-10 individuals with pre-existing immunity from SARS-CoV-2 infection were fully sampled through 6 months post-vaccination. Second, it is possible that the timepoints in this study do not perfectly capture the full kinetics of the response for each individual immune component. For example, it is possible that antibody levels could stabilize at timepoints beyond 6 months rather than continuing to decay at the observed rates. Additionally, the comparison of variant-specific immune memory induced by vaccination versus infection is limited to mild COVID-19 cases and does not include more severe disease. Timepoints for sampling of infection only, although broadly consistent with the vaccination studies, were also not perfectly aligned with the date of actual infection as samples were longitudinally collected following a positive serology test rather than an acutely positive PCR test in most cases. Regarding CD8+ T cell responses, our AIM assay was effective at capturing peak responses after vaccination; however, this assay may not be sensitive enough to detect very low frequency CD8+ T cells at memory timepoints. Other approaches, such as MHC tetramers, will be necessary in the future to further interrogate memory CD8+ T cell responses after vaccination. Finally, our cohort is skewed toward young healthy individuals. As such, the results described may not fully represent the durability of vaccine-induced immunity in older individuals or populations with chronic diseases and/or compromised immune systems, and future studies will be required to better quantify the immune response over time in these populations.

Methods

Clinical Recruitment and Sample Collection

61 individuals (45 SARS-CoV-2 naïve, 16 SARS-CoV-2 recovered) were consented and enrolled in the longitudinal vaccine study with approval from the University of Pennsylvania Institutional Review Board (IRB# 844642). All participants were otherwise healthy and based on self-reported health screening did not have any history of chronic health conditions. Subjects were stratified based on self-reported and laboratory evidence of a prior SARS-CoV-2 infection. All subjects received either Pfizer (BNT162b2) or Moderna (mRNA-1273) mRNA vaccines. Samples were collected at 6 timepoints: baseline, ~2 weeks post-primary immunization, day of secondary immunization, ~1 week post-secondary immunization, ~3 months post-primary immunization, and ~6 months post-primary immunization. 80-100mL of peripheral blood samples and clinical questionnaire data were collected at each study visit. A separate cohort of 26 SARS-CoV-2 convalescent individuals was used to compare vaccine-induced

immune responses to immune responses upon natural SARS-CoV-2 infection. This cohort was a subset from a sero-monitoring study previously described (*40*) that was approved by the University of Pennsylvania Institutional Review Board (IRB# 842847). Recent or active SARS-CoV-2 infections were identified based on SARS-CoV-2 RBD antibody levels and/or SARS-COV-2 PCR testing. Longitudinal samples were collected from seropositive participants up to ~200 days post seroconversion to study long-term immune responses. Full cohort and demographic information is provided in table S1. Additional healthy donor samples were collected with approval from the University of Pennsylvania Institutional Review Board (IRB# 845061)

Peripheral Blood Sample Processing

Venous blood was collected into sodium heparin and EDTA tubes by standard phlebotomy. Blood tubes were centrifuged at 3000rpm for 15 min to separate plasma. Heparin and EDTA plasma were stored at -80°C for downstream antibody analysis. Remaining whole blood was diluted 1:1 with R1 (RPMI + 1% FBS + 2mM L-Glutamine + 100 U Penicillin/Streptomycin) and layered onto SEPMATE tubes (STEMCELL Technologies) containing lymphoprep gradient (STEMCELL Technologies). SEPMATE tubes were centrifuged at 1200 g for 10 min and the PBMC fraction was collected into new tubes. PBMCs were then washed with R1 and treated with ACK lysis buffer (Thermo Fisher) for 5 min. Samples were washed again with R1, filtered with a 70μm filter, and counted using a Countess automated cell counter (Thermo Fisher). Aliquots containing 5-10x10^6 PBMCs were cryopreserved in fresh 90% FBS 10% DMSO.

Detection of SARS-CoV-2 Spike- and RBD-Specific Antibodies

Plasma samples were tested for SARS-CoV-2-specific antibody by enzyme-linked immunosorbent assay (ELISA) as described (*16*, *54*). Plasmids encoding the recombinant full-length Spike protein and the RBD were provided by F. Krammer (Mt. Sinai) and purified by nickel-nitrilotriacetic acid resin (Qiagen). ELISA plates (Immulon 4 HBX, Thermo Fisher Scientific) were coated with PBS or 2 ug/mL recombinant protein and stored overnight at 4C. The next day, plates were washed with PBS containing 0.1% Tween-20 (PBS-T) and blocked for 1 hour with PBS-T supplemented with 3% non-fat milk powder. Samples were heat-inactivated for 1 hour at 56C and diluted in PBS-T supplemented with 1% non-fat milk powder. After washing the plates with PBS-T, 50 uL diluted sample was added to each well. Plates were incubated for 2 hours and washed with PBS-T. Next, 50 uL of 1:5000 diluted goat anti-human IgG-HRP (Jackson ImmunoResearch Laboratories) or 1:1000 diluted goat anti-human IgM-HRP (SouthernBiotech) was added to each well and plates were incubated for 1 hour. Plates were washed with PBS-T before 50 uL SureBlue 3,3′,5,5′-tetramethylbenzidine substrate (KPL) was added to each well. After 5 min incubation, 25 uL of 250 mM hydrochloric acid was added to each well to stop the reaction. Plates were read with the SpectraMax 190 microplate reader (Molecular Devices) at an optical density (OD) of 450 nm. Monoclonal antibody CR3022 was included on each plate to convert OD values into relative antibody concentrations. Plasmids to express CR3022 were provided by I. Wilson (Scripps).

Detection of SARS-CoV-2 Neutralizing Antibodies

293T cells were seeded for 24 hours at 5 X 10^6 cells per 10 cm dish and were transfected using calcium phosphate with 35 μg of pCG1 SARS-CoV-2 S D614G delta18, pCG1 SARS-CoV-2 S B.1.351 delta18 or pCG1 SARS-CoV-2 S B.1.617.2 delta18 expression plasmid encoding a codon optimized SARS-CoV-2 S gene with an 18-residue truncation in the cytoplasmic tail (kindly provided by Stefan Pohlmann). Mutations in pseudovirus constructs are indicated: D614G (WT) = D614G; B.1.351 = L18F, D80A, D215G, R246I, K417N, E484K, N501Y, D614G, A701V; B.1.617.2 = T19R, G142D, del156-157, R158G, L452R, T478K, D614G, P681R, D950N. 12 hours post transfection, cells were fed with fresh media containing 1mM sodium butyrate to increase expression of the transfected DNA. 24 hours after transfection, the SARS-CoV-2 Spike expressing cells were infected for 2 hours with VSV-G pseudotyped VSV∆G-RFP at an MOI of ~1. Virus containing media was removed and the cells were re-fed with media without serum. Media containing the VSV∆G-RFP SARS-CoV-2 pseudotypes was harvested 28-30 hours after infection, clarified by centrifugation twice at 6000 g, then aliquoted and stored at -80°C until used for antibody neutralization analysis. All sera were heat-inactivated for 30 min at 55°C prior to use in the neutralization assay. Vero E6 cells stably expressing TMPRSS2 were seeded in 100 μl at 2.5x10^4 cells/well in a 96 well collagen coated plate. The next day, 2-fold serially diluted serum samples were mixed with VSV∆G-RFP SARS-CoV-2 pseudotype virus (100-300 focus forming units/well) and incubated for 1 hour at 37°C. 1E9F9, a mouse anti-VSV Indiana G, was also included in this mixture at a concentration of 600 ng/ml (Absolute Antibody, Ab01402-2.0) to neutralize any potential VSV-G carryover virus. The serum-virus mixture was then used to replace the media on VeroE6 TMPRSS2 cells. 22 hours post-infection, the cells were washed and fixed with 4% paraformaldehyde before visualization on an S6 FluoroSpot Analyzer (CTL, Shaker Heights OH). Individual infected foci were enumerated and the values were compared to control wells without antibody. The focus reduction neutralization titer 50% ($FRNT_{50}$) was measured as the greatest serum dilution at which focus count was reduced by at least 50% relative to control cells that were infected with pseudotype virus in

the absence of human serum. $FRNT_{50}$ titers for each sample were measured in at least two technical replicates and were reported for each sample as the geometric mean of the technical replicates.

Detection and Phenotyping of SARS-CoV-2-Specific Memory B Cells

Antigen-specific B cells were detected using biotinylated proteins in combination with different streptavidin (SA)-fluorophore conjugates as described (*16*). All reagents are listed in table S4. Biotinylated proteins were multimerized with fluorescently labeled SA for 1 hour at 4C. Full-length Spike protein was mixed with SA-BV421 at a 10:1 mass ratio (200ng Spike with 20ng SA; ~4:1 molar ratio). Spike RBD was mixed with SA-APC at a 2:1 mass ratio (25ng RBD with 12.5ng SA; ~4:1 molar ratio). Biotinylated influenza HA pools were mixed with SA-PE at a 6.25:1 mass ratio (100ng HA pool with 16ng SA; ~6:1 molar ratio). Influenza HA antigens corresponding with the 2019 trivalent vaccine (A/Brisbane/02/2018/H1N1, B/Colorado/06/2017) were chosen as a historical antigen and were biotinylated using an EZ-Link Micro NHS-PEG4 Biotinylation Kit (Thermo Fisher) according to the manufacturer's instructions. Excess biotin was subsequently removed from HA antigens using Zebra Spin Desalting Columns 7K MWCO (Thermo Fisher) and protein was quantified with a Pierce BCA Assay (Thermo Fisher). SA-BV711 was used as a decoy probe without biotinylated protein to gate out cells that non-specifically bind streptavidin. All experimental steps were performed in a 50/50 mixture of PBS + 2% FBS and Brilliant Buffer (BD Bioscience). Antigen probes for Spike, RBD, and HA were prepared individually and mixed together after multimerization with 5uM free D-biotin (Avidity LLC) to minimize potential cross-reactivity between probes. For staining, $5x10^6$ cryopreserved PBMC samples were prepared in a 96-well U-bottom plate. Cells were first stained with Fc block (Biolegend, 1:200) and Ghost 510 Viability Dye for 15 min at 4C. Cells were then washed and stained with 50uL antigen probe master mix containing 200ng Spike-BV421, 25ng RBD-APC, 100ng HA-PE, and 20ng SA-BV711 decoy for 1 hour at 4C. Following incubation with antigen probe, cells were washed again and stained with anti-CD3, anti-CD19, anti-CD20, anti-CD27, anti-CD38, anti-CD71, anti-IgD, anti-IgM, anti-IgG, and anti-IgA for 30 min at 4C. After surface stain, cells were washed and fixed in 1% PFA overnight at 4C. Antigen-specific gates for B cell probe assays were set based on healthy donors stained without antigen probes (similar to an FMO control) and were kept the same for all experimental runs.

Detection of Variant RBD, NTD, and S2-Specific Memory B Cells

Variant RBD, NTD, and S2-specific memory B cells were detected using a similar approach as described above. SARS-CoV-2 nucleocapsid was used as a vaccine-irrelevant antigen control. All reagents are listed in table S4. Probes were multimerized for 1.5 hours at the following ratios (all ~4:1 molar ratios calculated relative to the streptavidin-only component irrespective of fluorophore): 200ng full-length Spike protein was mixed with 20ng SA-BV421, 30ng N-terminal domain was mixed with 12ng SA-BV786, 25ng wild-type RBD was mixed with 12.5ng SA-BB515, 25ng B.1.1.7 RBD was mixed with 12.5ng SA-BV711, 25ng B.1.351 RBD was mixed with 12.5ng SA-PE, 25ng B.1.617.2 was mixed with 12.5ng SA-APC, 50ng S2 was mixed with 12ng SA-BUV737, 50ng nucleocapsid was mixed with 14ng SA-BV605. 12.5ng SA-BUV615 was used as a decoy probe. All antigen probes were multimerized separately and mixed together with 5uM free D-biotin. Prior to staining, total B cells were enriched from $20x10^6$ cryopreserved PBMC samples by negative selection using an EasySep human B cell isolation kit (STEMCELL, #17954). B cells were then prepared in a 96-well U-bottom plate and stained with Fc block and Ghost 510 Viability Dye as described above. Cells were washed and stained with 50uL antigen probe master mix for 1 hour at 4C. After probe staining, cells were washed again and stained with anti-CD3, anti-CD19, anti-CD27, anti-CD38, anti-IgD, and anti-IgG for 30 min at 4C. After surface stain, cells were washed and fixed in 1X Stabilizing Fixative (BD Biosciences) overnight at 4C.

For sorting, pre-enriched B cells were stained with Fc block and Ghost 510 Viability Dye, followed by full-length Spike, WT RBD, and B.1.351 RBD probes as described above. Cells were then stained for surface markers with anti-CD19, anti-CD20, anti-CD27, and anti-CD38, and anti-IgD. After surface stain, cells were washed and resuspended in PBS + 2% FBS for acquisition.

In Vitro Differentiation of Memory B Cells to Antibody Secreting Cells

Memory B cells from bulk PBMC samples were differentiated into antibody secreting cells as described (*39*). Briefly, $1x10^6$ cryopreserved PBMCs were seeded in 1mL of complete RPMI media (RPMI + 10% FBS + 1% Pen/Strep) in 24-well plates. PBMCs were then stimulated with 1000U/mL recombinant human IL-2 and 2.5ug/mL R848 for 10 days. Supernatants were collected at the indicated timepoints. anti-Spike IgG was quantified using a Human SARS-CoV-2 Spike (Trimer) IgG ELISA Kit (Invitrogen) according to the manufacturer's instructions. RBD-ACE2 binding inhibition was measured using a SARS-CoV-2 Neutralizing Ab ELISA Kit (Invitrogen). For anti-Spike IgG experiments, culture supernatants were tested at 1:100 and 1:1000 dilutions. For RBD inhibition experiments, culture supernatants were tested without dilution and at a 1:2 dilution. Pseudovirus neutralization titers were also measured in culture supernatants starting at a 1:2

dilution as described above.

Detection of SARS-CoV-2-Specific T Cells

SARS-CoV-2-specific T cells were detected using an activation induced marker assay. All reagents are listed in table S5. PBMCs were thawed by warming frozen cryovials in a 37°C water bath and resuspending cells in 10mL of RPMI supplemented with 10% FBS, 2mM L-Glutamine, 100 U/mL Penicillin, and 100 ug/mL Streptomycin (R10). Cells were washed once in R10, counted using a Countess automated cell counter (Thermo Fisher), and resuspended in fresh R10 to a density of 5x10^6 cells/mL. For each condition, duplicate wells containing 1x10^6 cells in 200uL were plated in 96-well round-bottom plates and rested overnight in a humidified incubator at 37°C, 5% CO2. After 16 hours, CD40 blocking antibody (0.5ug/mL final concentration) was added to cultures for 15 min prior to stimulation. Cells were then stimulated for 24 hours with costimulation (anti-human CD28/CD49d, BD Biosciences) and peptide megapools (CD4-S for all CD4+ T cell analyses, CD8-E for all CD8+ T cell analyses) at a final concentration of 1 ug/mL. Peptide megapools were prepared as previously described (*51*, *52*). Matched unstimulated samples for each donor at each timepoint were treated with costimulation alone. 20 hours post-stimulation, antibodies targeting CXCR3, CCR7, CD40L, CD107a, CXCR5, and CCR6 were added to the culture along with monensin (GolgiStop, BD Biosciences) for a 4-hour stain at 37°C. After 4 hours, duplicate wells were pooled and cells were washed in PBS supplemented with 2% FBS (FACS buffer). Cells were stained for 10 min at room temperature with Ghost Dye Violet 510 and Fc receptor blocking solution (Human TruStain FcX, BioLegend) and washed once in FACS buffer. Surface staining for 30 min at room temperature was then performed with antibodies directed against CD4, CD8, CD45RA, CD27, CD3, CD69, CD40L, CD200, OX40, and 41BB in FACS buffer. Cells were washed once in FACS buffer, fixed and permeabilized for 30 min at room temperature (eBioscience Foxp3 / Transcription Factor Fixation/Permeabilization Concentrate and Diluent), and washed once in 1X Permeabilization Buffer prior to staining for intracellular IFN-g overnight at 4°C. Cells were then washed again and resuspended in 1% paraformaldehyde in PBS prior to data acquisition.

All data from AIM expression assays were background-subtracted using paired unstimulated control samples. For memory T cell and helper T cell subsets, the AIM+ background frequency of non-naïve T cells was subtracted independently for each subset. AIM+ cells were identified from non-naïve T cell populations. AIM+ CD4+ T cells were defined by co-expression of CD200 and CD40L. AIM+ CD8+ T cells were defined by a Boolean analysis identifying cells expressing at least four of five markers: CD200, CD40L, 41BB, CD107a, and intracellular IFN-g.

Flow Cytometry and Cell Sorting

Samples were acquired on a BD Symphony A5 instrument. Standardized SPHERO rainbow beads (Spherotech) were used to track and adjust photomultiplier tubes over time. UltraComp eBeads (Thermo Fisher) were used for compensation. Up to 5x10^6 cells were acquired per sample. Data were analyzed using FlowJo v10 (BD Bioscience). For Boolean analysis of variant cross-binding, data were imported into SPICE 6 (NIH Vaccine Research Center (*55*)). Cell sorting was performed on a BD FACS Aria II instrument in low pressure mode using a 70um nozzle. Cells were sorted into DNA LoBind Eppendorf tubes containing cell lysis buffer (Qiagen).

B Cell Receptor Sequencing

Library Preparation

DNA was extracted from sorted cells using a Gentra Puregene Cell kit (Qiagen, catalog no. 158767). Immunoglobulin heavy-chain family–specific PCRs were performed on genomic DNA samples using primers in FR1 and JH as described previously (*47*, *56*). Two biological replicates were run on all samples. Sequencing was performed in the Human Immunology Core Facility at the University of Pennsylvania using an Illumina 2× 300-bp paired-end kit (Illumina MiSeq Reagent Kit v3, 600-cycle, Illumina MS-102-3003).

IGH Sequence Analysis

Reads from an Illumina MiSeq were filtered, annotated, and grouped into clones as described previously (*16*, *57*). Briefly, pRESTO v0.6.0 (*58*) was used to align paired end reads, remove short and low-quality reads, and mask low-quality bases with *N*s to avoid skewing SHM and lineage analyses. Sequences which passed this process were aligned and annotated with IgBLAST v1.17.0 (*59*). The annotated sequences were then imported into ImmuneDB v0.29.10 (*60*, *61*) for clonal inference, lineage construction, and downstream processing. For clonal inference, sequences with the same IGHV gene, IGHJ gene, and CDR3 length from each donor were hierarchically clustered. Sequences with 85% or higher similarity in their CDR3 amino-acid sequence were subsequently grouped into clones. Clones with productive rearrangements and >= 2 copies were filtered for downstream analysis.

Lineage Construction & Visualization

For each clone, a lineage was constructed with ImmuneDB as described in (*61*). ete3 (*62*) was used to visualize the lineages where each node represents a unique sequence, the size of a node represents its relative copy number fraction in the clone, and the integer next to each node represents the number of mutations from the preceding vertical node.

Overlapping Clone SHM Analysis

Clones were filtered based on size using a copy number filter

such that clones which had a copy number less than 50% of the mean copy number frequency (50% mcf) within the subject were excluded. From this population, only clones that appeared in both WT RBD and cross-binder (RBD++) samples were included. The SHM of each clone was averaged across each unique sequence, weighted by the copies of each sequence, and visualized as categorical variables (pie chart) and as frequencies (boxplots).

Data Availability

Raw sequencing data for all donors and subsets is available on SRA under BioProject PRJNA752617. Processed AIRR-seq data will be made available on the AIRR Data Commons via the iReceptor portal (*63*).

Estimating Decay Rates

To understand and compare the rate of loss of immune responses after vaccination, we tested different statistical models of decay against the data. We first tested if there was significant decay (i.e., was the decay rate significantly different from zero). We then tested if there was evidence for a slowing of decay with time (using a two-phase model). This is a heuristic approach to understanding decay and does not imply a mechanism or that the underlying immune dynamics may be more complex. The decay rate post-second dose of vaccine was estimated using a censored mixed effect regression framework. Briefly, the dependency of variables of interest on days post vaccine can be modeled by using either one constant decay slope or a decay slope that changes with time (assume a two- phase decay with a fixed break point at T_0). The model of the immune response y for participant i at time t_{ij} can be written as below:

$y_{ij} = \beta_0 + b_{0i} + \beta_1 t_{ij} + b_{1i} t_{ij}$ – for a model with a single slope; and

$y_{ij} = \beta_0 + b_{0i} + \beta_1 t_{ij} + b_{1i} t_{ij} + \beta_2 s_{ij}$ – for a model with two different slopes, in which:

$$s_{ij} = \begin{cases} 0, t_{ij} < T_0 \\ t_{ij} - T_0, t_{ij} \geq T_0 \end{cases}$$

The parameter β_0 is a constant (global intercept), and b_{0i} is a patient-specific adjustment (random effect) to the global intercept. The slope parameter β_1 is a fixed effect to capture the average decay rate for all individuals before T_0; and b_{1i} is a patient-specific random effect of the decay rate. To fit the model with a two-phase decay slope (with break point at time T_0), an extra parameter β_2 (with a subject-specific random effect b_{2i}) was added to represent the difference between the two slopes. Throughout the manuscript, we chose the median of the timepoints post-second dose of vaccine as the break point in decay rate (i.e., T_0=day 89).

To account for values less than the detection threshold in the assay, a censored mixed-effect regression method was used to estimate the parameters in the model. Values less than 10 were censored for the neutralization data. For T cell measurements, this detection threshold varies (see supplemental information – determining the limit of detection for details on how this variable limit of detection was captured). The linear models above were fitted with censoring of values below the limit of detection using lmec library in R (*64*) (with the maximum likelihood algorithm option to fit for the fixed effects). We used a likelihood ratio test to determine if the response variables where better fit with either the single or two-phase decay models (by testing whether β_2 = 0), and to test whether the decay rates were different between SARS-CoV-2 naïve and recovered subjects (this test compares the likelihood value of the nested models and the difference in the number of parameters). These analyses were carried out in R version 4.0.4.

Determining the Limit of Detection for Estimating Decay Rates

For each individual and at each time point (i.e., each sample) the limit of detection in assays of T cell stimulation varied. This is because the background level is determined by running paired assessment of cells from a given sample in (SARS-CoV-2 peptide) stimulated and unstimulated cultures. The quantify of interest (of which we wish to measure the decay rate) is the difference in the fraction of T cells activated in the stimulated and unstimulated cultures. The variable limit of detection (LOD) for each sample must be considered when determining the decay rate for T cell responses. To determine if the fraction of activated cells in a stimulated sample was significantly higher than the fraction of activated cells in the corresponding unstimulated sample (i.e., if the sample was above the limit of detection) we used a one-sided two proportion Z test. Formally, we let the proportion of unstimulated and stimulated responses (over total non-naïve cells) be denoted by $U_{i,j}$ and $S_{i,j}$ for patient i at time j, respectively. It follows that we are interested in estimating the decay rate of the quantity $\Delta_{i,j} = S_{i,j} - U_{i,j}$. A one-sided two proportion Z test was used to determine if $S_{i,j} > U_{i,j}$. Briefly, for each patient i at time j, the following quantity was calculated:

$$Z_{i,j} = \frac{\Delta_{i,j}}{\sqrt{p(1-p)\left(\frac{1}{n_{s_{i,j}}} + \frac{1}{n_{u_{i,j}}}\right)}}$$

With:

$$\Delta_{i,j} = S_{i,j} - U_{i,j},$$

$$p = \frac{s_{i,j} \times n_{s_{i,j}} + u_{i,j} \times n_{u_{i,j}}}{n_{s_{i,j}} + n_{u_{i,j}}},$$

$n_{s_{i,j}}$ = total non-naïve cells in stimulated group for subject i at time j,

$n_{u_{i,j}}$ = total non-naïve cells in unstimulated group for subject i at time j.

For each subject, we calculated the minimum difference needed to achieve significance by solving the above equation for $\Delta_{i,j}$ (assuming p is constant) at the $Z_{critical}$ level (i.e., with α=0.05, $Z_{critical}$=1.645 for a one-sided test). This minimum difference can be written as:

$$\Delta_{MIN_{i,j}} = 1.645 \times \sqrt{p(1-p)\left(\frac{1}{n_{s_{i,j}}} + \frac{1}{n_{u_{i,j}}}\right)}$$

We censored subject i if the difference is not statistically significant (i.e., $Z_{i,j}$<1.645, with α=0.05). The detection limit for subject i was calculated by taking the maximum value of $\Delta_{MIN_{i,j}}$ across all timepoint for that subject. The values $\Delta_{i,j}$ were normalized by the maximum $\Delta_{MIN_{i,j}}$ for each subject, hence the limit of detection was set to zero, and the lmec regression models applied to the normalized data in order to determine the decay rates of T cell responses.

High Dimensional Analysis and Statistics

All data were analyzed using custom scripts in R and visualized using RStudio. Pairwise correlations between variables were calculated and visualized as a correlogram using corrplot with FDR correction as described previously (*65*). For heatmaps, data were visualized with pheatmap. For construction of UMAPs, 12 antigen-specific immune features were selected: anti-Spike IgG, anti-RBD IgG, D614G FRNT50, B.1.351 FRNT50, Spike+ memory B, RBD+ memory B, % IgG+ of Spike+ memory B, % IgG+ of RBD+ memory B, AIM+ CD4 T, AIM+ CD4 Tfh, AIM+ CD4 Th1, and AIM+ CD8 T. Antibody and cell frequency data were log10 transformed and scaled by column (z-score normalization) prior to generating UMAP coordinates. Statistical tests are indicated in the corresponding figure legends. All tests were performed two-sided with a nominal significance threshold of $p < 0.05$. Benjamini-Hochberg (BH) correction was performed in all cases of multiple comparisons. Unpaired tests were used for comparisons between timepoints unless otherwise indicated as some participants were missing samples from individual timepoints. * indicates $p < 0.05$, ** indicates $p < 0.01$, *** indicates $p < 0.001$, **** indicates $p < 0.0001$. Source code and data files are available upon request from the authors.

REFERENCES AND NOTES

1. T. Carvalho, F. Krammer, A. Iwasaki, The first 12 months of COVID-19: A timeline of immunological insights. *Nat. Rev. Immunol.* **21**, 245–256 (2021). doi:10.1038/s41577-021-00522-1 Medline
2. F. P. Polack, S. J. Thomas, N. Kitchin, J. Absalon, A. Gurtman, S. Lockhart, J. L. Perez, G. Pérez Marc, E. D. Moreira, C. Zerbini, R. Bailey, K. A. Swanson, S. Roychoudhury, K. Koury, P. Li, W. V. Kalina, D. Cooper, R. W. Frenck, L. L. Hammitt, Ö. Türeci, H. Nell, A. Schaefer, S. Ünal, D. B. Tresnan, S. Mather, P. R. Dormitzer, U. Şahin, K. U. Jansen, W. C. Gruber, Safety and Efficacy of the BNT162b2 mRNA Covid-19 Vaccine. *N. Engl. J. Med.* **383**, 2603–2615 (2020). doi:10.1056/NEJMoa2034577
3. L. R. Baden, H. M. El Sahly, B. Essink, K. Kotloff, S. Frey, R. Novak, D. Diemert, S. A. Spector, N. Rouphael, C. B. Creech, J. McGettigan, S. Khetan, N. Segall, J. Solis, A. Brosz, C. Fierro, H. Schwartz, K. Neuzil, L. Corey, P. Gilbert, H. Janes, D. Follmann, M. Marovich, J. Mascola, L. Polakowski, J. Ledgerwood, B. S. Graham, H. Bennett, R. Pajon, C. Knightly, B. Leav, W. Deng, H. Zhou, S. Han, M. Ivarsson, J. Miller, T. Zaks, Efficacy and Safety of the mRNA-1273 SARS-CoV-2 Vaccine. *N. Engl. J. Med.* **384**, 403–416 (2021). doi:10.1056/NEJMoa2035389
4. D. S. Khoury, D. Cromer, A. Reynaldi, T. E. Schlub, A. K. Wheatley, J. A. Juno, K. Subbarao, S. J. Kent, J. A. Triccas, M. P. Davenport, Neutralizing antibody levels are highly predictive of immune protection from symptomatic SARS-CoV-2 infection. *Nat. Med.* **27**, 1205–1211 (2021). doi:10.1038/s41591-021-01377-8 Medline
5. D. Cromer, M. Steain, A. Reynaldi, T. E. Schlub, A. K. Wheatley, J. A. Juno, S. J. Kent, J. A. Triccas, D. S. Khoury, M. P. Davenport, SARS-CoV-2 variants: levels of neutralisation required for protective immunity. medRxiv 2021.08.11.21261876 [Preprint] (2021). https://doi.org/10.1101/2021.08.11.21261876.
6. P. B. Gilbert, D. C. Montefiori, A. McDermott, Y. Fong, D. Benkeser, W. Deng, H. Zhou, C. R. Houchens, K. Martins, L. Jayashankar, F. Castellino, B. Flach, B. C. Lin, S. O'Connell, C. McDanal, A. Eaton, M. Sarzotti-Kelsoe, Y. Lu, C. Yu, B. Borate, L. W. P. van der Laan, N. Hejazi, C. Huynh, J. Miller, H. M. El Sahly, L. R. Baden, M. Baron, L. De La Cruz, C. Gay, S. Kalams, C. F. Kelley, M. Kutner, M. P. Andrasik, J. G. Kublin, L. Corey, K. M. Neuzil, L. N. Carpp, R. Pajon, D. Follmann, R. O. Donis, R. A. Koup, Immune Correlates Analysis of the mRNA-1273 COVID-19 Vaccine Efficacy Trial. medRxiv 2021.08.09.21261290 [Preprint] (2021). https://doi.org/10.1101/2021.08.09.21261290.
7. N. Doria-Rose, M. S. Suthar, M. Makowski, S. O'Connell, A. B. McDermott, B. Flach, J. E. Ledgerwood, J. R. Mascola, B. S. Graham, B. C. Lin, S. O'Dell, S. D. Schmidt, A. T. Widge, V.-V. Edara, E. J. Anderson, L. Lai, K. Floyd, N. G. Rouphael, V. Zarnitsyna, P. C. Roberts, M. Makhene, W. Buchanan, C. J. Luke, J. H. Beigel, L. A. Jackson, K. M. Neuzil, H. Bennett, B. Leav, J. Albert, P. Kunwar, mRNA-1273 Study Group, Antibody Persistence through 6 Months after the Second Dose of mRNA-1273 Vaccine for Covid-19. *N. Engl. J. Med.* **384**, 2259–2261 (2021). doi:10.1056/NEJMc2103916 Medline
8. M. Bergwerk, T. Gonen, Y. Lustig, S. Amit, M. Lipsitch, C. Cohen, M. Mandelboim, E. G. Levin, C. Rubin, V. Indenbaum, I. Tal, M. Zavitan, N. Zuckerman, A. Bar-Chaim, Y. Kreiss, G. Regev-Yochay, Covid-19 Breakthrough Infections in Vaccinated Health Care Workers. *N. Engl. J. Med.* 10.1056/NEJMoa2109072 (2021). doi:10.1056/NEJMoa2109072
9. A. Israel, E. Merzon, A. A. Schäffer, Y. Shenhar, I. Green, A. Golan-Cohen, E. Ruppin, E. Magen, S. Vinker, Elapsed time since BNT162b2 vaccine and risk of SARS-CoV-2 infection in a large cohort. medRxiv 2021.08.03.21261496 [Preprint] (2021). https://doi.org/10.1101/2021.08.03.21261496.
10. S. Y. Tartof, J. M. Slezak, H. Fischer, V. Hong, B. K. Ackerson, O. N. Ranasinghe, T. B. Frankland, O. A. Ogun, J. M. Zamparo, S. Gray, S. R. Valluri, K. Pan, F. J. Angulo, L. Jodar, J. M. McLaughlin, Effectiveness of mRNA BNT162b2 COVID-19 vaccine up to 6 months in a large integrated health system in the USA: A retrospective cohort study. *Lancet* 10.1016/S0140-6736(21)02183-8 (2021). doi:10.1016/S0140-6736(21)02183-8 Medline
11. J. B. Griffin, M. Haddix, P. Danza, R. Fisher, T. H. Koo, E. Traub, P. Gounder, C. Jarashow, S. Balter, SARS-CoV-2 Infections and Hospitalizations Among Persons Aged ≥16 Years, by Vaccination Status — Los Angeles County, California, May 1–July 25, 2021. *MMWR Morb. Mortal. Wkly. Rep.* **70**, 1170–1176 (2021). doi:10.15585/mmwr.mm7034e5
12. S. J. Thomas, E. D. Moreira Jr., N. Kitchin, J. Absalon, A. Gurtman, S. Lockhart, J. L. Perez, G. Pérez Marc, F. P. Polack, C. Zerbini, R. Bailey, K. A. Swanson, X. Xu, S. Roychoudhury, K. Koury, S. Bouguermouh, W. V. Kalina, D. Cooper, R. W. Frenck Jr., L. L. Hammitt, Ö. Türeci, H. Nell, A. Schaefer, S. Ünal, Q. Yang, P. Liberator, D. B. Tresnan, S. Mather, P. R. Dormitzer, U. Şahin, W. C. Gruber, K. U. Jansen, C4591001 Clinical Trial Group, Six Month Safety and Efficacy of the BNT162b2 mRNA COVID-19 Vaccine. medRxiv 2021.07.28.21261159 [Preprint] (2021). https://doi.org/10.1101/2021.07.28.21261159.
13. P. S. Arunachalam, M. K. D. Scott, T. Hagan, C. Li, Y. Feng, F. Wimmers, L. Grigoryan, M. Trisal, V. V. Edara, L. Lai, S. E. Chang, A. Feng, S. Dhingra, M. Shah,

A. S. Lee, S. Chinthrajah, S. B. Sindher, V. Mallajosyula, F. Gao, N. Sigal, S. Kowli, S. Gupta, K. Pellegrini, G. Tharp, S. Maysel-Auslender, S. Hamilton, H. Aoued, K. Hrusovsky, M. Roskey, S. E. Bosinger, H. T. Maecker, S. D. Boyd, M. M. Davis, P. J. Utz, M. S. Suthar, P. Khatri, K. C. Nadeau, B. Pulendran, Systems vaccinology of the BNT162b2 mRNA vaccine in humans. *Nature* **596**, 410–416 (2021). doi:10.1038/s41586-021-03791-x Medline

14. J. S. Turner, J. A. O'Halloran, E. Kalaidina, W. Kim, A. J. Schmitz, J. Q. Zhou, T. Lei, M. Thapa, R. E. Chen, J. B. Case, F. Amanat, A. M. Rauseo, A. Haile, X. Xie, M. K. Klebert, T. Suessen, W. D. Middleton, P.-Y. Shi, F. Krammer, S. A. Teefey, M. S. Diamond, R. M. Presti, A. H. Ellebedy, SARS-CoV-2 mRNA vaccines induce persistent human germinal centre responses. *Nature* **596**, 109–113 (2021). doi:10.1038/s41586-021-03738-2 Medline
15. K. Lederer, K. Parvathaneni, M. M. Painter, E. Bettini, D. Agarwal, K. A. Lundgreen, M. Weirick, R. R. Goel, X. Xu, E. M. Drapeau, S. Gouma, A. R. Greenplate, C. Le Coz, N. Romberg, L. Jones, M. Rosen, B. Besharatian, M. Kaminiski, D. Weiskopf, A. Sette, S. E. Hensley, P. Bates, E. J. Wherry, A. Naji, V. Bhoj, M. Locci, Germinal center responses to SARS-CoV-2 mRNA vaccines in healthy and immunocompromised individuals. medRxiv 2021.09.16.21263686 [Preprint] (2021). https://doi.org/10.1101/2021.09.16.21263686.
16. R. R. Goel, S. A. Apostolidis, M. M. Painter, D. Mathew, A. Pattekar, O. Kuthuru, S. Gouma, P. Hicks, W. Meng, A. M. Rosenfeld, S. Dysinger, K. A. Lundgreen, L. Kuri-Cervantes, S. Adamski, A. Hicks, S. Korte, D. A. Oldridge, A. E. Baxter, J. R. Giles, M. E. Weirick, C. M. McAllister, J. Dougherty, S. Long, K. D'Andrea, J. T. Hamilton, M. R. Betts, E. T. Luning Prak, P. Bates, S. E. Hensley, A. R. Greenplate, E. J. Wherry, Distinct antibody and memory B cell responses in SARS-CoV-2 naïve and recovered individuals after mRNA vaccination. *Sci. Immunol.* **6**, eabi6950 (2021). doi:10.1126/sciimmunol.abi6950 Medline
17. A. Cho, F. Muecksch, D. Schaefer-Babajew, Z. Wang, S. Finkin, C. Gaebler, V. Ramos, M. Cipolla, P. Mendoza, M. Agudelo, E. Bednarski, J. DaSilva, I. Shimeliovich, J. Dizon, M. Daga, K. Millard, M. Turroja, F. Schmidt, F. Zhang, T. B. Tanfous, M. Jankovic, T. Y. Oliveria, A. Gazumyan, M. Caskey, P. D. Bieniasz, T. Hatziioannou, M. C. Nussenzweig, Anti- SARS-CoV-2 Receptor Binding Domain Antibody Evolution after mRNA Vaccination. bioRxiv 2021.07.29.454333 [Preprint] (2021). https://doi.org/10.1101/2021.07.29.454333.
18. A. Mazzoni, N. Di Lauria, L. Maggi, L. Salvati, A. Vanni, M. Capone, G. Lamacchia, E. Mantengoli, M. Spinicci, L. Zammarchi, S. T. Kiros, A. Rocca, F. Lagi, M. G. Colao, P. Parronchi, C. Scaletti, L. Turco, F. Liotta, G. M. Rossolini, L. Cosmi, A. Bartoloni, F. Annunziato, COVID-19 Research Group, First-dose mRNA vaccination is sufficient to reactivate immunological memory to SARS-CoV-2 in subjects who have recovered from COVID-19. *J. Clin. Invest.* **131**, e149150 (2021). doi:10.1172/JCI149150 Medline
19. M. M. Painter, D. Mathew, R. R. Goel, S. A. Apostolidis, A. Pattekar, O. Kuthuru, A. E. Baxter, R. S. Herati, D. A. Oldridge, S. Gouma, P. Hicks, S. Dysinger, K. A. Lundgreen, L. Kuri-Cervantes, S. Adamski, A. Hicks, S. Korte, J. R. Giles, M. E. Weirick, C. M. McAllister, J. Dougherty, S. Long, K. D'Andrea, J. T. Hamilton, M. R. Betts, P. Bates, S. E. Hensley, A. Grifoni, D. Weiskopf, A. Sette, A. R. Greenplate, E. J. Wherry, Rapid induction of antigen-specific $CD4^+$ T cells is associated with coordinated humoral and cellular immunity to SARS-CoV-2 mRNA vaccination. *Immunity* **54**, 2133–2142.e3 (2021). doi:10.1016/j.immuni.2021.08.001 Medline
20. V. Oberhardt, H. Luxenburger, J. Kemming, I. Schulien, K. Ciminski, S. Giese, B. Csernalabics, J. Lang-Meli, I. Janowska, J. Staniek, K. Wild, K. Basho, M. S. Marinescu, J. Fuchs, F. Topfstedt, A. Janda, O. Sogukpinar, H. Hilger, K. Stete, F. Emmerich, B. Bengsch, C. F. Waller, S. Rieg, Sagar, T. Boettler, K. Zoldan, G. Kochs, M. Schwemmle, M. Rizzi, R. Thimme, C. Neumann-Haefelin, M. Hofmann, Rapid and stable mobilization of $CD8^+$ T cells by SARS-CoV-2 mRNA vaccine. *Nature* **597**, 268–273 (2021). doi:10.1038/s41586-021-03841-4 Medline
21. A. Tarke, J. Sidney, N. Methot, E. D. Yu, Y. Zhang, J. M. Dan, B. Goodwin, P. Rubiro, A. Sutherland, E. Wang, A. Frazier, S. I. Ramirez, S. A. Rawlings, D. M. Smith, R. da Silva Antunes, B. Peters, R. H. Scheuermann, D. Weiskopf, S. Crotty, A. Grifoni, A. Sette, Impact of SARS-CoV-2 variants on the total $CD4^+$ and $CD8^+$ T cell reactivity in infected or vaccinated individuals. *Cell Rep. Med.* **2**, 100355 (2021). doi:10.1016/j.xcrm.2021.100355
22. J. Mateus, J. M. Dan, Z. Zhang, C. R. Moderbacher, M. Lammers, B. Goodwin, A. Sette, S. Crotty, D. Weiskopf, Low dose mRNA-1273 COVID-19 vaccine generates durable T cell memory and antibodies enhanced by pre-existing crossreactive T cell memory. medRxiv 2021.06.30.21259787 [Preprint] (2021). https://doi.org/10.1101/2021.06.30.21259787.
23. D. Cromer, J. A. Juno, D. Khoury, A. Reynaldi, A. K. Wheatley, S. J. Kent, M. P. Davenport, Prospects for durable immune control of SARS-CoV-2 and prevention of reinfection. *Nat. Rev. Immunol.* **21**, 395–404 (2021). doi:10.1038/s41577-021-00550-x Medline
24. M. Akkaya, K. Kwak, S. K. Pierce, B cell memory: Building two walls of protection against pathogens. *Nat. Rev. Immunol.* **20**, 229–238 (2020). doi:10.1038/s41577-019-0244-2 Medline
25. D. L. Farber, N. A. Yudanin, N. P. Restifo, Human memory T cells: Generation, compartmentalization and homeostasis. *Nat. Rev. Immunol.* **14**, 24–35 (2013). doi:10.1038/nri3567 Medline
26. M. C. Shamier, A. Tostmann, S. Bogers, J. de Wilde, J. IJpelaar, W. A. van der Kleij, H. de Jager, B. Haagmans, R. Molenkamp, B. B. O. Munnink, C. van Rossum, J. Rahamat-Langendoen, N. van der Geest, C. P. Bleeker-Rovers, H. Wertheim, M. P. G. Koopmans, C. H. GeurtsvanKessel, Virological characteristics of SARS-CoV-2 vaccine breakthrough infections in health care workers. medRxiv 2021.08.20.21262158 [Preprint] (2021). https://doi.org/10.1101/2021.08.20.21262158.
27. R. Ke, P. P. Martinez, R. L. Smith, L. L. Gibson, C. J. Achenbach, S. McFall, C. Qi, J. Jacob, E. Dembele, C. Bundy, L. M. Simons, E. A. Ozer, J. F. Hultquist, R. Lorenzo-Redondo, A. K. Opdycke, C. Hawkins, R. L. Murphy, A. Mirza, M. Conte, N. Gallagher, C. H. Luo, J. Jarrett, A. Conte, R. Zhou, M. Farjo, G. Rendon, C. J. Fields, L. Wang, R. Fredrickson, M. E. Baughman, K. K. Chiu, H. Choi, K. R. Scardina, A. N. Owens, J. Broach, B. Barton, P. Lazar, M. L. Robinson, H. H. Mostafa, Y. C. Manabe, A. Pekosz, D. D. McManus, C. B. Brooke, Longitudinal analysis of SARS-CoV-2 vaccine breakthrough infections reveal limited infectious virus shedding and restricted tissue distribution. medRxiv 2021.08.30.21262701 [Preprint] (2021). https://doi.org/10.1101/2021.08.30.21262701.
28. J. M. Dan, J. Mateus, Y. Kato, K. M. Hastie, E. D. Yu, C. E. Faliti, A. Grifoni, S. I. Ramirez, S. Haupt, A. Frazier, C. Nakao, V. Rayaprolu, S. A. Rawlings, B. Peters, F. Krammer, V. Simon, E. O. Saphire, D. M. Smith, D. Weiskopf, A. Sette, S. Crotty, Immunological memory to SARS-CoV-2 assessed for up to 8 months after infection. *Science* **371**, eabf4063 (2021). doi:10.1126/science.abf4063 Medline
29. K. W. Cohen, S. L. Linderman, Z. Moodie, J. Czartoski, L. Lai, G. Mantus, C. Norwood, L. E. Nyhoff, V. V. Edara, K. Floyd, S. C. De Rosa, H. Ahmed, R. Whaley, S. N. Patel, B. Prigmore, M. P. Lemos, C. W. Davis, S. Furth, J. B. O'Keefe, M. P. Gharpure, S. Gunisetty, K. Stephens, R. Antia, V. I. Zarnitsyna, D. S. Stephens, S. Edupuganti, N. Rouphael, E. J. Anderson, A. K. Mehta, J. Wrammert, M. S. Suthar, R. Ahmed, M. J. McElrath, Longitudinal analysis shows durable and broad immune memory after SARS-CoV-2 infection with persisting antibody responses and memory B and T cells. *Cell Rep. Med.* **2**, 100354 (2021). doi:10.1016/j.xcrm.2021.100354 Medline
30. D. A. Collier, A. De Marco, I. A. T. M. Ferreira, B. Meng, R. P. Datir, A. C. Walls, S. A. Kemp, J. Bassi, D. Pinto, C. Silacci-Fregni, S. Bianchi, M. A. Tortorici, J. Bowen, K. Culap, S. Jaconi, E. Cameroni, G. Snell, M. S. Pizzuto, A. F. Pellanda, C. Garzoni, A. Riva, The CITIID-NIHR BioResource COVID-19 Collaboration, A. Elmer, N. Kingston, B. Graves, L. E. McCoy, K. G. C. Smith, J. R. Bradley, N. Temperton, L. Ceron-Gutierrez, G. Barcenas-Morales, The COVID-19 Genomics UK (COG-UK) Consortium, W. Harvey, H. W. Virgin, A. Lanzavecchia, L. Piccoli, R. Doffinger, M. Wills, D. Veesler, D. Corti, R. K. Gupta, Sensitivity of SARS-CoV-2 B.1.1.7 to mRNA vaccine-elicited antibodies. *Nature* **593**, 136–141 (2021). doi:10.1038/s41586-021-03412-7
31. D. Zhou, W. Dejnirattisai, P. Supasa, C. Liu, A. J. Mentzer, H. M. Ginn, Y. Zhao, H. M. E. Duyvesteyn, A. Tuekprakhon, R. Nutalai, B. Wang, G. C. Paesen, C. Lopez-Camacho, J. Slon-Campos, B. Hallis, N. Coombes, K. Bewley, S. Charlton, T. S. Walter, D. Skelly, S. F. Lumley, C. Dold, R. Levin, T. Dong, A. J. Pollard, J. C. Knight, D. Crook, T. Lambe, E. Clutterbuck, S. Bibi, A. Flaxman, M. Bittaye, S. Belij-Rammerstorfer, S. Gilbert, W. James, M. W. Carroll, P. Klenerman, E. Barnes, S. J. Dunachie, E. E. Fry, J. Mongkolsapaya, J. Ren, D. I. Stuart, G. R. Screaton, Evidence of escape of SARS-CoV-2 variant B.1.351 from natural and vaccine-induced sera. *Cell* **184**, 2348–2361.e6 (2021). doi:10.1016/j.cell.2021.02.037 Medline
32. C. Liu, H. M. Ginn, W. Dejnirattisai, P. Supasa, B. Wang, A. Tuekprakhon, R. Nutalai, D. Zhou, A. J. Mentzer, Y. Zhao, H. M. E. Duyvesteyn, C. López-Camacho, J. Slon-Campos, T. S. Walter, D. Skelly, S. A. Johnson, T. G. Ritter, C. Mason, S. A. Costa

Clemens, F. Gomes Naveca, V. Nascimento, F. Nascimento, C. Fernandes da Costa, P. C. Resende, A. Pauvolid-Correa, M. M. Siqueira, C. Dold, N. Temperton, T. Dong, A. J. Pollard, J. C. Knight, D. Crook, T. Lambe, E. Clutterbuck, S. Bibi, A. Flaxman, M. Bittaye, S. Belij-Rammerstorfer, S. C. Gilbert, T. Malik, M. W. Carroll, P. Klenerman, E. Barnes, S. J. Dunachie, V. Baillie, N. Serafin, Z. Ditse, K. Da Silva, N. G. Paterson, M. A. Williams, D. R. Hall, S. Madhi, M. C. Nunes, P. Goulder, E. E. Fry, J. Mongkolsapaya, J. Ren, D. I. Stuart, G. R. Screaton, Reduced neutralization of SARS-CoV-2 B.1.617 by vaccine and convalescent serum. *Cell* **184**, 4220–4236.e13 (2021). doi:10.1016/j.cell.2021.06.020 Medline

33. W. F. Garcia-Beltran, E. C. Lam, K. St. Denis, A. D. Nitido, Z. H. Garcia, B. M. Hauser, J. Feldman, M. N. Pavlovic, D. J. Gregory, M. C. Poznansky, A. Sigal, A. G. Schmidt, A. J. Iafrate, V. Naranbhai, A. B. Balazs, Multiple SARS-CoV-2 variants escape neutralization by vaccine-induced humoral immunity. *Cell* **184**, 2372–2383.e9 (2021). doi:10.1016/j.cell.2021.03.013
34. L. Stamatatos, J. Czartoski, Y.-H. Wan, L. J. Homad, V. Rubin, H. Glantz, M. Neradilek, E. Seydoux, M. F. Jennewein, A. J. MacCamy, J. Feng, G. Mize, S. C. De Rosa, A. Finzi, M. P. Lemos, K. W. Cohen, Z. Moodie, M. J. McElrath, A. T. McGuire, mRNA vaccination boosts cross-variant neutralizing antibodies elicited by SARS-CoV-2 infection. *Science* **372**, 1413–1418 (2021). doi:10.1126/science.abg9175 Medline
35. C. J. Reynolds, C. Pade, J. M. Gibbons, D. K. Butler, A. D. Otter, K. Menacho, M. Fontana, A. Smit, J. E. Sackville-West, T. Cutino-Moguel, M. K. Maini, B. Chain, M. Noursadeghi, T. Brooks, A. Semper, C. Manisty, T. A. Treibel, J. C. Moon, A. M. Valdes, Á. McKnight, D. M. Altmann, R. Boyton, Prior SARS-CoV-2 infection rescues B and T cell responses to variants after first vaccine dose. *Science* **372**, 1418–1423 (2021). doi:10.1126/science.abh1282 Medline
36. Z. Wang, F. Muecksch, D. Schaefer-Babajew, S. Finkin, C. Viant, C. Gaebler, H. H. Hoffmann, C. O. Barnes, M. Cipolla, V. Ramos, T. Y. Oliveira, A. Cho, F. Schmidt, J. Da Silva, E. Bednarski, L. Aguado, J. Yee, M. Daga, M. Turroja, K. G. Millard, M. Jankovic, A. Gazumyan, Z. Zhao, C. M. Rice, P. D. Bieniasz, M. Caskey, T. Hatziioannou, M. C. Nussenzweig, Naturally enhanced neutralizing breadth against SARS-CoV-2 one year after infection. *Nature* **595**, 426–431 (2021). doi:10.1038/s41586-021-03696-9 Medline
37. A. H. Ellebedy, K. J. L. Jackson, H. T. Kissick, H. I. Nakaya, C. W. Davis, K. M. Roskin, A. K. McElroy, C. M. Oshansky, R. Elbein, S. Thomas, G. M. Lyon, C. F. Spiropoulou, A. K. Mehta, P. G. Thomas, S. D. Boyd, R. Ahmed, Defining antigen-specific plasmablast and memory B cell subsets in blood after viral infection or vaccination. *Nat. Immunol.* **17**, 1226–1234 (2016). doi:10.1038/ni.3533 Medline
38. A. Nellore, E. Zumaquero, C. D. Scharer, R. G. King, C. M. Tipton, C. F. Fucile, T. Mi, B. Mousseau, J. E. Bradley, F. Zhou, P. A. Goepfert, J. M. Boss, T. D. Randall, I. Sanz, A. F. Rosenberg, F. E. Lund, Influenza-specific effector memory B cells predict long-lived antibody responses to vaccination in humans. bioRxiv 643973 [Preprint] (2021). https://doi.org/10.1101/643973.
39. M. Jahnmatz, G. Kesa, E. Netterlid, A. M. Buisman, R. Thorstensson, N. Ahlborg, Optimization of a human IgG B-cell ELISpot assay for the analysis of vaccine-induced B-cell responses. *J. Immunol. Methods* **391**, 50–59 (2013). doi:10.1016/j.jim.2013.02.009 Medline
40. S. Gouma, M. E. Weirick, M. J. Bolton, C. P. Arevalo, E. C. Goodwin, E. M. Anderson, C. M. McAllister, S. R. Christensen, D. Dunbar, D. Fiore, A. Brock, J. Weaver, J. Millar, S. DerOhannessian, T. U. C. P. Unit, I. Frank, D. J. Rader, E. J. Wherry, S. E. Hensley, Health care worker seromonitoring reveals complex relationships between common coronavirus antibodies and COVID-19 symptom duration. *JCI Insight* **6**, e150449 (2021). doi:10.1172/jci.insight.150449 Medline
41. K. W. Ng, N. Faulkner, G. H. Cornish, A. Rosa, R. Harvey, S. Hussain, R. Ulferts, C. Earl, A. G. Wrobel, D. J. Benton, C. Roustan, W. Bolland, R. Thompson, A. Agua-Doce, P. Hobson, J. Heaney, H. Rickman, S. Paraskevopoulou, C. F. Houlihan, K. Thomson, E. Sanchez, G. Y. Shin, M. J. Spyer, D. Joshi, N. O'Reilly, P. A. Walker, S. Kjaer, A. Riddell, C. Moore, B. R. Jebson, M. Wilkinson, L. R. Marshall, E. C. Rosser, A. Radziszewska, H. Peckham, C. Ciurtin, L. R. Wedderburn, R. Beale, C. Swanton, S. Gandhi, B. Stockinger, J. McCauley, S. J. Gamblin, L. E. McCoy, P. Cherepanov, E. Nastouli, G. Kassiotis, Preexisting and de novo humoral immunity to SARS-CoV-2 in humans. *Science* **370**, 1339–1343 (2020). doi:10.1126/science.abe1107 Medline
42. P. Nguyen-Contant, A. K. Embong, P. Kanagaiah, F. A. Chaves, H. Yang, A. R. Branche, D. J. Topham, M. Y. Sangster, S protein-reactive IGG and memory B cell production after human SARS-CoV-2 infection includes broad reactivity to the S2 subunit. *mBio* **11**, 1–11 (2020). doi:10.1128/mBio.01991-20 Medline
43. J. Pallesen, N. Wang, K. S. Corbett, D. Wrapp, R. N. Kirchdoerfer, H. L. Turner, C. A. Cottrell, M. M. Becker, L. Wang, W. Shi, W.-P. Kong, E. L. Andres, A. N. Kettenbach, M. R. Denison, J. D. Chappell, B. S. Graham, A. B. Ward, J. S. McLellan, Immunogenicity and structures of a rationally designed prefusion MERS-CoV spike antigen. *Proc. Natl. Acad. Sci. U.S.A.* **114**, E7348–E7357 (2017). doi:10.1073/pnas.1707304114 Medline
44. K. S. Corbett, D. K. Edwards, S. R. Leist, O. M. Abiona, S. Boyoglu-Barnum, R. A. Gillespie, S. Himansu, A. Schäfer, C. T. Ziwawo, A. T. DiPiazza, K. H. Dinnon, S. M. Elbashir, C. A. Shaw, A. Woods, E. J. Fritch, D. R. Martinez, K. W. Bock, M. Minai, B. M. Nagata, G. B. Hutchinson, K. Wu, C. Henry, K. Bahl, D. Garcia-Dominguez, L. Ma, I. Renzi, W.-P. Kong, S. D. Schmidt, L. Wang, Y. Zhang, E. Phung, L. A. Chang, R. J. Loomis, N. E. Altaras, E. Narayanan, M. Metkar, V. Presnyak, C. Liu, M. K. Louder, W. Shi, K. Leung, E. S. Yang, A. West, K. L. Gully, L. J. Stevens, N. Wang, D. Wrapp, N. A. Doria-Rose, G. Stewart-Jones, H. Bennett, G. S. Alvarado, M. C. Nason, T. J. Ruckwardt, J. S. McLellan, M. R. Denison, J. D. Chappell, I. N. Moore, K. M. Morabito, J. R. Mascola, R. S. Baric, A. Carfi, B. S. Graham, SARS-CoV-2 mRNA vaccine design enabled by prototype pathogen preparedness. *Nature* **586**, 567–571 (2020). doi:10.1038/s41586-020-2622-0 Medline
45. T. J. C. Tan, M. Yuan, K. Kuzelka, G. C. Padron, J. R. Beal, X. Chen, Y. Wang, J. Rivera-Cardona, X. Zhu, B. M. Stadtmueller, C. B. Brooke, I. A. Wilson, N. C. Wu, Sequence signatures of two public antibody clonotypes that bind SARS-CoV-2 receptor binding domain. *Nat. Commun.* **12**, 3815 (2021). doi:10.1038/s41467-021-24123-7 Medline
46. H. L. Dugan, C. T. Stamper, L. Li, S. Changrob, N. W. Asby, P. J. Halfmann, N.-Y. Zheng, M. Huang, D. G. Shaw, M. S. Cobb, S. A. Erickson, J. J. Guthmiller, O. Stovicek, J. Wang, E. S. Winkler, M. L. Madariaga, K. Shanmugarajah, M. O. Jansen, F. Amanat, I. Stewart, H. A. Utset, J. Huang, C. A. Nelson, Y.-N. Dai, P. D. Hall, R. P. Jedrzejczak, A. Joachimiak, F. Krammer, M. S. Diamond, D. H. Fremont, Y. Kawaoka, P. C. Wilson, Profiling B cell immunodominance after SARS-CoV-2 infection reveals antibody evolution to non-neutralizing viral targets. *Immunity* **54**, 1290–1303.e7 (2021). doi:10.1016/j.immuni.2021.05.001 Medline
47. A. M. Rosenfeld, W. Meng, D. Y. Chen, B. Zhang, T. Granot, D. L. Farber, U. Hershberg, E. T. Luning Prak, Computational evaluation of B-cell clone sizes in bulk populations. *Front. Immunol.* **9**, 1472 (2018). doi:10.3389/fimmu.2018.01472 Medline
48. C. Gaebler, Z. Wang, J. C. C. Lorenzi, F. Muecksch, S. Finkin, M. Tokuyama, A. Cho, M. Jankovic, D. Schaefer-Babajew, T. Y. Oliveira, M. Cipolla, C. Viant, C. O. Barnes, Y. Bram, G. Breton, T. Hägglöf, P. Mendoza, A. Hurley, M. Turroja, K. Gordon, K. G. Millard, V. Ramos, F. Schmidt, Y. Weisblum, D. Jha, M. Tankelevich, G. Martinez-Delgado, J. Yee, R. Patel, J. Dizon, C. Unson-O'Brien, I. Shimeliovich, D. F. Robbiani, Z. Zhao, A. Gazumyan, R. E. Schwartz, T. Hatziioannou, P. J. Bjorkman, S. Mehandru, P. D. Bieniasz, M. Caskey, M. C. Nussenzweig, Evolution of antibody immunity to SARS-CoV-2. *Nature* **591**, 639–644 (2021). doi:10.1038/s41586-021-03207-w Medline
49. M. G. de Mattos Barbosa, H. Liu, D. Huynh, G. Shelley, E. T. Keller, B. T. Emmer, E. Sherman, D. Ginsburg, A. A. Kennedy, A. W. Tai, C. Wobus, C. Mirabeli, T. M. Lanigan, M. Samaniego, W. Meng, A. M. Rosenfeld, E. T. Luning Prak, J. L. Platt, M. Cascalho, IgV somatic mutation of human anti-SARS-CoV-2 monoclonal antibodies governs neutralization and breadth of reactivity. *JCI Insight* **6**, e147386 (2021). doi:10.1172/jci.insight.147386 Medline
50. D. Geers, M. C. Shamier, S. Bogers, G. den Hartog, L. Gommers, N. N. Nieuwkoop, K. S. Schmitz, L. C. Rijsbergen, J. A. T. van Osch, E. Dijkhuizen, G. Smits, A. Comvalius, D. van Mourik, T. G. Caniels, M. J. van Gils, R. W. Sanders, B. B. O. Munnink, R. Molenkamp, H. J. de Jager, B. L. Haagmans, R. L. de Swart, M. P. G. Koopmans, R. S. van Binnendijk, R. D. de Vries, C. H. GeurtsvanKessel, SARS-CoV-2 variants of concern partially escape humoral but not T cell responses in COVID-19 convalescent donors and vaccine recipients. *Sci. Immunol.* **6**, eabj1750 (2021). doi:10.1126/sciimmunol.abj1750
51. A. Tarke, J. Sidney, C. K. Kidd, J. M. Dan, S. I. Ramirez, E. D. Yu, J. Mateus, R. da Silva Antunes, E. Moore, P. Rubiro, N. Methot, E. Phillips, S. Mallal, A. Frazier, S. A. Rawlings, J. A. Greenbaum, B. Peters, D. M. Smith, S. Crotty, D. Weiskopf, A. Grifoni, A. Sette, Comprehensive analysis of T cell immunodominance and immunoprevalence of SARS-CoV-2 epitopes in COVID-19 cases. *Cell Rep. Med.* **2**,

100204 (2021). doi:10.1016/j.xcrm.2021.100204 Medline
52. A. Grifoni, D. Weiskopf, S. I. Ramirez, J. Mateus, J. M. Dan, C. R. Moderbacher, S. A. Rawlings, A. Sutherland, L. Premkumar, R. S. Jadi, D. Marrama, A. M. de Silva, A. Frazier, A. F. Carlin, J. A. Greenbaum, B. Peters, F. Krammer, D. M. Smith, S. Crotty, A. Sette, Targets of T Cell Responses to SARS-CoV-2 Coronavirus in Humans with COVID-19 Disease and Unexposed Individuals. *Cell* **181**, 1489–1501.e15 (2020). doi:10.1016/j.cell.2020.05.015 Medline
53. S. M. Kaech, E. J. Wherry, R. Ahmed, Effector and memory T-cell differentiation: Implications for vaccine development. *Nat. Rev. Immunol.* **2**, 251–262 (2002). doi:10.1038/nri778 Medline
54. D. D. Flannery, S. Gouma, M. B. Dhudasia, S. Mukhopadhyay, M. R. Pfeifer, E. C. Woodford, J. S. Gerber, C. P. Arevalo, M. J. Bolton, M. E. Weirick, E. C. Goodwin, E. M. Anderson, A. R. Greenplate, J. Kim, N. Han, A. Pattekar, J. Dougherty, O. Kuthuru, D. Mathew, A. E. Baxter, L. A. Vella, J. Weaver, A. Verma, R. Leite, J. S. Morris, D. J. Rader, M. A. Elovitz, E. J. Wherry, K. M. Puopolo, S. E. Hensley, SARS-CoV-2 seroprevalence among parturient women in Philadelphia. *Sci. Immunol.* **5**, eabd5709 (2020). doi:10.1126/sciimmunol.abd5709 Medline
55. M. Roederer, J. L. Nozzi, M. C. Nason, SPICE: Exploration and analysis of post-cytometric complex multivariate datasets. *Cytometry A* **79**, 167–174 (2011). doi:10.1002/cyto.a.21015 Medline
56. W. Meng, B. Zhang, G. W. Schwartz, A. M. Rosenfeld, D. Ren, J. J. C. Thome, D. J. Carpenter, N. Matsuoka, H. Lerner, A. L. Friedman, T. Granot, D. L. Farber, M. J. Shlomchik, U. Hershberg, E. T. Luning Prak, An atlas of B-cell clonal distribution in the human body. *Nat. Biotechnol.* **35**, 879–884 (2017). doi:10.1038/nbt.3942 Medline
57. L. Kuri-Cervantes, M. B. Pampena, W. Meng, A. M. Rosenfeld, C. A. G. Ittner, A. R. Weisman, R. S. Agyekum, D. Mathew, A. E. Baxter, L. A. Vella, O. Kuthuru, S. A. Apostolidis, L. Bershaw, J. Dougherty, A. R. Greenplate, A. Pattekar, J. Kim, N. Han, S. Gouma, M. E. Weirick, C. P. Arevalo, M. J. Bolton, E. C. Goodwin, E. M. Anderson, S. E. Hensley, T. K. Jones, N. S. Mangalmurti, E. T. Luning Prak, E. J. Wherry, N. J. Meyer, M. R. Betts, Comprehensive mapping of immune perturbations associated with severe COVID-19. *Sci. Immunol.* **5**, eabd7114 (2020). doi:10.1126/sciimmunol.abd7114 Medline
58. J. A. Vander Heiden, G. Yaari, M. Uduman, J. N. H. Stern, K. C. O'Connor, D. A. Hafler, F. Vigneault, S. H. Kleinstein, pRESTO: A toolkit for processing high-throughput sequencing raw reads of lymphocyte receptor repertoires. *Bioinformatics* **30**, 1930–1932 (2014). doi:10.1093/bioinformatics/btu138 Medline
59. J. Ye, N. Ma, T. L. Madden, J. M. Ostell, IgBLAST: An immunoglobulin variable domain sequence analysis tool. *Nucleic Acids Res.* **41**, W34–W40 (2013). doi:10.1093/nar/gkt382 Medline
60. A. M. Rosenfeld, W. Meng, E. T. Luning Prak, U. Hershberg, ImmuneDB: A system for the analysis and exploration of high-throughput adaptive immune receptor sequencing data. *Bioinformatics* **33**, 292–293 (2017). doi:10.1093/bioinformatics/btw593 Medline
61. A. M. Rosenfeld, W. Meng, E. T. Luning Prak, U. Hershberg, ImmuneDB, a Novel Tool for the Analysis, Storage, and Dissemination of Immune Repertoire Sequencing Data. *Front. Immunol.* **9**, 2107 (2018). doi:10.3389/fimmu.2018.02107 Medline
62. J. Huerta-Cepas, F. Serra, P. Bork, ETE 3: Reconstruction, Analysis, and Visualization of Phylogenomic Data. *Mol. Biol. Evol.* **33**, 1635–1638 (2016). doi:10.1093/molbev/msw046 Medline
63. B. D. Corrie, N. Marthandan, B. Zimonja, J. Jaglale, Y. Zhou, E. Barr, N. Knoetze, F. M. W. Breden, S. Christley, J. K. Scott, L. G. Cowell, F. Breden, iReceptor: A platform for querying and analyzing antibody/B-cell and T-cell receptor repertoire data across federated repositories. *Immunol. Rev.* **284**, 24–41 (2018). doi:10.1111/imr.12666 Medline
64. F. Vaida, L. Liu, Fast Implementation for Normal Mixed Effects Models With Censored Response. *J. Comput. Graph. Stat.* **18**, 797–817 (2009). doi:10.1198/jcgs.2009.07130 Medline
65. D. Mathew, J. R. Giles, A. E. Baxter, D. A. Oldridge, A. R. Greenplate, J. E. Wu, C. Alanio, L. Kuri-Cervantes, M. B. Pampena, K. D'Andrea, S. Manne, Z. Chen, Y. J. Huang, J. P. Reilly, A. R. Weisman, C. A. G. Ittner, O. Kuthuru, J. Dougherty, K. Nzingha, N. Han, J. Kim, A. Pattekar, E. C. Goodwin, E. M. Anderson, M. E. Weirick, S. Gouma, C. P. Arevalo, M. J. Bolton, F. Chen, S. F. Lacey, H. Ramage, S. Cherry, S. E. Hensley, S. A. Apostolidis, A. C. Huang, L. A. Vella, The UPenn COVID Processing Unit, M. R. Betts, N. J. Meyer, E. J. Wherry, Deep immune profiling of COVID-19 patients reveals distinct immunotypes with therapeutic implications. *Science* **369**, eabc8511 (2020). doi:10.1126/science.abc8511 Medline

ACKNOWLEDGMENTS

We thank the study participants for their generosity in making the study possible. We also thank S. Crotty and members of the Wherry lab for helpful discussions and feedback, as well as the Flow Cytometry Core and the Human Immunology Core at the University of Pennsylvania for technical support. **Funding:** This work was supported by grants from the NIH AI105343, AI082630, AI108545, AI155577, AI149680 (to EJW), AI152236, AI142638 (to PB), HL143613 (to JRG), P30-AI0450080 (to ELP), R38 HL143613 (to DAO), T32 AR076951-01 (to SAA), T32 CA009140 (to JRG, DAO, and DM), T32 AI055400 (to PH), U19AI082630 (to SEH and EJW), NIH contract Nr. 75N9301900065 (to DW, AS), Australian government Medical Research Future Fund awards GNT2002073 (MPD), MRF2005544 (MPD), MRF2005760 (to MPD), an NHMRC program grant GNT1149990 (MPD), NHMRC Fellowship / Investigator grants (DSK, MPD), funding from the National Health and Medical Research Council of Australia and the Australian Research Council (DSK), funding from the Allen Institute for Immunology (to SAA, EJW), Cancer Research Institute-Mark Foundation Fellowship (to JRG), Chen Family Research Fund (to SAA), the Parker Institute for Cancer Immunotherapy (to JRG, EJW), funding from Moderna and Janssen (IF), the Penn Center for Research on Coronavirus and Other Emerging Pathogens (to PB), the University of Pennsylvania Perelman School of Medicine COVID Fund (to RRG, EJW), the University of Pennsylvania Perelman School of Medicine 21st Century Scholar Fund (to RRG), and a philanthropic gift from Jeffrey Lurie, Joel Embiid, Josh Harris, and David Blitzer (to SEH). **Author contributions:** RRG, MMP, and EJW designed the study. RRG, MMP, SAA, DM, WM, KL, SG, LKC, PH, SD, MEW, CMM, MA, NT, and EMD carried out experiments. RRG, SAA, JD, SL, and OK were involved in clinical recruitment and sample collection. WM, AMR, AR, DSK, DAO, JRG, MPD, and ELP provided expertise on statistical analyses. RRG, MMP, DM, and AEB contributed to the methodology. RRG, MMP, AP, AH, HS, SH, SK, JTH, JCW, and SA processed peripheral blood samples and managed the sample database. IF, AG, DW, and AS provided key samples and/or reagents. ELP, ARG and EJW supervised the study. All authors participated in data analysis and interpretation. RRG, MMP, ELP, and EJW wrote the manuscript. **Competing interests:** AS is a consultant for Gritstone, Flow Pharma, CellCarta, Arcturus, Oxfordimmunotech, and Avalia. La Jolla Institute for Immunology has filed for patent protection for various aspects of T cell epitope and vaccine design work. SEH has received consultancy fees from Sanofi Pasteur, Lumen, Novavax, and Merk for work unrelated to this report. ELP is consulting or an advisor for Roche Diagnostics, Enpicom, The Antibody Society, IEDB, and The American Autoimmune Related Diseases Association. ARG is a consultant for Relation Therapeutics. MRB is a consultant for Interius Biotherapeutics. IF receives research funding from Moderna and Janssen. EJW is consulting or is an advisor for Merck, Marengo, Janssen, Related Sciences, Synthekine and Surface Oncology. EJW is a founder of Surface Oncology, Danger Bio and Arsenal Biosciences. **Data and materials availability:** All data, code, and materials used in this manuscript are available from the authors upon reasonable request. This work is licensed under a Creative Commons Attribution 4.0 International (CC BY 4.0) license, which permits unrestricted use, distribution, and reproduction in any medium, provided the original work is properly cited. To view a copy of this license, visit https://creativecommons.org/licenses/by/4.0/. This license does not apply to figures/photos/artwork or other content included in the article that is credited to a third party; obtain authorization from the rights holder before using such material.

The UPenn COVID Processing Unit

S. Adamski, Z. Alam, M. M. Addison, K. T. Byrne, A. Chandra, H. C. Descamps, N. Han, Y. Kaminskiy, S. C. Kammerman, J. Kim, A. R. Greenplate, J. T. Hamilton, N. Markosyan, J. Han Noll, D. K. Omran, A. Pattekar, E. Perkey, E. M. Prager, D. Pueschl, A. Rennels, J. B. Shah, J. S. Shilan, N. Wilhausen, A. N. Vanderbeck

University of Pennsylvania Perelman School of Medicine, Philadelphia, PA, USA.

SUPPLEMENTARY MATERIALS
science.org/doi/10.1126/science.abm0829
Figs. S1 to S7
Tables S1 to S5

26 August 2021; accepted 10 October 2021
Published online 14 October 2021
10.1126/science.abm0829

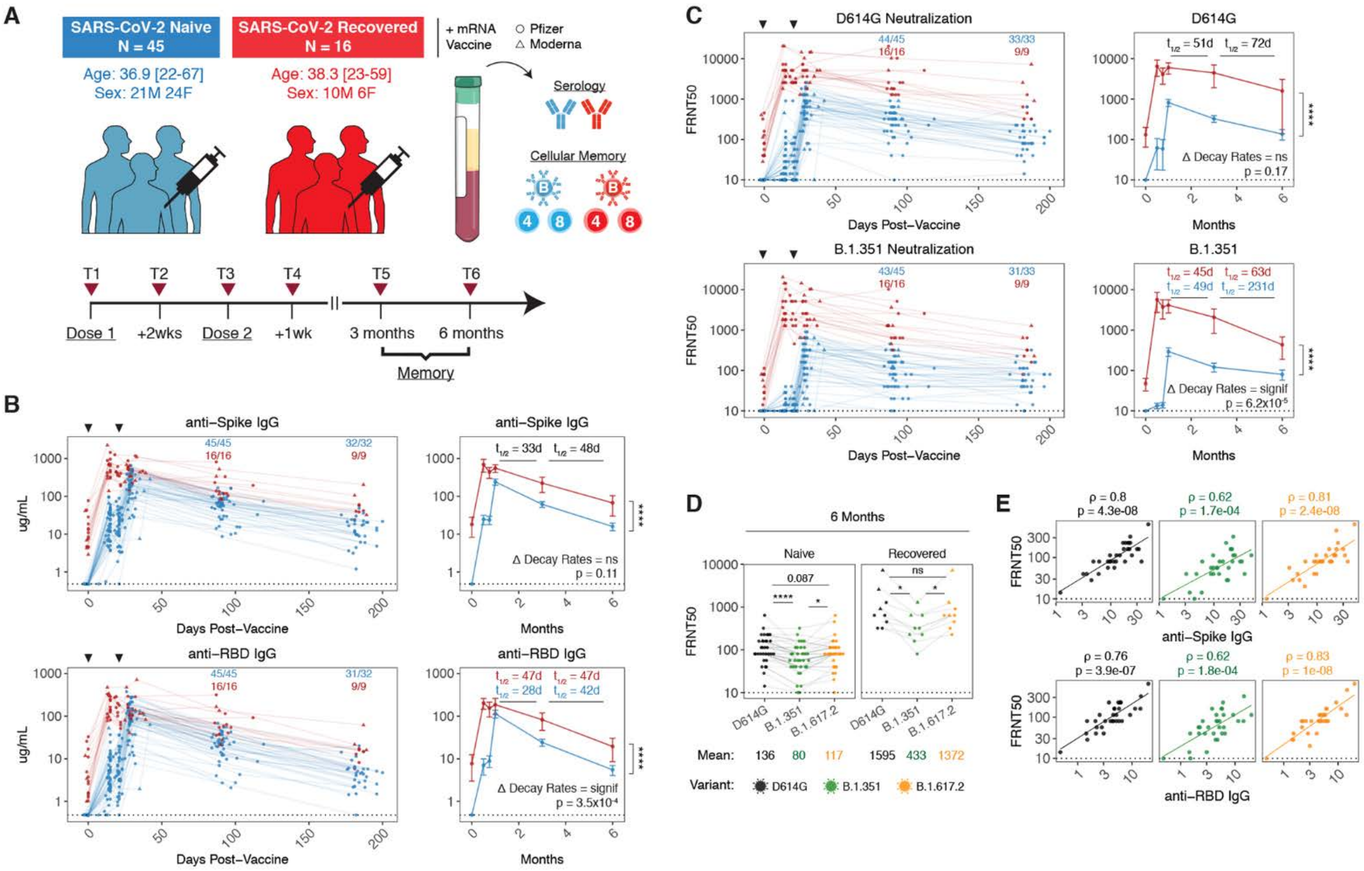

Fig. 1. SARS-CoV-2 mRNA vaccines induce robust antibody responses. (**A**) University of Pennsylvania COVID-19 vaccine study design and cohort summary statistics. (**B**) Anti-Spike and anti-RBD IgG concentrations over time in plasma samples from vaccinated individuals. (**C**) Pseudovirus neutralization titers against wild-type D614G or B.1.351 variant Spike protein over time in plasma samples from vaccinated individuals. Data are represented as focus reduction neutralization titer 50% (FRNT50) values. (**D**) Comparison of D614G, B.1.351, and B.1.617.2 FRNT50 values at 6 months post-vaccination. (**E**) Correlation between anti-Spike or anti-RBD IgG and neutralizing titers (D614G = black, B.1.351 = green, B.1.617.2 = orange; statistics were calculated using non-parametric Spearman rank correlation). Dotted lines indicate the limit of detection for the assay. For B and C, black triangles indicate time of vaccine doses, fractions above plots indicate the number of individuals above their individual baseline at memory timepoints, and summary plots show mean values with the 95% confidence interval. Decay rates were calculated using a piecewise linear mixed effects model with censoring. Changes in decay rate over time (linear vs. 2-phase decay) were determined based on a likelihood ratio test. Δ Decay Rates indicates whether decay rates were different in SARS-CoV-2 naïve and recovered groups. Statistics were calculated using unpaired (B and C) or paired (D) non-parametric Wilcoxon test with BH correction. Blue and red values indicate comparisons within naïve or recovered groups. * = p < 0.05, ** = p < 0.01, *** = p < 0.001, **** = p < 0.0001, ns = not significant.

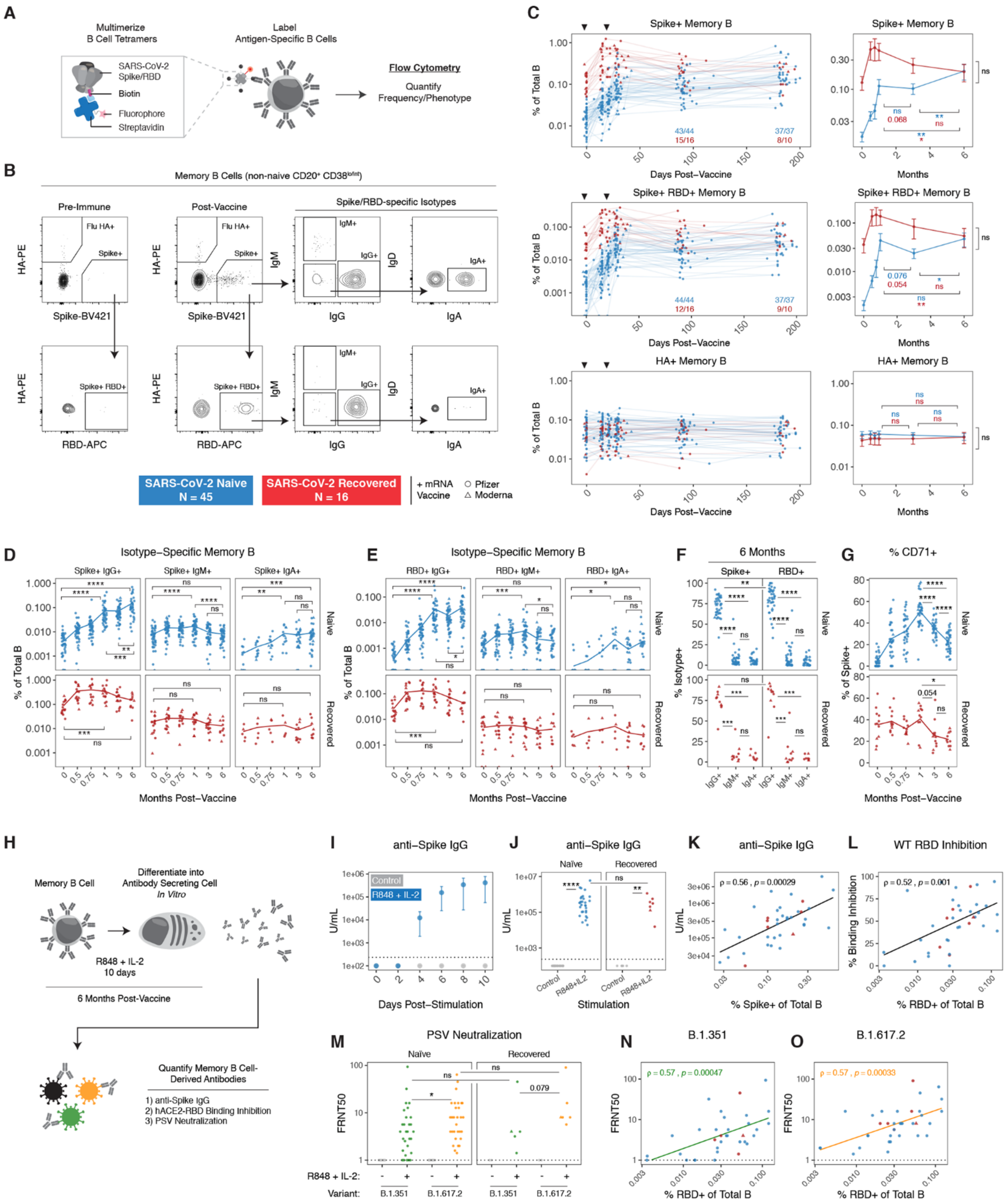
A
Multimerize B Cell Tetramers
Label Antigen-Specific B Cells
SARS-CoV-2 Spike/RBD
Biotin
Fluorophore
Streptavidin
Flow Cytometry
Quantify Frequency/Phenotype
B
Memory B Cells (non-naive CD20+ CD38lo/int)
Pre-Immune
Post-Vaccine
Spike/RBD-specific Isotypes
SARS-CoV-2 Naive N = 45
SARS-CoV-2 Recovered N = 16
+ mRNA Vaccine
Pfizer
Moderna
C
Spike+ Memory B
Spike+ RBD+ Memory B
HA+ Memory B
Days Post−Vaccine
Months
% of Total B
D
Isotype−Specific Memory B
E
Isotype−Specific Memory B
F
6 Months
G
% CD71+
Months Post−Vaccine
Naive
Recovered
H
Memory B Cell
Differentiate into Antibody Secreting Cell In Vitro
R848 + IL-2 10 days
6 Months Post-Vaccine
Quantify Memory B Cell-Derived Antibodies
1) anti-Spike IgG
2) hACE2-RBD Binding Inhibition
3) PSV Neutralization
I
anti−Spike IgG
Days Post−Stimulation
J
anti−Spike IgG
Stimulation
K
anti−Spike IgG
% Spike+ of Total B
L
WT RBD Inhibition
% RBD+ of Total B
M
PSV Neutralization
N
B.1.351
O
B.1.617.2
FRNT50
% RBD+ of Total B

Fig. 2. SARS-CoV-2 mRNA vaccines generate durable and functional memory B cell responses. (**A**) Experimental design and (**B**) Gating strategy for quantifying the frequency and phenotype of SARS-CoV-2-specific memory B cells by flow cytometry. Antigen specificity was determined based on binding to fluorophore-labeled Spike, RBD, and influenza HA tetramers. (**C**) Frequencies of SARS-CoV-2 Spike+, Spike+ RBD+, and influenza HA+ memory B cells over time in PBMC samples from vaccinated individuals. Data are represented as a percentage of total B cells, black triangles indicate time of vaccine doses, fractions below plots indicate the number of individuals above their individual baseline at memory timepoints, and summary plots show mean values with the 95% confidence interval. (**D**) Frequency of isotype-specific Spike+ and (**E**) Spike+ RBD+ memory B cells over time. IgA was assessed on a subset of subjects. (**F**) Percent IgG+, IgM+, or IgA+ of SARS-CoV-2-specific memory B cells at 6 months post-vaccination. (**G**) Percent CD71+ of total Spike+ memory B cells over time. (**H**) Experimental design for in vitro differentiation of memory B cells into antibody secreting cells. (**I**) anti-Spike IgG levels in culture supernatants over time from PBMCs stimulated with PBS control or R848 + IL-2 (n=4). (**J**) anti-Spike IgG levels in culture supernatants after 10 days of stimulation (**K**) Correlation of Spike+ memory B cell frequencies by flow cytometry with anti-Spike IgG levels from in vitro stimulation. (**L**) Correlation of RBD+ memory B cell frequencies by flow cytometry with hACE2-RBD-binding inhibition from in vitro stimulation. (**M**) Pseudovirus (PSV) neutralizing titers against B.1.351 and B.1.617.2 variants in culture supernatants after 10 days of stimulation. (**N**) Correlation of RBD+ memory B cell frequencies by flow cytometry with PSV neutralizing titers of memory B cell-derived antibodies against B.1.351 and (**O**) B.1.617.2. For D, E, and G, lines connect mean values at different timepoints. For K, L, N, and O, correlations were calculated using non-parametric Spearman rank correlation. Dotted lines indicate the limit of detection of the assay. Statistics were calculated using unpaired non-parametric Wilcoxon test with BH correction for multiple comparisons. Blue and red values indicate comparisons within naïve or recovered groups. * = $p < 0.05$, ** = $p < 0.01$, *** = $p < 0.001$, **** = $p < 0.0001$, ns = not significant.

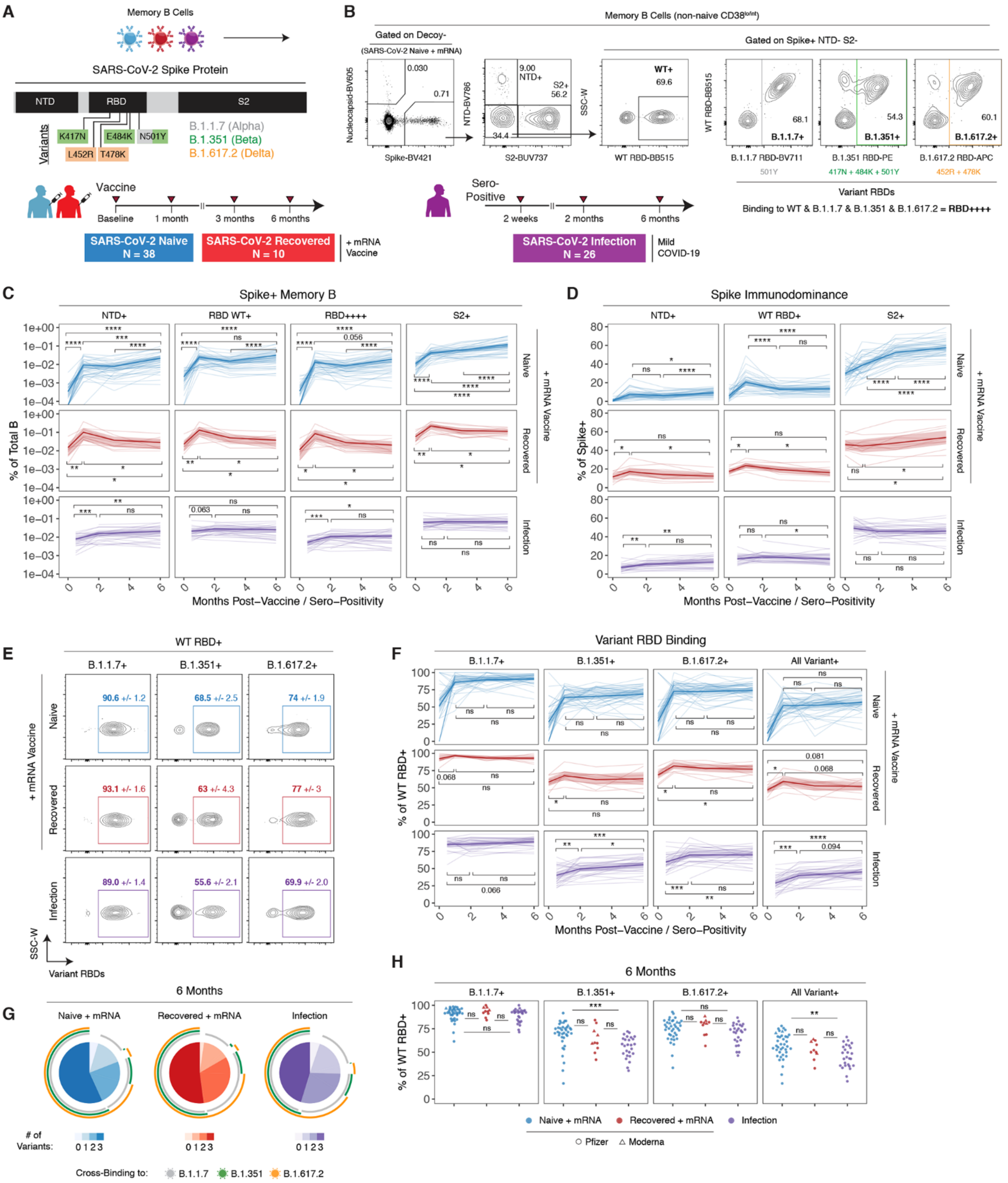
A
Memory B Cells
SARS-CoV-2 Spike Protein
NTD
RBD
S2
Variants
K417N
E484K
N501Y
L452R
T478K
B.1.1.7 (Alpha)
B.1.351 (Beta)
B.1.617.2 (Delta)
Vaccine
Baseline
1 month
3 months
6 months
SARS-CoV-2 Naive N = 38
SARS-CoV-2 Recovered N = 10
+ mRNA Vaccine
Sero-Positive
2 weeks
2 months
6 months
SARS-CoV-2 Infection N = 26
Mild COVID-19
B
Memory B Cells (non-naive CD38lo/int)
Gated on Decoy-
(SARS-CoV-2 Naive + mRNA)
Gated on Spike+ NTD- S2-
Nucleocapsid-BV605
Spike-BV421
NTD-BV786
S2-BUV737
SSC-W
WT RBD-BB515
WT+
B.1.1.7+
B.1.351+
B.1.617.2+
B.1.1.7 RBD-BV711
B.1.351 RBD-PE
B.1.617.2 RBD-APC
501Y
417N + 484K + 501Y
452R + 478K
Variant RBDs
Binding to WT & B.1.1.7 & B.1.351 & B.1.617.2 = RBD++++
C
Spike+ Memory B
NTD+
RBD WT+
RBD++++
S2+
% of Total B
Naive
Recovered
Infection
+ mRNA Vaccine
Months Post–Vaccine / Sero–Positivity
D
Spike Immunodominance
NTD+
WT RBD+
S2+
% of Spike+
Months Post–Vaccine / Sero–Positivity
E
WT RBD+
B.1.1.7+
B.1.351+
B.1.617.2+
+ mRNA Vaccine
Naive
Recovered
Infection
90.6 +/- 1.2
68.5 +/- 2.5
74 +/- 1.9
93.1 +/- 1.6
63 +/- 4.3
77 +/- 3
89.0 +/- 1.4
55.6 +/- 2.1
69.9 +/- 2.0
SSC-W
Variant RBDs
F
Variant RBD Binding
B.1.1.7+
B.1.351+
B.1.617.2+
All Variant+
% of WT RBD+
Months Post–Vaccine / Sero–Positivity
G
6 Months
Naive + mRNA
Recovered + mRNA
Infection
# of Variants:
0 1 2 3
Cross-Binding to:
B.1.1.7
B.1.351
B.1.617.2
H
6 Months
B.1.1.7+
B.1.351+
B.1.617.2+
All Variant+
% of WT RBD+
Naive + mRNA
Recovered + mRNA
Infection
Pfizer
Moderna

Fig. 3. Memory B cells induced by mRNA vaccination or infection are cross-reactive to SARS-CoV-2 variants of concern and increase in frequency over time. (**A**) Experimental design and (**B**) Gating strategy for quantifying the frequency and phenotype of Spike subunit and variant-specific memory B cells by flow cytometry. Specific mutations in B.1.1.7, B.1.351, or B.1.617.2 variant RBDs are indicated. (**C**) Frequencies of Spike+ NTD+, Spike+ WT RBD+, Spike+ RBD++++ (all variant binding), and Spike+ S2+ memory B cells over time in PBMC samples from vaccinated or convalescent individuals. Data are represented as a percentage of total B cells. (**D**) Percent NTD+, RBD+, or S2+ of total Spike+ memory B cells over time. (**E**) Representative plots of variant RBD cross-binding gated on Spike+ WT RBD+ cells in vaccinated or convalescent individuals. Mean and standard error values at the 6-month timepoint are indicated. (**F**) Percent B.1.1.7+, B.1.351+, B.1.617.2+, or all variant+ of WT RBD+ memory B cells over time. (**G**) Boolean analysis of variant cross-binding memory B cell populations in vaccinated, infected then vaccinated, or infected only individuals at 6 months post-vaccination/seropositivity. Pie charts indicate the fraction of WT RBD+ memory B cells that cross-bind 0, 1, 2, or 3 variant RBDs. Colored arcs indicate cross-binding to specific variants. (**H**) Cross-sectional analysis of variant binding as a percentage of WT RBD+ memory B cells at 6 months post-vaccination/seropositivity. For C, D, and F, thick lines indicate mean values and thin lines represent individual subjects. Statistics were calculated using paired (C, D, and F) or unpaired (H) non-parametric Wilcoxon test with BH correction for multiple comparisons. Blue, red, and purple values indicate comparisons within naïve, recovered, or infection only groups. * = $p < 0.05$, ** = $p < 0.01$, *** = $p < 0.001$, **** = $p < 0.0001$, ns = not significant.

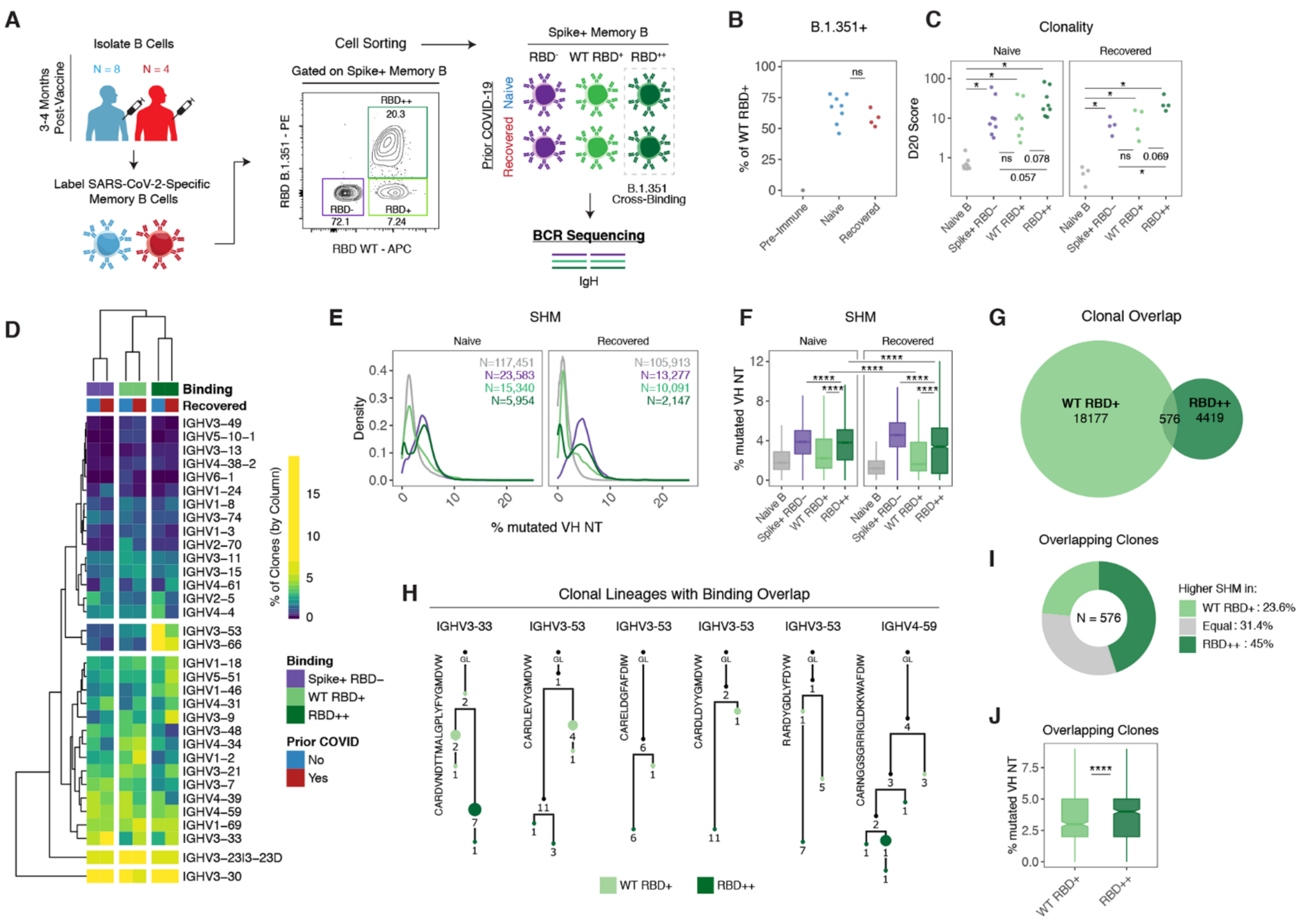

Fig. 4. Variant-binding memory B cell clones use distinct VH genes and evolve through somatic hypermutation. (**A**) Experimental design for sorting and sequencing SARS-CoV-2-specific memory B cells. (**B**) Frequency of RBD++ (B.1.351 variant cross-binding) memory B cells as a percentage of total RBD+ cells. (**C**) Percentage of sequence copies occupied by the top 20 ranked clones (D20) across naïve B cells and different antigen-binding memory B cell populations. (**D**) Heatmap and hierarchical clustering of VH gene usage frequencies in memory B cell clones across different antigen-binding populations. Data are represented as the percent of clones with the indicated VH gene per column. (**E**) Somatic hypermutation (SHM) density plots (bin width = 1) and (**F**) boxplots of individual clones across naïve B cells and different antigen-binding memory B cell populations. Data are represented as the percent of mutated VH nucleotides. Number of clones sampled for each population is indicated. For C-F, data were filtered on clones with productive rearrangements and ≥ 2 copies. (**G**) Venn diagram of clonal lineages that are shared between WT RBD and RBD cross-binding (RBD++) populations. Data were filtered based on larger clones with ≥ 50% mean copy number frequency (mcf) in each sequencing library. (**H**) Example lineage trees of clones with overlapping binding to WT and B.1.351 variant RBD. VH genes and CDR3 sequences are indicated. Numbers refer to mutations compared to the preceding vertical node. Colors indicate binding specificity, black dots indicate inferred nodes, and size is proportional to sequence copy number; GL = germline sequence. (**I**) Classification of SHM within overlapping clones. Each clone was defined as having higher (or equal) SHM in WT RBD binders or RBD++ cross-binders based on average levels of SHM for all WT RBD vs. RBD++ sequence variant copies within each lineage. (**J**) SHM levels within overlapping clones. Data are represented as the percent of mutated VH nucleotides for WT RBD and RBD++ sequence copies. Statistics were calculated using paired non-parametric Wilcoxon test, with BH correction for multiple comparisons in C and F. Notches on boxplots in F and J indicate a 95% confidence interval of the median. * = p < 0.05, ** = p < 0.01, *** = p < 0.001, **** = p < 0.0001, ns = not significant.

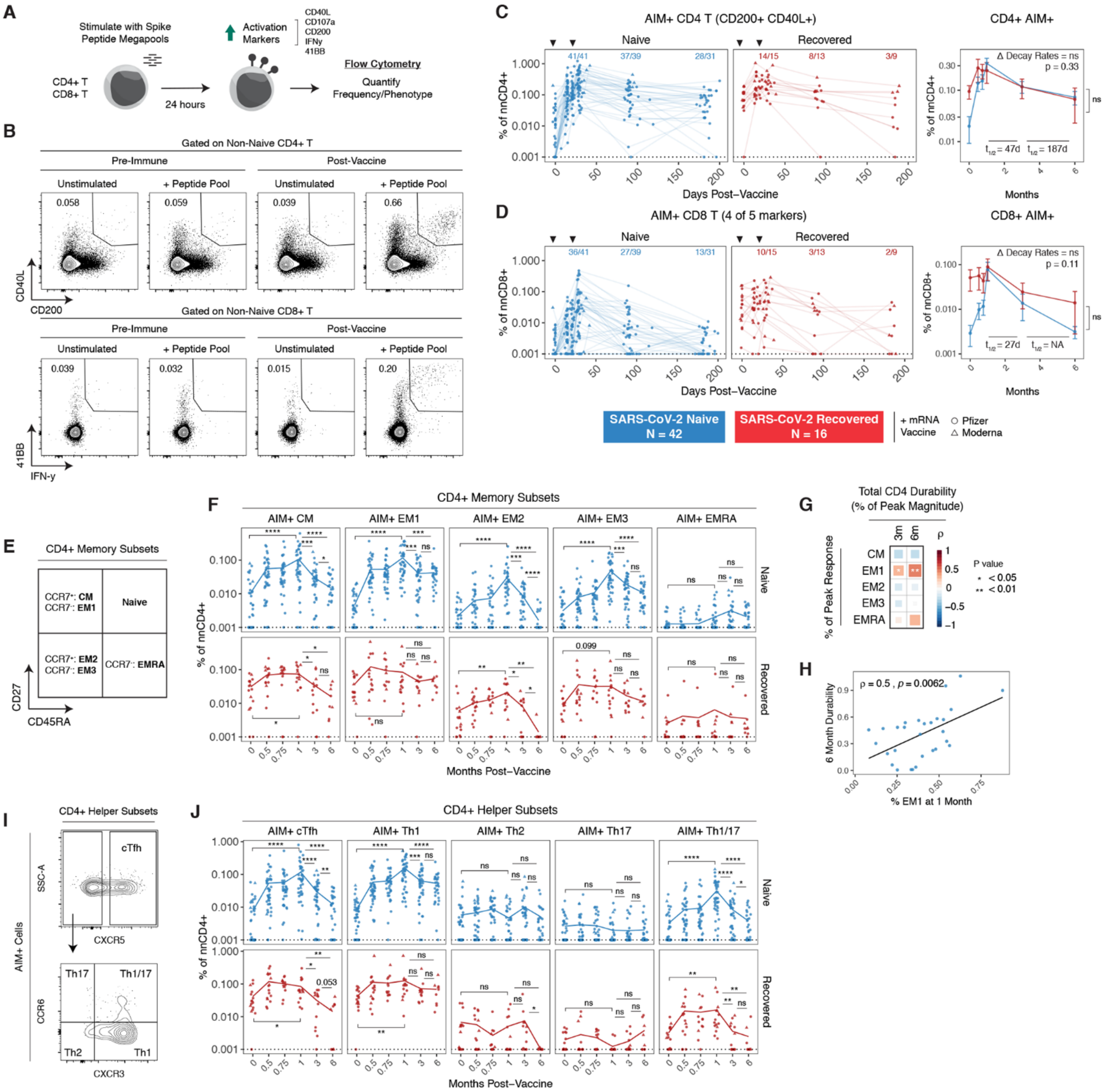

A
Stimulate with Spike Peptide Megapools
CD4+ T
CD8+ T
24 hours
Activation Markers
CD40L
CD107a
CD200
IFNy
41BB
Flow Cytometry
Quantify Frequency/Phenotype
B
Gated on Non-Naive CD4+ T
Pre-Immune
Post-Vaccine
Unstimulated
+ Peptide Pool
0.058
0.059
0.039
0.66
CD40L
CD200
Gated on Non-Naive CD8+ T
0.039
0.032
0.015
0.20
41BB
IFN-y
C
AIM+ CD4 T (CD200+ CD40L+)
Naive
Recovered
41/41
37/39
28/31
14/15
8/13
3/9
% of nnCD4+
Days Post–Vaccine
CD4+ AIM+
Δ Decay Rates = ns
p = 0.33
t1/2 = 47d
t1/2 = 187d
Months
D
AIM+ CD8 T (4 of 5 markers)
36/41
27/39
13/31
10/15
3/13
2/9
% of nnCD8+
CD8+ AIM+
Δ Decay Rates = ns
p = 0.11
t1/2 = 27d
t1/2 = NA
SARS-CoV-2 Naive N = 42
SARS-CoV-2 Recovered N = 16
+ mRNA Vaccine
○ Pfizer
△ Moderna
E
CD4+ Memory Subsets
CCR7+: CM
CCR7-: EM1
Naive
CCR7+: EM2
CCR7-: EM3
CCR7-: EMRA
CD27
CD45RA
F
CD4+ Memory Subsets
AIM+ CM
AIM+ EM1
AIM+ EM2
AIM+ EM3
AIM+ EMRA
% of nnCD4+
Naive
Recovered
Months Post–Vaccine
G
Total CD4 Durability (% of Peak Magnitude)
3m
6m
ρ
CM
EM1
EM2
EM3
EMRA
% of Peak Response
P value
* < 0.05
** < 0.01
H
ρ = 0.5 , p = 0.0062
6 Month Durability
% EM1 at 1 Month
I
CD4+ Helper Subsets
cTfh
SSC-A
CXCR5
AIM+ Cells
Th17
Th1/17
Th2
Th1
CCR6
CXCR3
J
CD4+ Helper Subsets
AIM+ cTfh
AIM+ Th1
AIM+ Th2
AIM+ Th17
AIM+ Th1/17
% of nnCD4+
Naive
Recovered
Months Post–Vaccine

Fig. 5. SARS-CoV-2 mRNA vaccines generate durable memory T cell responses. (**A**) Experimental design and (**B**) Gating strategy for quantifying the frequency of SARS-CoV-2-specific CD4+ and CD8+ T cells by AIM assay. For CD4+ T cells, antigen specificity was defined based on co-expression of CD40L and CD200. For CD8+ T cells, antigen specificity was defined based on expression of at least 4/5 activation markers as indicated in A. (**C**) Frequencies of AIM+ CD4+ T and (**D**) AIM+ CD8+ T cells over time in PBMC samples from vaccinated individuals. Data were background subtracted using a paired unstimulated control for each timepoint and are represented as a percentage of non-naïve CD4+ or CD8+ T cells. Black triangles indicate time of vaccine doses, fractions above plots indicate the number of individuals above their individual baseline at memory timepoints, and summary plots show mean values with the 95% confidence interval. Decay rates were calculated using a piecewise linear mixed effects model with censoring. Δ Decay Rates indicates whether decay rates were different in SARS-CoV-2 naïve and recovered groups. (**E**) AIM+ CD4+ T cell memory subsets were identified based on surface expression of CD45RA, CD27, and CCR7. (**F**) Frequencies of AIM+ CD4+ T cell memory subsets over time. (**G**) Correlation matrix of memory subset skewing at peak (1 month) response with total AIM+ CD4+ T cell durability at 3 and 6 months. Durability was measured as the percent of peak response maintained at memory timepoints for each individual. (**H**) Correlation between percent of EM1 cells at peak response and 6-month durability. (**I**) AIM+ CD4+ T helper subsets were defined based on chemokine receptor expression. (**J**) Frequencies of AIM+ CD4+ T helper subsets over time. For F and J, lines connect mean values at different timepoints. Dotted lines indicate the limit of detection for the assay. Statistics were calculated using unpaired non-parametric Wilcoxon test with BH correction for multiple comparisons. Correlations were calculated using non-parametric Spearman rank correlation. * = $p < 0.05$, ** = $p < 0.01$, *** = $p < 0.001$, **** = $p < 0.0001$, ns = not significant.

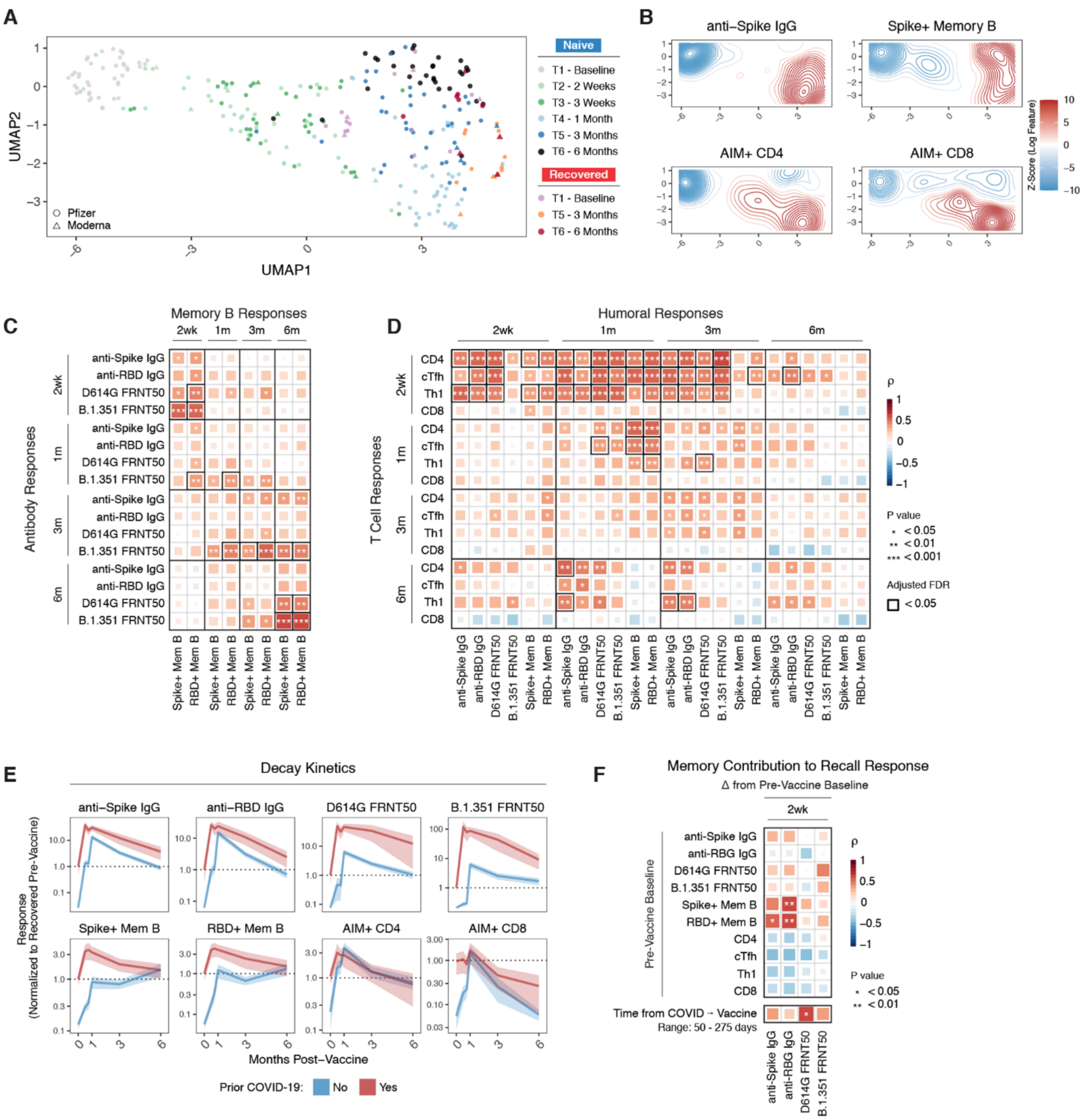

Fig. 6. Immune trajectories and relationships in response to SARS-CoV-2 mRNA vaccination. (**A**) UMAP of 12 antigen-specific parameters of antibody, memory B, and memory T cell responses to mRNA vaccination in SARS-CoV-2 naïve and recovered subjects. Data points represent individual participants and are colored by timepoint relative to primary vaccine. (**B**) Kernel density plots of anti-Spike IgG, Spike+ memory B, AIM CD4+, and AIM+ CD8+ T cells. Red contours represent areas of UMAP space that are enriched for specific immune components. (**C**) Correlation matrix of antibody and memory B cell responses over time in SARS-CoV-2 naïve subjects. (**D**) Correlation matrix of T cell and humoral responses over time in SARS-CoV-2 naïve subjects. (**E**) Decay kinetics of antibody, memory B cell, and memory T cell parameters over time in SARS-CoV-2 naïve and recovered vaccinees. Data are normalized to pre-vaccine levels in SARS-CoV-2 recovered individuals to evaluate the effect of boosting pre-existing immunity. Lines connect mean values at different timepoints, ribbons represent the 95% confidence interval of the mean, and dotted lines indicate mean values at baseline. (**F**) Correlation matrix of baseline memory components and time since infection with antibody recall responses after vaccination in SARS-CoV-2 recovered individuals. Recall responses were calculated as the difference between post-vaccination levels and pre-vaccine baseline. All statistics were calculated using non-parametric Spearman rank correlation.
