# Supplementary material for: mRNA vaccines induce durable immune memory to SARS-CoV-2 and variants of concern
Source: Science. 2021 Oct 14;374(6572):abm0829. doi: 10.1126/science.abm0829 (PMC9284784; doi:10.1126/science.abm0829)
Supplement: Supplementary file 2 — Figs. S1 to S7 Tables S1 to S5 [file science.abm0829_sm.pdf]

Science AAAS

Supplementary Materials for

mRNA vaccines induce durable immune memory to SARS-CoV-2 and variants of concern

Rishi R. Goel *et al.*

Corresponding author: E. John Wherry, wherry@pennmedicine.upenn.edu

Science **374**, eabm0829 (2021)
DOI: 10.1126/science.abm0829

The PDF file includes:

Figs. S1 to S7
Tables S1 to S5

Figure S1

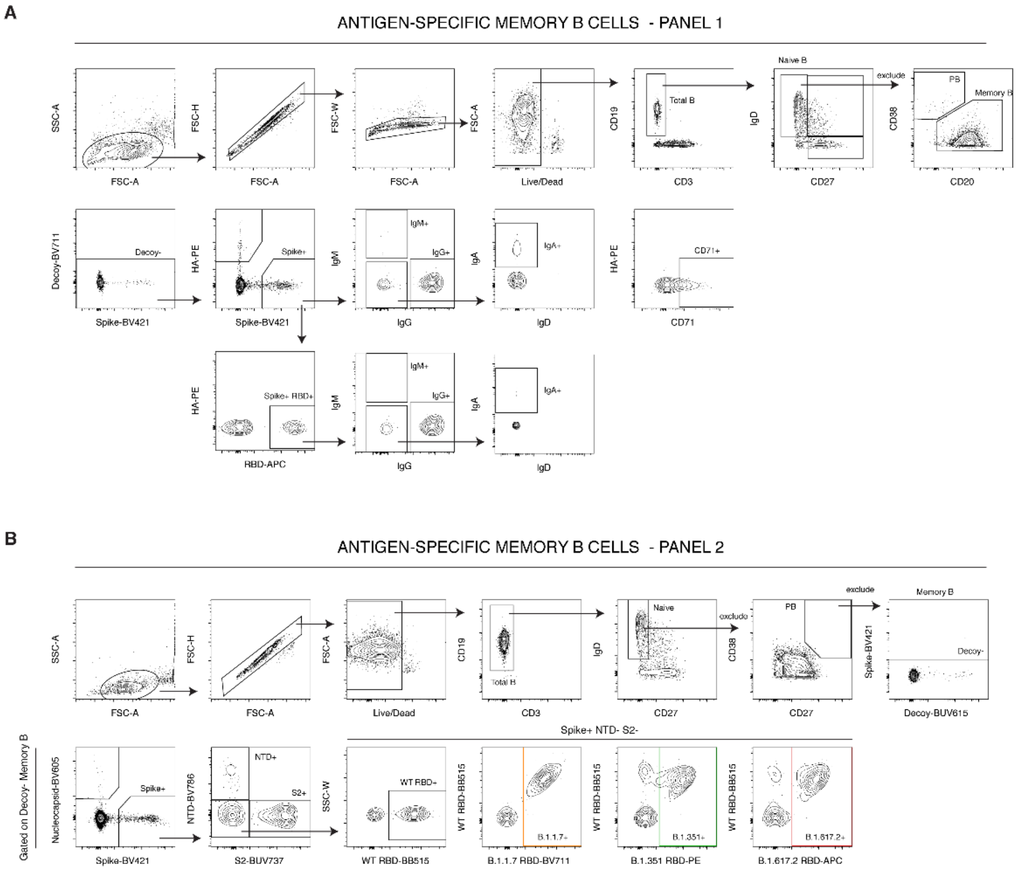

Figure S1. Gating strategy for SARS-CoV-2-specific memory B cells. A) For **panel 1**, lymphocytes were first identified based on forward- and side-scatter from bulk PBMC samples. Singlets were excluded by FSC-A/FSC-H and FSC-A/FSC-W. Dead cells were excluded using Ghost 510 viability dye. Total B cells were then identified as CD3- CD19+ cells. Naïve B cells were identified as IgD+ CD27- B cells and excluded from downstream analysis. Memory B cells were subsequently identified from non-naïve B cells as CD20+ CD38lo/int cells. A BV711 decoy probe was used to gate out memory B cells that non-specifically bound streptavidin. Spike- and HA-binding were then quantified on decoy- memory B cells. Binding to RBD probe was also measured on Spike+ memory B cells. IgG, IgM, and IgA isotypes were evaluated for both Spike+ and Spike+ RBD+ memory B cells. CD71 was measured as an activation marker on Spike+ memory B cells. **B)** For **panel 2**, total B cells were enriched by negative selection from PBMC samples prior to staining. Live, non-naïve B cells were identified as described above. Plasmablasts were then identified as CD27+ CD38+ non-naïve B cells and were excluded from downstream analysis. Decoy- cells were excluded as described above. Spike- and nucleocapsid-specific B cells were identified based on binding to corresponding probes. Spike+ memory cells were then analyzed for co-binding to N-terminal domain (NTD) or S2 domain probes. Memory B cells that were Spike+ but NTD- and S2- were subsequently analyzed for co-binding to a panel of variant RBD probes, including wild-type (WT), B.1.1.7, B.1.351, and B.1.617.2 RBDs. IgG expression was evaluated for all antigen-specific populations.

Figure S2

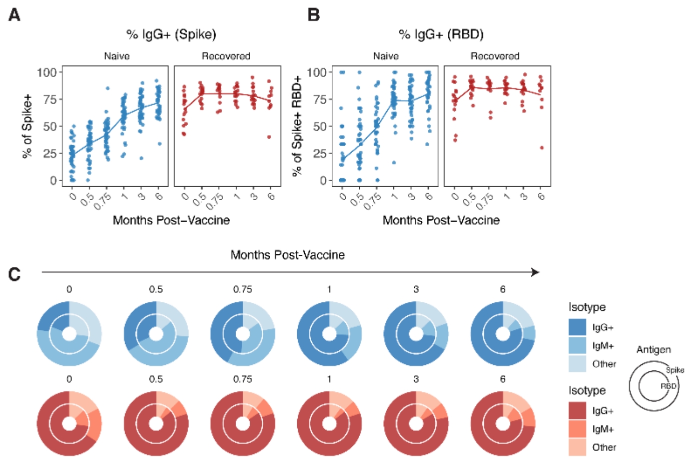

Figure S2. Class-switching of SARS-CoV-2-specific memory B cells after mRNA vaccination. A) Percent IgG+ of Spike+ and **B)** Spike+ RBD+ memory B cells over time after mRNA vaccination. Lines connect mean values at different timepoints. **C)** Summary statistics for % IgG+, % IgM+, and % other isotype+ of SARS-CoV-2-specific memory B cells over time. Outer rings represent total Spike+ memory B cells, inner rings represent Spike+ RBD+ memory B cells.

Figure S3

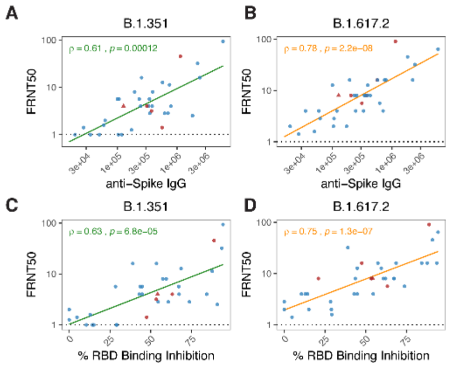

Figure S3. Functional characterization of memory B cell-derived SARS-CoV-2-specific antibodies. A) Correlation of anti-Spike IgG with pseudovirus neutralization titers against B.1.351 and **B)** B.1.617.2 in culture supernatants from *in vitro* stimulation. **C)** Correlation of hACE2-RBD-binding inhibition with pseudovirus neutralization titers against B.1.351 and **D)** B.1.617.2 in culture supernatants from *in vitro* stimulation. Correlations were calculated using non-parametric Spearman rank correlation.

Figure S4

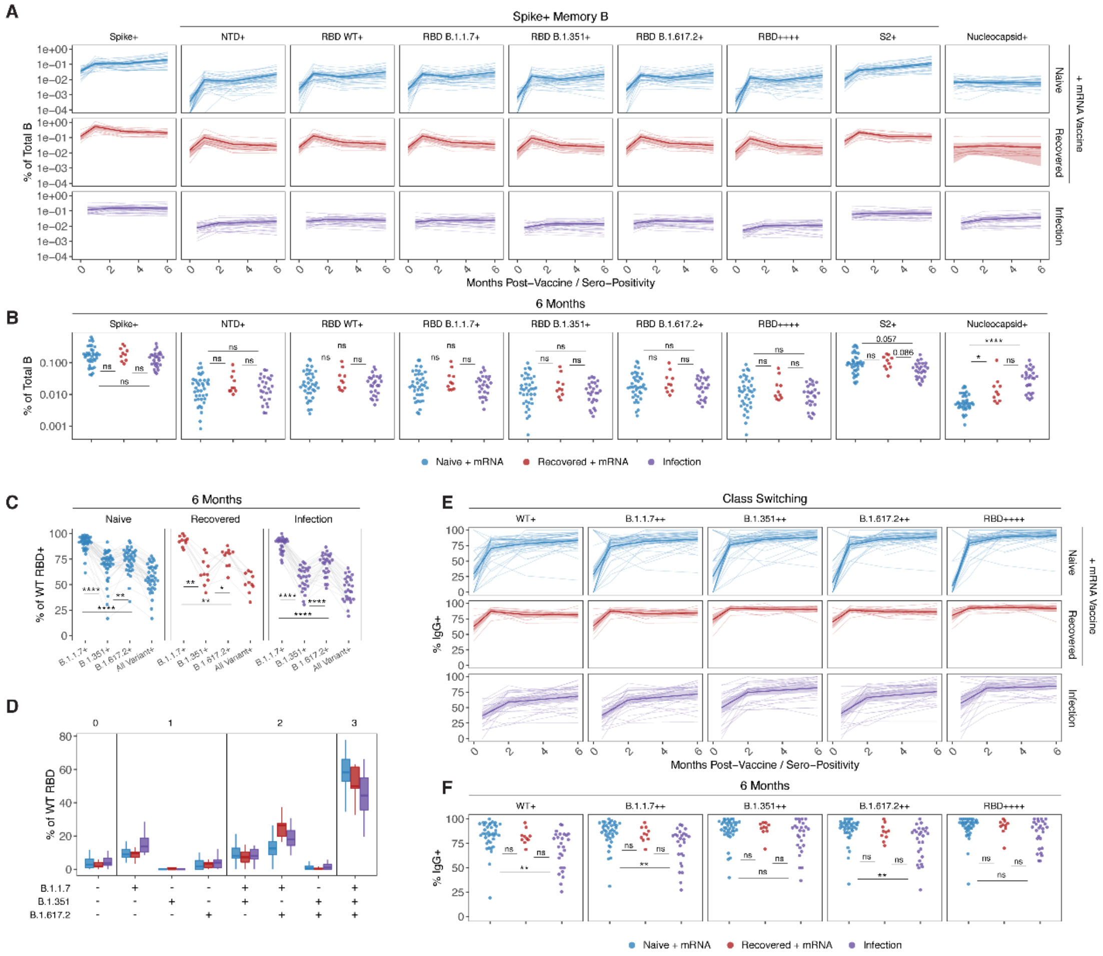

Figure S4. SARS-CoV-2 variant-specific memory B cell frequencies and class-switching after mRNA vaccination or infection. A) Frequency of SARS-CoV-2 antigen-specific memory B cells over time after mRNA vaccination or infection. Data are represented as a percentage of total B cells. **B)** Cross sectional analysis of antigen-specific memory B cell frequencies at 6 months post-vaccination or sero-positivity. **C)** Paired analysis of variant cross-binding in WT RBD+ memory B cells at 6 months post-vaccination or sero-positivity. **D)** Frequency of WT RBD+ memory B cells with different variant cross-binding phenotypes at 6 months post-vaccination or sero-positivity. **E)** Class-switching of RBD-binding cells over time after mRNA vaccination or infection. Data are represented as the percent of cells that are IgG+. **F)** Cross sectional analysis of class-switching to IgG in RBD-binding memory B cells at 6 months post-vaccination or sero-positivity. Statistics were calculated using unpaired (**B** and **F**) or paired (**C**) non-parametric Wilcoxon test with BH correction for multiple comparisons. * = $p < 0.05$, ** = $p < 0.01$, *** = $p < 0.001$, **** = $p < 0.0001$, ns = not significant.

Figure S5

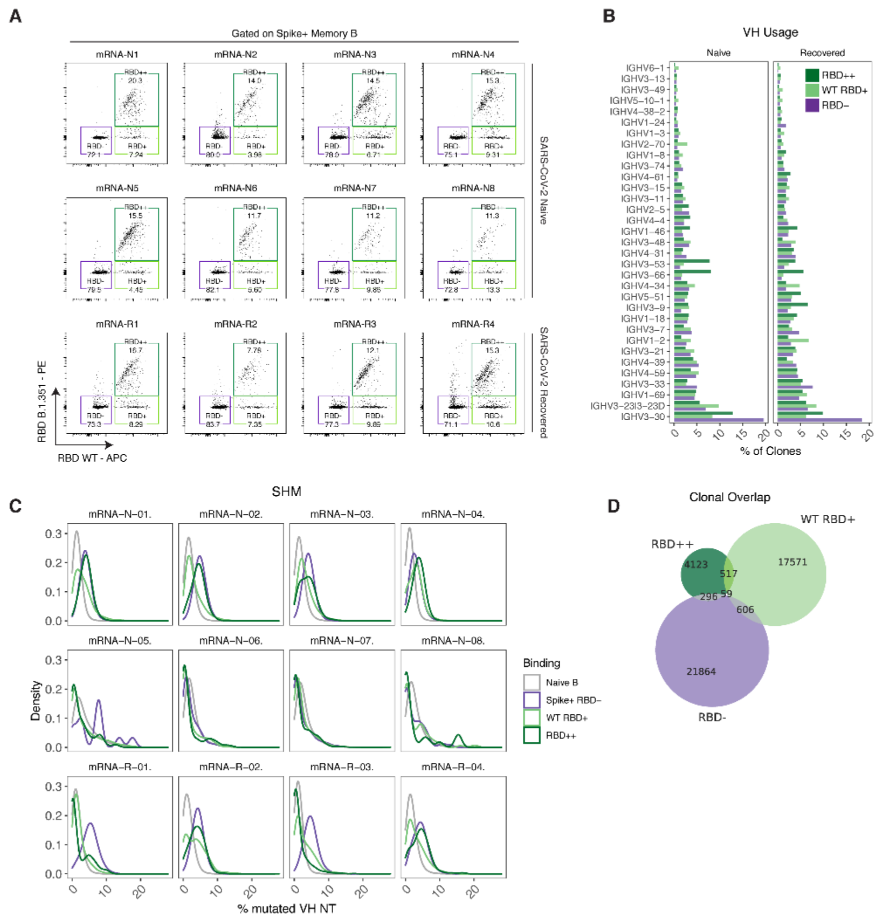

Figure S5. Extended analysis of SARS-CoV-2-specific antibody sequences. A) Individual flow plots of sorted memory B cell populations in 8 SARS-CoV-2 naïve and 4 SARS-CoV-2 recovered individuals. **B)** VH gene usage across different antigen binding populations. Data are represented as a percentage of the overall clones for a given antigen-binding population. **C)** Individual somatic hypermutation (SHM) distributions of memory B cell clones for SARS-CoV-2 naïve and recovered subjects. Data are represented as the percent of mutated VH gene nucleotides. **D)** Venn diagram of clonal overlap between RBD-, WT RBD+ and RBD++ populations. Data were filtered based on larger clones with ≥ 50% mean copy number frequency in each sequencing library.

Figure S6

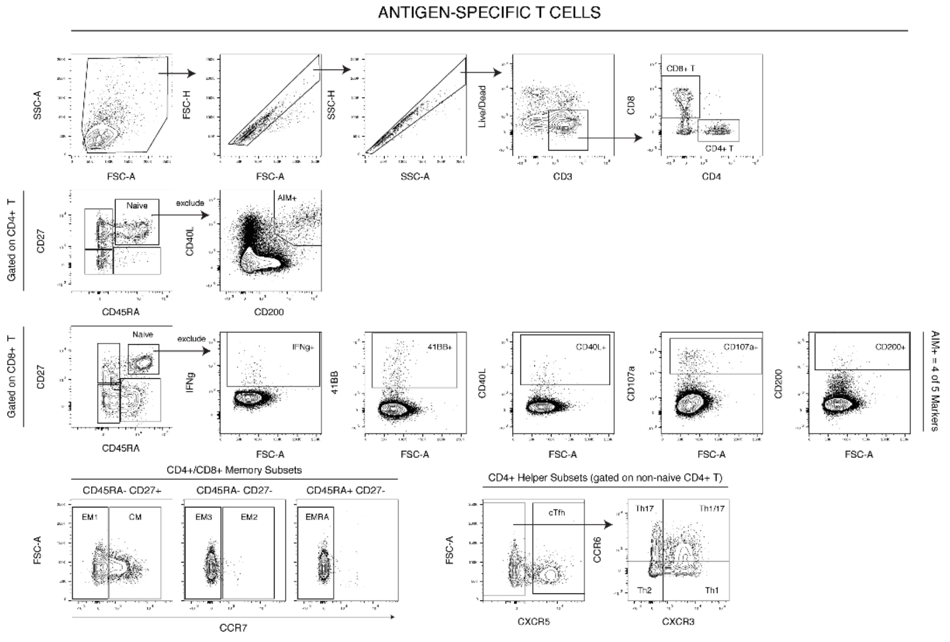

Figure S6. Gating strategy for SARS-CoV-2-specific memory T cells. Lymphocytes were first identified based on forward- and side-scatter from bulk PBMC samples. Singlets were identified by FSC-A/FSC-H and SSC-A/SSC-H. Total T cells were identified as Live/Dead- CD3+. CD4 and CD8 T cells were then identified from total T cells. For both CD4+ and CD8+ T cells, naïve cells were identified as CD45RA+ CD27+ and excluded from downstream analysis. Memory subsets were defined based on a combination of CD45A, CD27, and CCR7 expression. CD4+ helper subsets were defined based on CCR6, CXCR3, and CXCR5 chemokine receptor expression. AIM+ CD4+ T cells were identified based on co-expression of CD40L and CD200. AIM+ CD8+ T cells were identified based on co-expression of at least 4 of 5 activation induced markers (intracellular IFN-g, 41BB, CD40L, CD107a, CD200).

Figure S7

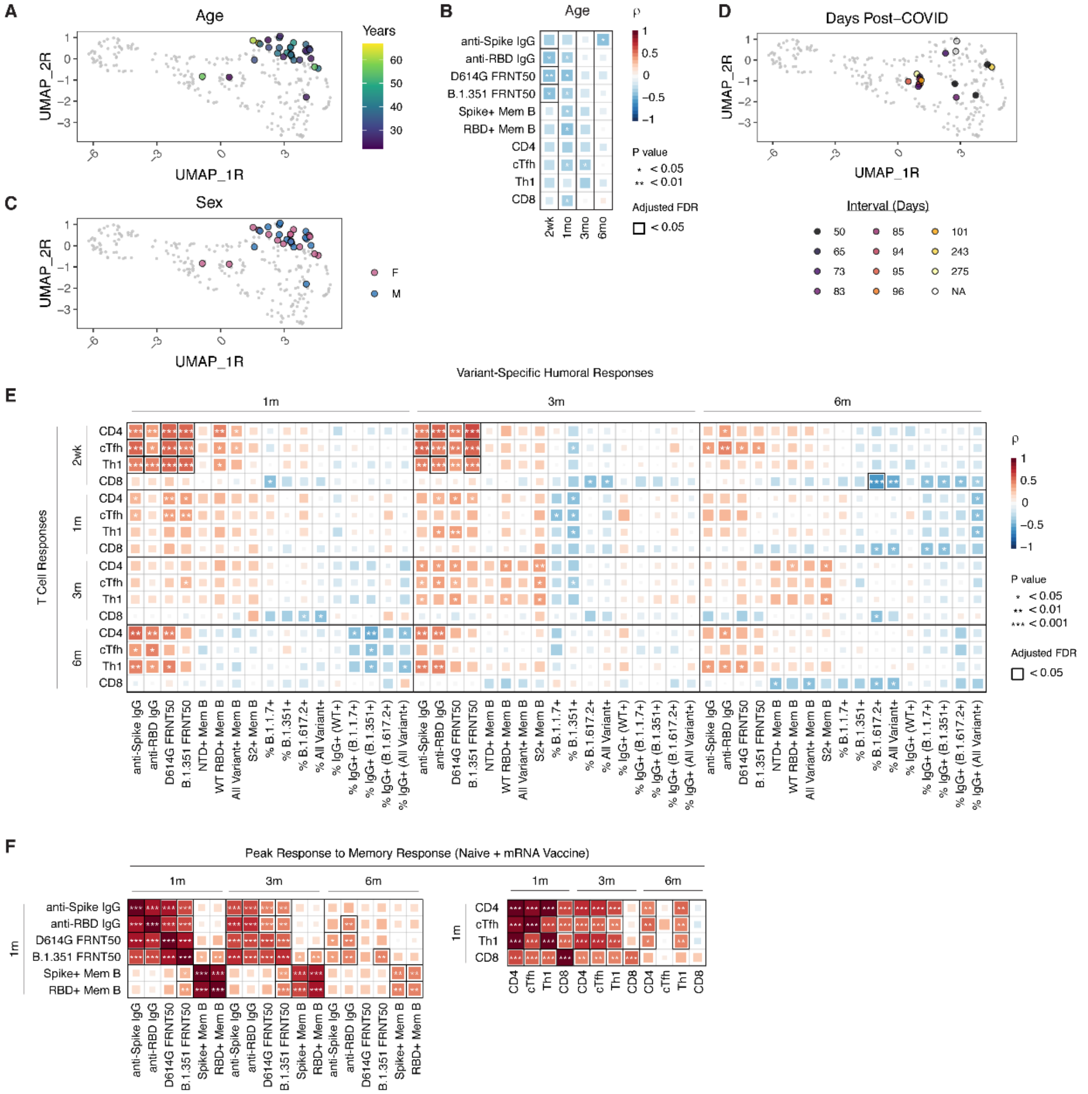

Figure S7. Immune correlations after mRNA vaccination. A) Relationship between age and overall vaccine response in SARS-CoV-2 naïve subjects. 6-month post-vaccination samples are colored by age and projected onto the UMAP coordinates from figure 6. **B)** Correlation between individual immune parameters and age over time after vaccination in SARS-CoV-2 naïve subjects. **C)** Relationship between sex and overall vaccine response in SARS-CoV-2 naïve subjects. 6-month post-vaccination samples are colored by sex and projected onto the UMAP coordinates from figure 6. **D)** Relationship between time since infection and pre-vaccine immune phenotype in SARS-CoV-2 recovered subjects. Pre-vaccine samples are colored based on days since infection and projected onto the UMAP coordinates from figure 6. **E)** Correlation between T cell responses and variant-specific humoral responses over time in SARS-CoV-2 naïve subjects. **F)** Correlation between peak antibody, memory B, and memory T cell responses 1 week after the second vaccine dose with later responses at 3- and 6-months post-vaccination in SARS-CoV-2 naïve subjects. All statistics were calculated using non-parametric Spearman rank correlation.

		SARS-CoV-2 Naïve	SARS-CoV-2 Recovered	Infection Only
**Total**	Number	45	16	26
**Age**	Average (Years)	36.9	38.3	35.5
	Range (Years)	22-67	23-59	25-58
	20-30	15	4	10
	30-40	14	6	9
	40-50	9	2	4
	50+	7	4	3
**Sex**	Male	21	10	9
	Female	24	6	17
**Race/Ethnicity**	White - Non-Hispanic/Latino	27	7	23
	White - Hispanic/Latino	4	1	0
	Asian	8	6	1
	Black	3	1	2
	Native	0	1	0
	Mixed	2	0	0
	Other	1	0	0
**Vaccine Type**	Pfizer	42	12	--
	Moderna	3	4	--
**Time Between Infection and Vaccine**	Average (Days)	--	102.4	--
	Range (Days)	--	50-275; 2 N/A	--

Table S1. Demographic Information for University of Pennsylvania Healthy COVID Vaccine and Healthcare Worker (HCW) Sero-Monitoring Studies. Number of participants, age, sex, race/ethnicity, and vaccine type are indicated. For SARS-CoV-2 recovered vaccinees, time between infection and vaccine is also indicated.

Sample ID	Recovered	Population	Input DNA (ng)	Copies	Unique	# of 2-Copy Productive Clones	Average VH Identity	CDR3 Length (NT)
N1	No	Naïve B	200	122567	98404	11990	0.98	53.84
N1	No	Spike+ RBD-	35.88	148254	118969	5701	0.96	51.01
N1	No	Spike+ WT RBD+	9.516	211511	108374	1905	0.97	49.84
N1	No	Spike+ RBD++	3.614	185656	93739	1458	0.96	49.28
N2	No	Naïve B	200	137926	116872	15574	0.98	54.91
N2	No	Spike+ RBD-	46.8	126678	106225	6081	0.95	54.54
N2	No	Spike+ WT RBD+	21.19	161592	105657	3703	0.97	54.43
N2	No	Spike+ RBD++	5.746	172531	89797	1395	0.96	51.98
N3	No	Naïve B	200	144164	114631	12975	0.98	54.20
N3	No	Spike+ RBD-	20.67	142926	93621	5040	0.96	51.39
N3	No	Spike+ WT RBD+	29.9	152730	112390	5439	0.97	53.33
N3	No	Spike+ RBD++	3.692	156165	78526	1131	0.96	50.55
N4	No	Naïve B	200	171525	130359	12634	0.99	55.88
N4	No	Spike+ RBD-	46.8	178318	129650	4218	0.97	53.15
N4	No	Spike+ WT RBD+	N.D	162184	28112	215	0.97	51.56
N4	No	Spike+ RBD++	3.952	215612	87554	899	0.96	49.92
N5	No	Naïve B	200	132781	102777	17464	0.96	53.05
N5	No	Spike+ RBD-	N.D	6786	1397	12	0.93	57.00
N5	No	Spike+ WT RBD+	5.356	111474	44545	979	0.96	53.13
N5	No	Spike+ RBD++	N.D	135740	18454	139	0.97	49.47
N6	No	Naïve B	200	180913	145385	16281	0.97	52.97
N6	No	Spike+ RBD-	4.914	157476	73933	1336	0.98	51.79
N6	No	Spike+ WT RBD+	4.628	214823	75025	1016	0.98	50.56
N6	No	Spike+ RBD++	N.D	123634	32078	313	0.98	50.83
N7	No	Naïve B	200	195059	155054	18795	0.97	52.16
N7	No	Spike+ RBD-	2.392	243454	85933	944	0.98	49.51
N7	No	Spike+ WT RBD+	7.514	243014	118718	1863	0.97	50.98
N7	No	Spike+ RBD++	1.638	265908	61669	557	0.98	47.70
N8	No	Naïve B	127.4	118333	91634	11738	0.97	52.85
N8	No	Spike+ RBD-	N.D	168131	37287	251	0.97	55.02
N8	No	Spike+ WT RBD+	N.D	113831	24573	220	0.97	49.60
N8	No	Spike+ RBD++	N.D	17036	3030	62	0.97	42.92
R1	Yes	Naïve B	200	143463	110315	20150	0.99	54.68
R1	Yes	Spike+ RBD-	22.75	167823	90997	2953	0.95	52.46
R1	Yes	Spike+ WT RBD+	23.92	152168	91549	4541	0.98	55.47
R1	Yes	Spike+ RBD++	N.D	154271	30773	237	0.98	52.24
R2	Yes	Naïve B	200	130861	118251	40083	0.99	52.92
R2	Yes	Spike+ RBD-	19.63	140803	90062	3648	0.96	52.23
R2	Yes	Spike+ WT RBD+	2.964	219907	75961	872	0.96	53.12
R2	Yes	Spike+ RBD++	N.D	234281	58686	599	0.96	52.52
R3	Yes	Naïve B	200	210839	164405	26661	0.99	55.22
R3	Yes	Spike+ RBD-	22.49	122283	76751	3321	0.95	50.32
R3	Yes	Spike+ WT RBD+	7.046	239056	105423	1738	0.97	54.58
R3	Yes	Spike+ RBD++	N.D	202133	49395	418	0.99	52.24
R4	Yes	Naïve B	200	167836	134501	19019	0.98	54.34
R4	Yes	Spike+ RBD-	23.01	176440	108360	3355	0.96	52.49
R4	Yes	Spike+ WT RBD+	13.65	187951	94348	2940	0.97	52.69
R4	Yes	Spike+ RBD++	5.512	147560	53828	893	0.96	51.36

Table S2. BCR Sequencing Metadata. Sample ID indicates subject; Recovered indicates prior COVID-19. Two independent PCR amplifications (biological replicates) were performed for each sample; Total input DNA from 2 replicates; Number of valid sequence copies (passing length and other QC filters, see methods); Clones are defined as sequences that share the same VH, JH, CDR3 length and are at least 85% identical in the third complementarity determining region (CDR3) amino acid sequence; Productive rearrangements only. Clones with only 1 copy at the subject level are excluded; Average VH identity compared to the nearest germline VH gene (average identity was calculated for each clone and then averaged across clones with each clone counted once per sample); CDR3 length in nucleotides (nt).

	Fold Change at 3 Months		Decay (3-6 Months)	Estimated Duration of Boosting (Days from Vaccine)	
	**Pre-Boost Baseline**	**Naïve Peak Response**	**Half-Life (Days)**	**Pre-Boost Baseline**	**Naïve Peak Response**
**anti-Spike IgG**	12.3 (7.0 - 17.7)	0.92 (0.52 - 1.32)	48	264 (224 - 289)	84 (45 - 109)
**anti-RBD IgG**	10.7 (6.0 - 15.4)	0.72 (0.41 - 1.04)	47	251 (211 - 275)	68 (29 - 93)
**D614G FRNT50**	33.9 (14.6 - 53.3)	5.43 (2.33 - 8.53)	72	456 (368 - 503)	266 (178 - 313)
**B.1.351 FRNT50**	43.7 (17.4 - 70.1)	7.10 (2.83 - 11.38)	63	433 (350 - 476)	268 (185 - 311)

Table S3: Estimated Duration of Vaccine-Boosted Antibody Responses in SARS-CoV-2 Recovered Individuals. Data are presented as mean values with 95% confidence intervals in brackets. Fold change was calculated by dividing antibody levels at 3 months post-vaccination in SARS-CoV-2 recovered individuals by baseline (pre-vaccine) levels, or levels at 1-month post-vaccine in SARS-CoV-2 naïve individuals (naïve peak response). To estimate the duration of boosting (i.e., time to return to pre-vaccine baseline or naïve peak response levels), the number of times the fold change could be halved before reaching a value of 1 was multiplied by the mean calculated population half-lives for decay from 3-6 months.

Reagent	Vendor	Identifier	Concentration
**Panel 1 - B Cell Probe**			
SARS-CoV-2 Biotinylated Full Length Spike	R&D Systems	BT10549-050	200ng
SARS-CoV-2 Biotinylated Full Length Spike	R&D Systems	BT10500-050	25ng
HA(ΔTM)(A/Brisbane/02/2018)(H1N1)	Immune Tech	IT-003-00110ΔTMp	50ng
HA(ΔTM)(B/Colorado/06/2017)	Immune Tech	IT-003-B21ΔTMp	50ng
BV421 Streptavidin	Biolegend	405226	20ng
BV711 Streptavidin	BD Biosciences	563262	20ng
PE Streptavidin	Biolegend	405203	16ng
APC Streptavidin	Biolegend	405207	12.5ng
Ghost Viability Dye Violet 510	Tonbo	13-0870-T100	1:600
BUV563 anti-CD3	BD Biosciences	748569	1:200
BV750 anti-CD19	Biolegend	302262	1:100
BUV805 anti-CD20	BD Biosciences	612905	1:500
BUV395 anti-CD27	BD Biosciences	563815	1:200
BUV661 anti-CD38	BD Biosciences	612969	1:200
APC-H7 anti-CD71	BD Biosciences	563671	1:50
FITC anti-IgA	Miltenyi	130-113-475	1:400
BV480 anti-IgD	BD Biosciences	566138	1:50
PE-Cy7 anti-IgG	Biolegend	410722	1:400
PerCP/Cy5.5 anti-IgM	Biolegend	314512	1:400
**Panel 2 - Variant B Cell Probe**			
SARS-CoV-2 Biotinylated Full Length Spike	R&D Systems	AVI10549-050	200ng
SARS-CoV-2 Biotinylated RBD	Acro Biosystems	SPD-C82E9-25ug	25ng
SARS-CoV-2 Biotinylated RBD (N501Y)	Acro Biosystems	SPD-C82E6-25ug	25ng
SARS-CoV-2 Biotinylated RBD (K417N/E484K/N501Y)	Acro Biosystems	SPD-C82E5-25ug	25ng
SARS-CoV-2 Biotinylated RBD (L452R/K478N)	Acro Biosystems	SPD-C82Ed-25ug	25ng
SARS-CoV-2 Biotinylated N-Terminal Domain	Sino Biological	40591-V49H-B	30ng
SARS-CoV-2 Biotinylated S2	Acro Biosystems	S2N-C52E8-25ug	50ng
SARS-CoV-2 Biotinylated Nucleocapsid	R&D Systems	BT10474-050	50ng
BV421 Streptavidin	Biolegend	405226	20ng
BV605 Streptavidin	Biolegend	405229	14ng
BV711 Streptavidin	BD Biosciences	563262	12.5ng
BV786 Streptavidin	BD Biosciences	563858	12ng
BUV615 Streptavidin	BD Biosciences	613013	12.5ng
BUV737 Streptavidin	BD Biosciences	612775	12ng
BB515 Streptavidin	BD Biosciences	564453	12.5ng
PE Streptavidin	Biolegend	405203	12.5ng
APC Streptavidin	Biolegend	405207	12.5ng
Ghost Viability Dye Violet 510	Tonbo	13-0870-T100	1:600
BUV563 anti-CD3	BD Biosciences	748569	1:200
BV750 anti-CD19	Biolegend	302262	1:100
BUV395 anti-CD27	BD Biosciences	563815	1:200
BUV661 anti-CD38	BD Biosciences	612969	1:200
BV480 anti-IgD	BD Biosciences	566138	1:50
APC-H7 anti-IgG	BD Biosciences	561297	1:100
**Panel 3 - Variant-Specific B Cell Sorting**			
SARS-CoV-2 Biotinylated Full Length Spike	R&D Systems	BT10549-050	200ng
SARS-CoV-2 Biotinylated RBD	Sino Biological	40592-V08B-B	25ng

SARS-CoV-2 Biotinylated RBD (K417N/E484K/N501Y)	Sino Biological	40592-V08H85-B	25ng
BV421 Streptavidin	Biolegend	405226	20ng
AF488 Streptavidin	Biolegend	405235	20ng
PE Streptavidin	Biolegend	405203	12.5ng
APC Streptavidin	Biolegend	405207	12.5ng
Ghost Viability Dye Violet 510	Tonbo	13-0870-T100	1:600
APC-Cy7 anti-CD19	BD Biosciences	557791	1:200
BV650 anti-CD20	Biolegend	302336	1:200
BV785 anti-CD27	Biolegend	302832	1:66
PE-Cy7 anti-CD38	eBioscience	25-0389-42	1:200
PE-CF594 anti-IgD	BD Biosciences	562540	1:50

Table S4. Reagents for Memory B Cell Analysis. Reagent, vendor, catalog number, and concentration/dilution are indicated.

Reagent	Vendor	Identifier
**Flow Cytometry Antibodies**		
BUV395 CD4	BD Biosciences	Cat#563550
BUV496 CD8	BD Biosciences	Cat#612943
BUV615 CD45RA	BD Biosciences	Cat#751555
BUV737 CD27	BD Biosciences	Cat#612829
BUV805 CD3	BD Biosciences	Cat#612896
BV421 CXCR3	Biolegend	Cat#353716
BV650 CCR7	Biolegend	Cat#353234
BV605 CD69	Biolegend	Cat#310938
BV711 CD40L	Biolegend	Cat#310838
BV785 CD107a	Biolegend	Cat#328644
FITC IFNy	Biolegend	Cat#502515
PE CD200	Biolegend	Cat#399804
PE-Cy7 OX40	Biolegend	Cat#350012
AF647 41BB	Biolegend	Cat#309810
APC-R700 CXCR5	BD Biosciences	Cat#565191
APC-Cy7 CCR6	Biolegend	Cat#353432
**Peptides**		
CD4-S peptide Megapool	Synthetic Biomolecules (aka A&A)	http://www.syntheticbiomolecules.com/
CD8-E peptide Megapool	Synthetic Biomolecules (aka A&A)	http://www.syntheticbiomolecules.com/
**Other**		
Ghost Dye Violet 510	Tonbo	Cat#13-0870-T500
GolgiStop (Containing Monensin)	BD Biosciences	Cat#51-2092K7
CD40 Antibody, anti-human, pure-functional grade	Miltenyi Biotech	Cat#130-094-133
Anti-Human CD28/CD49d Purified	BD Biosciences	Cat#347690
Human TruStain FcX™ (Fc Receptor Blocking Solution)	Biolegend	Cat#422302
Foxp3 / Transcription Factor Fixation/Permeabilization Concentrate and Diluent	eBioscience	Cat#00-5521-00

Table S5. Reagents for Memory T Cell Analysis. Reagent, vendor, and catalog number are indicated.

References and Notes

1. T. Carvalho, F. Krammer, A. Iwasaki, The first 12 months of COVID-19: A timeline of immunological insights. *Nat. Rev. Immunol.* **21**, 245–256 (2021). doi:10.1038/s41577-021-00522-1 Medline

2. F. P. Polack, S. J. Thomas, N. Kitchin, J. Absalon, A. Gurtman, S. Lockhart, J. L. Perez, G. Pérez Marc, E. D. Moreira, C. Zerbini, R. Bailey, K. A. Swanson, S. Roychoudhury, K. Koury, P. Li, W. V. Kalina, D. Cooper, R. W. Frenck, L. L. Hammitt, Ö. Türeci, H. Nell, A. Schaefer, S. Ünal, D. B. Tresnan, S. Mather, P. R. Dormitzer, U. Şahin, K. U. Jansen, W. C. Gruber, Safety and Efficacy of the BNT162b2 mRNA Covid-19 Vaccine. *N. Engl. J. Med.* **383**, 2603–2615 (2020). doi:10.1056/NEJMoa2034577

3. L. R. Baden, H. M. El Sahly, B. Essink, K. Kotloff, S. Frey, R. Novak, D. Diemert, S. A. Spector, N. Rouphael, C. B. Creech, J. McGettigan, S. Khetan, N. Segall, J. Solis, A. Brosz, C. Fierro, H. Schwartz, K. Neuzil, L. Corey, P. Gilbert, H. Janes, D. Follmann, M. Marovich, J. Mascola, L. Polakowski, J. Ledgerwood, B. S. Graham, H. Bennett, R. Pajon, C. Knightly, B. Leav, W. Deng, H. Zhou, S. Han, M. Ivarsson, J. Miller, T. Zaks, Efficacy and Safety of the mRNA-1273 SARS-CoV-2 Vaccine. *N. Engl. J. Med.* **384**, 403–416 (2021). doi:10.1056/NEJMoa2035389

4. D. S. Khoury, D. Cromer, A. Reynaldi, T. E. Schlub, A. K. Wheatley, J. A. Juno, K. Subbarao, S. J. Kent, J. A. Triccas, M. P. Davenport, Neutralizing antibody levels are highly predictive of immune protection from symptomatic SARS-CoV-2 infection. *Nat. Med.* **27**, 1205–1211 (2021). doi:10.1038/s41591-021-01377-8 Medline

5. D. Cromer, M. Steain, A. Reynaldi, T. E. Schlub, A. K. Wheatley, J. A. Juno, S. J. Kent, J. A. Triccas, D. S. Khoury, M. P. Davenport, SARS-CoV-2 variants: levels of neutralisation required for protective immunity. medRxiv 2021.08.11.21261876 [Preprint] (2021). https://doi.org/10.1101/2021.08.11.21261876.

6. P. B. Gilbert, D. C. Montefiori, A. McDermott, Y. Fong, D. Benkeser, W. Deng, H. Zhou, C. R. Houchens, K. Martins, L. Jayashankar, F. Castellino, B. Flach, B. C. Lin, S. O'Connell, C. McDanal, A. Eaton, M. Sarzotti-Kelsoe, Y. Lu, C. Yu, B. Borate, L. W. P. van der Laan, N. Hejazi, C. Huynh, J. Miller, H. M. El Sahly, L. R. Baden, M. Baron, L. De La Cruz, C. Gay, S. Kalams, C. F. Kelley, M. Kutner, M. P. Andrasik, J. G. Kublin, L. Corey, K. M. Neuzil, L. N. Carpp, R. Pajon, D. Follmann, R. O. Donis, R. A. Koup, Immune Correlates Analysis of the mRNA-1273 COVID-19 Vaccine Efficacy Trial. medRxiv 2021.08.09.21261290 [Preprint] (2021). https://doi.org/10.1101/2021.08.09.21261290.

7. N. Doria-Rose, M. S. Suthar, M. Makowski, S. O'Connell, A. B. McDermott, B. Flach, J. E. Ledgerwood, J. R. Mascola, B. S. Graham, B. C. Lin, S. O'Dell, S. D. Schmidt, A. T. Widge, V.-V. Edara, E. J. Anderson, L. Lai, K. Floyd, N. G. Rouphael, V. Zarnitsyna, P. C. Roberts, M. Makhene, W. Buchanan, C. J. Luke, J. H. Beigel, L. A. Jackson, K. M. Neuzil, H. Bennett, B. Leav, J. Albert, P. Kunwar, mRNA-1273 Study Group, Antibody Persistence through 6 Months after the Second Dose of mRNA-1273 Vaccine for Covid-19. *N. Engl. J. Med.* **384**, 2259–2261 (2021). doi:10.1056/NEJMc2103916 Medline

8. M. Bergwerk, T. Gonen, Y. Lustig, S. Amit, M. Lipsitch, C. Cohen, M. Mandelboim, E. G. Levin, C. Rubin, V. Indenbaum, I. Tal, M. Zavitan, N. Zuckerman, A. Bar-Chaim, Y.

Kreiss, G. Regev-Yochay, Covid-19 Breakthrough Infections in Vaccinated Health Care Workers. *N. Engl. J. Med.* 10.1056/NEJMoa2109072 (2021). doi:10.1056/NEJMoa2109072

9. A. Israel, E. Merzon, A. A. Schäffer, Y. Shenhar, I. Green, A. Golan-Cohen, E. Ruppin, E. Magen, S. Vinker, Elapsed time since BNT162b2 vaccine and risk of SARS-CoV-2 infection in a large cohort. medRxiv 2021.08.03.21261496 [Preprint] (2021). https://doi.org/10.1101/2021.08.03.21261496.

10. S. Y. Tartof, J. M. Slezak, H. Fischer, V. Hong, B. K. Ackerson, O. N. Ranasinghe, T. B. Frankland, O. A. Ogun, J. M. Zamparo, S. Gray, S. R. Valluri, K. Pan, F. J. Angulo, L. Jodar, J. M. McLaughlin, Effectiveness of mRNA BNT162b2 COVID-19 vaccine up to 6 months in a large integrated health system in the USA: A retrospective cohort study. *Lancet* 10.1016/S0140-6736(21)02183-8 (2021). doi:10.1016/S0140-6736(21)02183-8 Medline

11. J. B. Griffin, M. Haddix, P. Danza, R. Fisher, T. H. Koo, E. Traub, P. Gounder, C. Jarashow, S. Balter, SARS-CoV-2 Infections and Hospitalizations Among Persons Aged ≥16 Years, by Vaccination Status — Los Angeles County, California, May 1–July 25, 2021. *MMWR Morb. Mortal. Wkly. Rep.* **70**, 1170–1176 (2021). doi:10.15585/mmwr.mm7034e5

12. S. J. Thomas, E. D. Moreira Jr., N. Kitchin, J. Absalon, A. Gurtman, S. Lockhart, J. L. Perez, G. Pérez Marc, F. P. Polack, C. Zerbini, R. Bailey, K. A. Swanson, X. Xu, S. Roychoudhury, K. Koury, S. Bouguermouh, W. V. Kalina, D. Cooper, R. W. Frenck Jr., L. L. Hammitt, Ö. Türeci, H. Nell, A. Schaefer, S. Ünal, Q. Yang, P. Liberator, D. B. Tresnan, S. Mather, P. R. Dormitzer, U. Şahin, W. C. Gruber, K. U. Jansen, C4591001 Clinical Trial Group, Six Month Safety and Efficacy of the BNT162b2 mRNA COVID-19 Vaccine. medRxiv 2021.07.28.21261159 [Preprint] (2021). https://doi.org/10.1101/2021.07.28.21261159.

13. P. S. Arunachalam, M. K. D. Scott, T. Hagan, C. Li, Y. Feng, F. Wimmers, L. Grigoryan, M. Trisal, V. V. Edara, L. Lai, S. E. Chang, A. Feng, S. Dhingra, M. Shah, A. S. Lee, S. Chinthrajah, S. B. Sindher, V. Mallajosyula, F. Gao, N. Sigal, S. Kowli, S. Gupta, K. Pellegrini, G. Tharp, S. Maysel-Auslender, S. Hamilton, H. Aoued, K. Hrusovsky, M. Roskey, S. E. Bosinger, H. T. Maecker, S. D. Boyd, M. M. Davis, P. J. Utz, M. S. Suthar, P. Khatri, K. C. Nadeau, B. Pulendran, Systems vaccinology of the BNT162b2 mRNA vaccine in humans. *Nature* **596**, 410–416 (2021). doi:10.1038/s41586-021-03791-x Medline

14. J. S. Turner, J. A. O'Halloran, E. Kalaidina, W. Kim, A. J. Schmitz, J. Q. Zhou, T. Lei, M. Thapa, R. E. Chen, J. B. Case, F. Amanat, A. M. Rauseo, A. Haile, X. Xie, M. K. Klebert, T. Suessen, W. D. Middleton, P.-Y. Shi, F. Krammer, S. A. Teefey, M. S. Diamond, R. M. Presti, A. H. Ellebedy, SARS-CoV-2 mRNA vaccines induce persistent human germinal centre responses. *Nature* **596**, 109–113 (2021). doi:10.1038/s41586-021-03738-2 Medline

15. K. Lederer, K. Parvathaneni, M. M. Painter, E. Bettini, D. Agarwal, K. A. Lundgreen, M. Weirick, R. R. Goel, X. Xu, E. M. Drapeau, S. Gouma, A. R. Greenplate, C. Le Coz, N. Romberg, L. Jones, M. Rosen, B. Besharatian, M. Kaminiski, D. Weiskopf, A. Sette, S. E. Hensley, P. Bates, E. J. Wherry, A. Naji, V. Bhoj, M. Locci, Germinal center

responses to SARS-CoV-2 mRNA vaccines in healthy and immunocompromised individuals. medRxiv 2021.09.16.21263686 [Preprint] (2021). https://doi.org/10.1101/2021.09.16.21263686.

16. R. R. Goel, S. A. Apostolidis, M. M. Painter, D. Mathew, A. Pattekar, O. Kuthuru, S. Gouma, P. Hicks, W. Meng, A. M. Rosenfeld, S. Dysinger, K. A. Lundgreen, L. Kuri-Cervantes, S. Adamski, A. Hicks, S. Korte, D. A. Oldridge, A. E. Baxter, J. R. Giles, M. E. Weirick, C. M. McAllister, J. Dougherty, S. Long, K. D'Andrea, J. T. Hamilton, M. R. Betts, E. T. Luning Prak, P. Bates, S. E. Hensley, A. R. Greenplate, E. J. Wherry, Distinct antibody and memory B cell responses in SARS-CoV-2 naïve and recovered individuals after mRNA vaccination. *Sci. Immunol.* **6**, eabi6950 (2021). doi:10.1126/sciimmunol.abi6950 Medline

17. A. Cho, F. Muecksch, D. Schaefer-Babajew, Z. Wang, S. Finkin, C. Gaebler, V. Ramos, M. Cipolla, P. Mendoza, M. Agudelo, E. Bednarski, J. DaSilva, I. Shimeliovich, J. Dizon, M. Daga, K. Millard, M. Turroja, F. Schmidt, F. Zhang, T. B. Tanfous, M. Jankovic, T. Y. Oliveria, A. Gazumyan, M. Caskey, P. D. Bieniasz, T. Hatziioannou, M. C. Nussenzweig, Anti- SARS-CoV-2 Receptor Binding Domain Antibody Evolution after mRNA Vaccination. bioRxiv 2021.07.29.454333 [Preprint] (2021). https://doi.org/10.1101/2021.07.29.454333.

18. A. Mazzoni, N. Di Lauria, L. Maggi, L. Salvati, A. Vanni, M. Capone, G. Lamacchia, E. Mantengoli, M. Spinicci, L. Zammarchi, S. T. Kiros, A. Rocca, F. Lagi, M. G. Colao, P. Parronchi, C. Scaletti, L. Turco, F. Liotta, G. M. Rossolini, L. Cosmi, A. Bartoloni, F. Annunziato, COVID-19 Research Group, First-dose mRNA vaccination is sufficient to reactivate immunological memory to SARS-CoV-2 in subjects who have recovered from COVID-19. *J. Clin. Invest.* **131**, e149150 (2021). doi:10.1172/JCI149150 Medline

19. M. M. Painter, D. Mathew, R. R. Goel, S. A. Apostolidis, A. Pattekar, O. Kuthuru, A. E. Baxter, R. S. Herati, D. A. Oldridge, S. Gouma, P. Hicks, S. Dysinger, K. A. Lundgreen, L. Kuri-Cervantes, S. Adamski, A. Hicks, S. Korte, J. R. Giles, M. E. Weirick, C. M. McAllister, J. Dougherty, S. Long, K. D'Andrea, J. T. Hamilton, M. R. Betts, P. Bates, S. E. Hensley, A. Grifoni, D. Weiskopf, A. Sette, A. R. Greenplate, E. J. Wherry, Rapid induction of antigen-specific $CD4^+$ T cells is associated with coordinated humoral and cellular immunity to SARS-CoV-2 mRNA vaccination. *Immunity* **54**, 2133–2142.e3 (2021). doi:10.1016/j.immuni.2021.08.001 Medline

20. V. Oberhardt, H. Luxenburger, J. Kemming, I. Schulien, K. Ciminski, S. Giese, B. Csernalabics, J. Lang-Meli, I. Janowska, J. Staniek, K. Wild, K. Basho, M. S. Marinescu, J. Fuchs, F. Topfstedt, A. Janda, O. Sogukpinar, H. Hilger, K. Stete, F. Emmerich, B. Bengsch, C. F. Waller, S. Rieg, Sagar, T. Boettler, K. Zoldan, G. Kochs, M. Schwemmle, M. Rizzi, R. Thimme, C. Neumann-Haefelin, M. Hofmann, Rapid and stable mobilization of $CD8^+$ T cells by SARS-CoV-2 mRNA vaccine. *Nature* **597**, 268–273 (2021). doi:10.1038/s41586-021-03841-4 Medline

21. A. Tarke, J. Sidney, N. Methot, E. D. Yu, Y. Zhang, J. M. Dan, B. Goodwin, P. Rubiro, A. Sutherland, E. Wang, A. Frazier, S. I. Ramirez, S. A. Rawlings, D. M. Smith, R. da Silva Antunes, B. Peters, R. H. Scheuermann, D. Weiskopf, S. Crotty, A. Grifoni, A. Sette, Impact of SARS-CoV-2 variants on the total $CD4^+$ and $CD8^+$ T cell reactivity in infected

or vaccinated individuals. *Cell Rep. Med.* **2**, 100355 (2021). doi:10.1016/j.xcrm.2021.100355

22. J. Mateus, J. M. Dan, Z. Zhang, C. R. Moderbacher, M. Lammers, B. Goodwin, A. Sette, S. Crotty, D. Weiskopf, Low dose mRNA-1273 COVID-19 vaccine generates durable T cell memory and antibodies enhanced by pre-existing crossreactive T cell memory. medRxiv 2021.06.30.21259787 [Preprint] (2021). https://doi.org/10.1101/2021.06.30.21259787.

23. D. Cromer, J. A. Juno, D. Khoury, A. Reynaldi, A. K. Wheatley, S. J. Kent, M. P. Davenport, Prospects for durable immune control of SARS-CoV-2 and prevention of reinfection. *Nat. Rev. Immunol.* **21**, 395–404 (2021). doi:10.1038/s41577-021-00550-x Medline

24. M. Akkaya, K. Kwak, S. K. Pierce, B cell memory: Building two walls of protection against pathogens. *Nat. Rev. Immunol.* **20**, 229–238 (2020). doi:10.1038/s41577-019-0244-2 Medline

25. D. L. Farber, N. A. Yudanin, N. P. Restifo, Human memory T cells: Generation, compartmentalization and homeostasis. *Nat. Rev. Immunol.* **14**, 24–35 (2013). doi:10.1038/nri3567 Medline

26. M. C. Shamier, A. Tostmann, S. Bogers, J. de Wilde, J. IJpelaar, W. A. van der Kleij, H. de Jager, B. Haagmans, R. Molenkamp, B. B. O. Munnink, C. van Rossum, J. Rahamat-Langendoen, N. van der Geest, C. P. Bleeker-Rovers, H. Wertheim, M. P. G. Koopmans, C. H. GeurtsvanKessel, Virological characteristics of SARS-CoV-2 vaccine breakthrough infections in health care workers. medRxiv 2021.08.20.21262158 [Preprint] (2021). https://doi.org/10.1101/2021.08.20.21262158.

27. R. Ke, P. P. Martinez, R. L. Smith, L. L. Gibson, C. J. Achenbach, S. McFall, C. Qi, J. Jacob, E. Dembele, C. Bundy, L. M. Simons, E. A. Ozer, J. F. Hultquist, R. Lorenzo-Redondo, A. K. Opdycke, C. Hawkins, R. L. Murphy, A. Mirza, M. Conte, N. Gallagher, C. H. Luo, J. Jarrett, A. Conte, R. Zhou, M. Farjo, G. Rendon, C. J. Fields, L. Wang, R. Fredrickson, M. E. Baughman, K. K. Chiu, H. Choi, K. R. Scardina, A. N. Owens, J. Broach, B. Barton, P. Lazar, M. L. Robinson, H. H. Mostafa, Y. C. Manabe, A. Pekosz, D. D. McManus, C. B. Brooke, Longitudinal analysis of SARS-CoV-2 vaccine breakthrough infections reveal limited infectious virus shedding and restricted tissue distribution. medRxiv 2021.08.30.21262701 [Preprint] (2021). https://doi.org/10.1101/2021.08.30.21262701.

28. J. M. Dan, J. Mateus, Y. Kato, K. M. Hastie, E. D. Yu, C. E. Faliti, A. Grifoni, S. I. Ramirez, S. Haupt, A. Frazier, C. Nakao, V. Rayaprolu, S. A. Rawlings, B. Peters, F. Krammer, V. Simon, E. O. Saphire, D. M. Smith, D. Weiskopf, A. Sette, S. Crotty, Immunological memory to SARS-CoV-2 assessed for up to 8 months after infection. *Science* **371**, eabf4063 (2021). doi:10.1126/science.abf4063 Medline

29. K. W. Cohen, S. L. Linderman, Z. Moodie, J. Czartoski, L. Lai, G. Mantus, C. Norwood, L. E. Nyhoff, V. V. Edara, K. Floyd, S. C. De Rosa, H. Ahmed, R. Whaley, S. N. Patel, B. Prigmore, M. P. Lemos, C. W. Davis, S. Furth, J. B. O'Keefe, M. P. Gharpure, S. Gunisetty, K. Stephens, R. Antia, V. I. Zarnitsyna, D. S. Stephens, S. Edupuganti, N. Rouphael, E. J. Anderson, A. K. Mehta, J. Wrammert, M. S. Suthar, R. Ahmed, M. J. McElrath, Longitudinal analysis shows durable and broad immune memory after SARS-

CoV-2 infection with persisting antibody responses and memory B and T cells. *Cell Rep. Med.* **2**, 100354 (2021). doi:10.1016/j.xcrm.2021.100354 Medline

30. D. A. Collier, A. De Marco, I. A. T. M. Ferreira, B. Meng, R. P. Datir, A. C. Walls, S. A. Kemp, J. Bassi, D. Pinto, C. Silacci-Fregni, S. Bianchi, M. A. Tortorici, J. Bowen, K. Culap, S. Jaconi, E. Cameroni, G. Snell, M. S. Pizzuto, A. F. Pellanda, C. Garzoni, A. Riva, The CITIID-NIHR BioResource COVID-19 Collaboration, A. Elmer, N. Kingston, B. Graves, L. E. McCoy, K. G. C. Smith, J. R. Bradley, N. Temperton, L. Ceron-Gutierrez, G. Barcenas-Morales, The COVID-19 Genomics UK (COG-UK) Consortium, W. Harvey, H. W. Virgin, A. Lanzavecchia, L. Piccoli, R. Doffinger, M. Wills, D. Veesler, D. Corti, R. K. Gupta, Sensitivity of SARS-CoV-2 B.1.1.7 to mRNA vaccine-elicited antibodies. *Nature* **593**, 136–141 (2021). doi:10.1038/s41586-021-03412-7

31. D. Zhou, W. Dejnirattisai, P. Supasa, C. Liu, A. J. Mentzer, H. M. Ginn, Y. Zhao, H. M. E. Duyvesteyn, A. Tuekprakhon, R. Nutalai, B. Wang, G. C. Paesen, C. Lopez-Camacho, J. Slon-Campos, B. Hallis, N. Coombes, K. Bewley, S. Charlton, T. S. Walter, D. Skelly, S. F. Lumley, C. Dold, R. Levin, T. Dong, A. J. Pollard, J. C. Knight, D. Crook, T. Lambe, E. Clutterbuck, S. Bibi, A. Flaxman, M. Bittaye, S. Belij-Rammerstorfer, S. Gilbert, W. James, M. W. Carroll, P. Klenerman, E. Barnes, S. J. Dunachie, E. E. Fry, J. Mongkolsapaya, J. Ren, D. I. Stuart, G. R. Screaton, Evidence of escape of SARS-CoV-2 variant B.1.351 from natural and vaccine-induced sera. *Cell* **184**, 2348–2361.e6 (2021). doi:10.1016/j.cell.2021.02.037 Medline

32. C. Liu, H. M. Ginn, W. Dejnirattisai, P. Supasa, B. Wang, A. Tuekprakhon, R. Nutalai, D. Zhou, A. J. Mentzer, Y. Zhao, H. M. E. Duyvesteyn, C. López-Camacho, J. Slon-Campos, T. S. Walter, D. Skelly, S. A. Johnson, T. G. Ritter, C. Mason, S. A. Costa Clemens, F. Gomes Naveca, V. Nascimento, F. Nascimento, C. Fernandes da Costa, P. C. Resende, A. Pauvolid-Correa, M. M. Siqueira, C. Dold, N. Temperton, T. Dong, A. J. Pollard, J. C. Knight, D. Crook, T. Lambe, E. Clutterbuck, S. Bibi, A. Flaxman, M. Bittaye, S. Belij-Rammerstorfer, S. C. Gilbert, T. Malik, M. W. Carroll, P. Klenerman, E. Barnes, S. J. Dunachie, V. Baillie, N. Serafin, Z. Ditse, K. Da Silva, N. G. Paterson, M. A. Williams, D. R. Hall, S. Madhi, M. C. Nunes, P. Goulder, E. E. Fry, J. Mongkolsapaya, J. Ren, D. I. Stuart, G. R. Screaton, Reduced neutralization of SARS-CoV-2 B.1.617 by vaccine and convalescent serum. *Cell* **184**, 4220–4236.e13 (2021). doi:10.1016/j.cell.2021.06.020 Medline

33. W. F. Garcia-Beltran, E. C. Lam, K. St. Denis, A. D. Nitido, Z. H. Garcia, B. M. Hauser, J. Feldman, M. N. Pavlovic, D. J. Gregory, M. C. Poznansky, A. Sigal, A. G. Schmidt, A. J. Iafrate, V. Naranbhai, A. B. Balazs, Multiple SARS-CoV-2 variants escape neutralization by vaccine-induced humoral immunity. *Cell* **184**, 2372–2383.e9 (2021). doi:10.1016/j.cell.2021.03.013

34. L. Stamatatos, J. Czartoski, Y.-H. Wan, L. J. Homad, V. Rubin, H. Glantz, M. Neradilek, E. Seydoux, M. F. Jennewein, A. J. MacCamy, J. Feng, G. Mize, S. C. De Rosa, A. Finzi, M. P. Lemos, K. W. Cohen, Z. Moodie, M. J. McElrath, A. T. McGuire, mRNA vaccination boosts cross-variant neutralizing antibodies elicited by SARS-CoV-2 infection. *Science* **372**, 1413–1418 (2021). doi:10.1126/science.abg9175 Medline

35. C. J. Reynolds, C. Pade, J. M. Gibbons, D. K. Butler, A. D. Otter, K. Menacho, M. Fontana, A. Smit, J. E. Sackville-West, T. Cutino-Moguel, M. K. Maini, B. Chain, M.

Noursadeghi, T. Brooks, A. Semper, C. Manisty, T. A. Treibel, J. C. Moon, A. M. Valdes, Á. McKnight, D. M. Altmann, R. Boyton, Prior SARS-CoV-2 infection rescues B and T cell responses to variants after first vaccine dose. *Science* **372**, 1418–1423 (2021). doi:10.1126/science.abh1282 Medline

36. Z. Wang, F. Muecksch, D. Schaefer-Babajew, S. Finkin, C. Viant, C. Gaebler, H. H. Hoffmann, C. O. Barnes, M. Cipolla, V. Ramos, T. Y. Oliveira, A. Cho, F. Schmidt, J. Da Silva, E. Bednarski, L. Aguado, J. Yee, M. Daga, M. Turroja, K. G. Millard, M. Jankovic, A. Gazumyan, Z. Zhao, C. M. Rice, P. D. Bieniasz, M. Caskey, T. Hatziioannou, M. C. Nussenzweig, Naturally enhanced neutralizing breadth against SARS-CoV-2 one year after infection. *Nature* **595**, 426–431 (2021). doi:10.1038/s41586-021-03696-9 Medline

37. A. H. Ellebedy, K. J. L. Jackson, H. T. Kissick, H. I. Nakaya, C. W. Davis, K. M. Roskin, A. K. McElroy, C. M. Oshansky, R. Elbein, S. Thomas, G. M. Lyon, C. F. Spiropoulou, A. K. Mehta, P. G. Thomas, S. D. Boyd, R. Ahmed, Defining antigen-specific plasmablast and memory B cell subsets in blood after viral infection or vaccination. *Nat. Immunol.* **17**, 1226–1234 (2016). doi:10.1038/ni.3533 Medline

38. A. Nellore, E. Zumaquero, C. D. Scharer, R. G. King, C. M. Tipton, C. F. Fucile, T. Mi, B. Mousseau, J. E. Bradley, F. Zhou, P. A. Goepfert, J. M. Boss, T. D. Randall, I. Sanz, A. F. Rosenberg, F. E. Lund, Influenza-specific effector memory B cells predict long-lived antibody responses to vaccination in humans. bioRxiv 643973 [Preprint] (2021). https://doi.org/10.1101/643973.

39. M. Jahnmatz, G. Kesa, E. Netterlid, A. M. Buisman, R. Thorstensson, N. Ahlborg, Optimization of a human IgG B-cell ELISpot assay for the analysis of vaccine-induced B-cell responses. *J. Immunol. Methods* **391**, 50–59 (2013). doi:10.1016/j.jim.2013.02.009 Medline

40. S. Gouma, M. E. Weirick, M. J. Bolton, C. P. Arevalo, E. C. Goodwin, E. M. Anderson, C. M. McAllister, S. R. Christensen, D. Dunbar, D. Fiore, A. Brock, J. Weaver, J. Millar, S. DerOhannessian, T. U. C. P. Unit, I. Frank, D. J. Rader, E. J. Wherry, S. E. Hensley, Health care worker seromonitoring reveals complex relationships between common coronavirus antibodies and COVID-19 symptom duration. *JCI Insight* **6**, e150449 (2021). doi:10.1172/jci.insight.150449 Medline

41. K. W. Ng, N. Faulkner, G. H. Cornish, A. Rosa, R. Harvey, S. Hussain, R. Ulferts, C. Earl, A. G. Wrobel, D. J. Benton, C. Roustan, W. Bolland, R. Thompson, A. Agua-Doce, P. Hobson, J. Heaney, H. Rickman, S. Paraskevopoulou, C. F. Houlihan, K. Thomson, E. Sanchez, G. Y. Shin, M. J. Spyer, D. Joshi, N. O’Reilly, P. A. Walker, S. Kjaer, A. Riddell, C. Moore, B. R. Jebson, M. Wilkinson, L. R. Marshall, E. C. Rosser, A. Radziszewska, H. Peckham, C. Ciurtin, L. R. Wedderburn, R. Beale, C. Swanton, S. Gandhi, B. Stockinger, J. McCauley, S. J. Gamblin, L. E. McCoy, P. Cherepanov, E. Nastouli, G. Kassiotis, Preexisting and de novo humoral immunity to SARS-CoV-2 in humans. *Science* **370**, 1339–1343 (2020). doi:10.1126/science.abe1107 Medline

42. P. Nguyen-Contant, A. K. Embong, P. Kanagaiah, F. A. Chaves, H. Yang, A. R. Branche, D. J. Topham, M. Y. Sangster, S protein-reactive IGG and memory B cell production after

human SARS-CoV-2 infection includes broad reactivity to the S2 subunit. *mBio* **11**, 1–11 (2020). doi:10.1128/mBio.01991-20 Medline

43. J. Pallesen, N. Wang, K. S. Corbett, D. Wrapp, R. N. Kirchdoerfer, H. L. Turner, C. A. Cottrell, M. M. Becker, L. Wang, W. Shi, W.-P. Kong, E. L. Andres, A. N. Kettenbach, M. R. Denison, J. D. Chappell, B. S. Graham, A. B. Ward, J. S. McLellan, Immunogenicity and structures of a rationally designed prefusion MERS-CoV spike antigen. *Proc. Natl. Acad. Sci. U.S.A.* **114**, E7348–E7357 (2017). doi:10.1073/pnas.1707304114 Medline

44. K. S. Corbett, D. K. Edwards, S. R. Leist, O. M. Abiona, S. Boyoglu-Barnum, R. A. Gillespie, S. Himansu, A. Schäfer, C. T. Ziwawo, A. T. DiPiazza, K. H. Dinnon, S. M. Elbashir, C. A. Shaw, A. Woods, E. J. Fritch, D. R. Martinez, K. W. Bock, M. Minai, B. M. Nagata, G. B. Hutchinson, K. Wu, C. Henry, K. Bahl, D. Garcia-Dominguez, L. Ma, I. Renzi, W.-P. Kong, S. D. Schmidt, L. Wang, Y. Zhang, E. Phung, L. A. Chang, R. J. Loomis, N. E. Altaras, E. Narayanan, M. Metkar, V. Presnyak, C. Liu, M. K. Louder, W. Shi, K. Leung, E. S. Yang, A. West, K. L. Gully, L. J. Stevens, N. Wang, D. Wrapp, N. A. Doria-Rose, G. Stewart-Jones, H. Bennett, G. S. Alvarado, M. C. Nason, T. J. Ruckwardt, J. S. McLellan, M. R. Denison, J. D. Chappell, I. N. Moore, K. M. Morabito, J. R. Mascola, R. S. Baric, A. Carfi, B. S. Graham, SARS-CoV-2 mRNA vaccine design enabled by prototype pathogen preparedness. *Nature* **586**, 567–571 (2020). doi:10.1038/s41586-020-2622-0 Medline

45. T. J. C. Tan, M. Yuan, K. Kuzelka, G. C. Padron, J. R. Beal, X. Chen, Y. Wang, J. Rivera-Cardona, X. Zhu, B. M. Stadtmueller, C. B. Brooke, I. A. Wilson, N. C. Wu, Sequence signatures of two public antibody clonotypes that bind SARS-CoV-2 receptor binding domain. *Nat. Commun.* **12**, 3815 (2021). doi:10.1038/s41467-021-24123-7 Medline

46. H. L. Dugan, C. T. Stamper, L. Li, S. Changrob, N. W. Asby, P. J. Halfmann, N.-Y. Zheng, M. Huang, D. G. Shaw, M. S. Cobb, S. A. Erickson, J. J. Guthmiller, O. Stovicek, J. Wang, E. S. Winkler, M. L. Madariaga, K. Shanmugarajah, M. O. Jansen, F. Amanat, I. Stewart, H. A. Utset, J. Huang, C. A. Nelson, Y.-N. Dai, P. D. Hall, R. P. Jedrzejczak, A. Joachimiak, F. Krammer, M. S. Diamond, D. H. Fremont, Y. Kawaoka, P. C. Wilson, Profiling B cell immunodominance after SARS-CoV-2 infection reveals antibody evolution to non-neutralizing viral targets. *Immunity* **54**, 1290–1303.e7 (2021). doi:10.1016/j.immuni.2021.05.001 Medline

47. A. M. Rosenfeld, W. Meng, D. Y. Chen, B. Zhang, T. Granot, D. L. Farber, U. Hershberg, E. T. Luning Prak, Computational evaluation of B-cell clone sizes in bulk populations. *Front. Immunol.* **9**, 1472 (2018). doi:10.3389/fimmu.2018.01472 Medline

48. C. Gaebler, Z. Wang, J. C. C. Lorenzi, F. Muecksch, S. Finkin, M. Tokuyama, A. Cho, M. Jankovic, D. Schaefer-Babajew, T. Y. Oliveira, M. Cipolla, C. Viant, C. O. Barnes, Y. Bram, G. Breton, T. Hägglöf, P. Mendoza, A. Hurley, M. Turroja, K. Gordon, K. G. Millard, V. Ramos, F. Schmidt, Y. Weisblum, D. Jha, M. Tankelevich, G. Martinez-Delgado, J. Yee, R. Patel, J. Dizon, C. Unson-O'Brien, I. Shimeliovich, D. F. Robbiani, Z. Zhao, A. Gazumyan, R. E. Schwartz, T. Hatziioannou, P. J. Bjorkman, S. Mehandru, P. D. Bieniasz, M. Caskey, M. C. Nussenzweig, Evolution of antibody immunity to SARS-CoV-2. *Nature* **591**, 639–644 (2021). doi:10.1038/s41586-021-03207-w Medline

49. M. G. de Mattos Barbosa, H. Liu, D. Huynh, G. Shelley, E. T. Keller, B. T. Emmer, E. Sherman, D. Ginsburg, A. A. Kennedy, A. W. Tai, C. Wobus, C. Mirabeli, T. M. Lanigan, M. Samaniego, W. Meng, A. M. Rosenfeld, E. T. Luning Prak, J. L. Platt, M. Cascalho, IgV somatic mutation of human anti-SARS-CoV-2 monoclonal antibodies governs neutralization and breadth of reactivity. *JCI Insight* **6**, e147386 (2021). doi:10.1172/jci.insight.147386 Medline

50. D. Geers, M. C. Shamier, S. Bogers, G. den Hartog, L. Gommers, N. N. Nieuwkoop, K. S. Schmitz, L. C. Rijsbergen, J. A. T. van Osch, E. Dijkhuizen, G. Smits, A. Comvalius, D. van Mourik, T. G. Caniels, M. J. van Gils, R. W. Sanders, B. B. O. Munnink, R. Molenkamp, H. J. de Jager, B. L. Haagmans, R. L. de Swart, M. P. G. Koopmans, R. S. van Binnendijk, R. D. de Vries, C. H. GeurtsvanKessel, SARS-CoV-2 variants of concern partially escape humoral but not T cell responses in COVID-19 convalescent donors and vaccine recipients. *Sci. Immunol.* **6**, eabj1750 (2021). doi:10.1126/sciimmunol.abj1750

51. A. Tarke, J. Sidney, C. K. Kidd, J. M. Dan, S. I. Ramirez, E. D. Yu, J. Mateus, R. da Silva Antunes, E. Moore, P. Rubiro, N. Methot, E. Phillips, S. Mallal, A. Frazier, S. A. Rawlings, J. A. Greenbaum, B. Peters, D. M. Smith, S. Crotty, D. Weiskopf, A. Grifoni, A. Sette, Comprehensive analysis of T cell immunodominance and immunoprevalence of SARS-CoV-2 epitopes in COVID-19 cases. *Cell Rep. Med.* **2**, 100204 (2021). doi:10.1016/j.xcrm.2021.100204 Medline

52. A. Grifoni, D. Weiskopf, S. I. Ramirez, J. Mateus, J. M. Dan, C. R. Moderbacher, S. A. Rawlings, A. Sutherland, L. Premkumar, R. S. Jadi, D. Marrama, A. M. de Silva, A. Frazier, A. F. Carlin, J. A. Greenbaum, B. Peters, F. Krammer, D. M. Smith, S. Crotty, A. Sette, Targets of T Cell Responses to SARS-CoV-2 Coronavirus in Humans with COVID-19 Disease and Unexposed Individuals. *Cell* **181**, 1489–1501.e15 (2020). doi:10.1016/j.cell.2020.05.015 Medline

53. S. M. Kaech, E. J. Wherry, R. Ahmed, Effector and memory T-cell differentiation: Implications for vaccine development. *Nat. Rev. Immunol.* **2**, 251–262 (2002). doi:10.1038/nri778 Medline

54. D. D. Flannery, S. Gouma, M. B. Dhudasia, S. Mukhopadhyay, M. R. Pfeifer, E. C. Woodford, J. S. Gerber, C. P. Arevalo, M. J. Bolton, M. E. Weirick, E. C. Goodwin, E. M. Anderson, A. R. Greenplate, J. Kim, N. Han, A. Pattekar, J. Dougherty, O. Kuthuru, D. Mathew, A. E. Baxter, L. A. Vella, J. Weaver, A. Verma, R. Leite, J. S. Morris, D. J. Rader, M. A. Elovitz, E. J. Wherry, K. M. Puopolo, S. E. Hensley, SARS-CoV-2 seroprevalence among parturient women in Philadelphia. *Sci. Immunol.* **5**, eabd5709 (2020). doi:10.1126/sciimmunol.abd5709 Medline

55. M. Roederer, J. L. Nozzi, M. C. Nason, SPICE: Exploration and analysis of post-cytometric complex multivariate datasets. *Cytometry A* **79**, 167–174 (2011). doi:10.1002/cyto.a.21015 Medline

56. W. Meng, B. Zhang, G. W. Schwartz, A. M. Rosenfeld, D. Ren, J. J. C. Thome, D. J. Carpenter, N. Matsuoka, H. Lerner, A. L. Friedman, T. Granot, D. L. Farber, M. J. Shlomchik, U. Hershberg, E. T. Luning Prak, An atlas of B-cell clonal distribution in the human body. *Nat. Biotechnol.* **35**, 879–884 (2017). doi:10.1038/nbt.3942 Medline

57. L. Kuri-Cervantes, M. B. Pampena, W. Meng, A. M. Rosenfeld, C. A. G. Ittner, A. R. Weisman, R. S. Agyekum, D. Mathew, A. E. Baxter, L. A. Vella, O. Kuthuru, S. A. Apostolidis, L. Bershaw, J. Dougherty, A. R. Greenplate, A. Pattekar, J. Kim, N. Han, S. Gouma, M. E. Weirick, C. P. Arevalo, M. J. Bolton, E. C. Goodwin, E. M. Anderson, S. E. Hensley, T. K. Jones, N. S. Mangalmurti, E. T. Luning Prak, E. J. Wherry, N. J. Meyer, M. R. Betts, Comprehensive mapping of immune perturbations associated with severe COVID-19. *Sci. Immunol.* **5**, eabd7114 (2020). doi:10.1126/sciimmunol.abd7114 Medline

58. J. A. Vander Heiden, G. Yaari, M. Uduman, J. N. H. Stern, K. C. O'Connor, D. A. Hafler, F. Vigneault, S. H. Kleinstein, pRESTO: A toolkit for processing high-throughput sequencing raw reads of lymphocyte receptor repertoires. *Bioinformatics* **30**, 1930–1932 (2014). doi:10.1093/bioinformatics/btu138 Medline

59. J. Ye, N. Ma, T. L. Madden, J. M. Ostell, IgBLAST: An immunoglobulin variable domain sequence analysis tool. *Nucleic Acids Res.* **41**, W34–W40 (2013). doi:10.1093/nar/gkt382 Medline

60. A. M. Rosenfeld, W. Meng, E. T. Luning Prak, U. Hershberg, ImmuneDB: A system for the analysis and exploration of high-throughput adaptive immune receptor sequencing data. *Bioinformatics* **33**, 292–293 (2017). doi:10.1093/bioinformatics/btw593 Medline

61. A. M. Rosenfeld, W. Meng, E. T. Luning Prak, U. Hershberg, ImmuneDB, a Novel Tool for the Analysis, Storage, and Dissemination of Immune Repertoire Sequencing Data. *Front. Immunol.* **9**, 2107 (2018). doi:10.3389/fimmu.2018.02107 Medline

62. J. Huerta-Cepas, F. Serra, P. Bork, ETE 3: Reconstruction, Analysis, and Visualization of Phylogenomic Data. *Mol. Biol. Evol.* **33**, 1635–1638 (2016). doi:10.1093/molbev/msw046 Medline

63. B. D. Corrie, N. Marthandan, B. Zimonja, J. Jaglale, Y. Zhou, E. Barr, N. Knoetze, F. M. W. Breden, S. Christley, J. K. Scott, L. G. Cowell, F. Breden, iReceptor: A platform for querying and analyzing antibody/B-cell and T-cell receptor repertoire data across federated repositories. *Immunol. Rev.* **284**, 24–41 (2018). doi:10.1111/imr.12666 Medline

64. F. Vaida, L. Liu, Fast Implementation for Normal Mixed Effects Models With Censored Response. *J. Comput. Graph. Stat.* **18**, 797–817 (2009). doi:10.1198/jcgs.2009.07130 Medline

65. D. Mathew, J. R. Giles, A. E. Baxter, D. A. Oldridge, A. R. Greenplate, J. E. Wu, C. Alanio, L. Kuri-Cervantes, M. B. Pampena, K. D'Andrea, S. Manne, Z. Chen, Y. J. Huang, J. P. Reilly, A. R. Weisman, C. A. G. Ittner, O. Kuthuru, J. Dougherty, K. Nzingha, N. Han, J. Kim, A. Pattekar, E. C. Goodwin, E. M. Anderson, M. E. Weirick, S. Gouma, C. P. Arevalo, M. J. Bolton, F. Chen, S. F. Lacey, H. Ramage, S. Cherry, S. E. Hensley, S. A. Apostolidis, A. C. Huang, L. A. Vella, The UPenn COVID Processing Unit, M. R. Betts, N. J. Meyer, E. J. Wherry, Deep immune profiling of COVID-19 patients reveals distinct immunotypes with therapeutic implications. *Science* **369**, eabc8511 (2020). doi:10.1126/science.abc8511 Medline
